# JBP485 promotes tear and mucin secretion in ocular surface epithelia

**DOI:** 10.1038/srep10248

**Published:** 2015-05-21

**Authors:** Takahiro Nakamura, Yuiko Hata, Maho Nagata, Norihiko Yokoi, Shumpei Yamaguchi, Taiichi Kaku, Shigeru Kinoshita

**Affiliations:** 1 Department of Ophthalmology, Kyoto Prefectural University of Medicine, Kyoto, Japan; 2 Research Center for Inflammation and Regenerative Medicine, Doshisha University, Kyoto, Japan; 3 Japan Bio Products Co., Ltd, Tokyo, Japan

## Abstract

Dry eye syndrome (DES), a multifactorial disease of the tears and ocular surface, is one of the most common ocular disorders. Tear film contains ocular mucins and is essential for maintaining the homeostasis of the wet ocular surface. Since there are a limited number of clinical options for the treatment of DES, additional novel treatments are needed to improve the clinical results. In this study, we found that placental extract-derived dipeptide (JBP485) clearly promoted the expression and secretion of gel-forming mucin 5ac (Muc5ac) in rabbit conjunctival epithelium. JBP485 also elevated the expression level of cell surface-associated mucins (Muc1/4/16) in rabbit corneal epithelium. The Schirmer tear test results indicated that JBP485 induced tear secretion in the rabbit model. Moreover, JBP485 clinically improved corneal epithelial damage in a mouse dry eye model. Thus, our data indicate that JBP485 efficiently promoted mucin and aqueous tear secretion in rabbit ocular surface epithelium and has the potential to be used as a novel treatment for DES.

Dry eye syndrome (DES) is one of the most frequently encountered ocular diseases. Epidemiological investigations have reported that it is prevalent in up to one-third of the population in some countries, making it a subject of growing importance related to public health^1^,^2^,^3^. The International Dry Eye Workshop defined DES as a multifactorial disease of the tears and ocular surface that results in symptoms of discomfort, visual disturbance, and tear film instability, thus having the potential to damage the ocular surface^4^. In the clinical setting, DES is accompanied by increased tear-film osmolarity and ocular surface inflammation. Thus, a clear understanding of tear film dynamics and the pathogenesis of ocular inflammation is essential in order to successfully treat this type of disease.

The pre-ocular tear film is vital for the maintenance of a healthy, wet ocular surface. The film consists of an aqueous layer containing a high concentration of soluble mucin and an outermost lipid layer^5^. Recently, particular attention has been focused on the function of ocular mucins in dry eye disease. There are two types of ocular mucins that contribute, in different ways, to ocular surface homeostasis^6^,^7^: 1) cell surface-associated mucins (Muc1/4/16), which are expressed on ocular-surface epithelium, and 2) gel-forming mucin (Muc5ac), which is secreted by conjunctival goblet cells. Several lines of experimental and clinical evidence suggest that alterations in both cell surface-associated and gel-forming mucins occur in dry-eye-related ocular surface diseases^8^,^9^,^10^.

Currently, the first choice of treatment in cases of DES involves the use of tear supplementation with artificial tears. However, this therapeutic pathway provides only a partial replacement of the wet ocular surface components, and repeated instillation is often required. Sodium hyaluronate and autologous serum have also shown some clinical effectiveness in DES patients^11^,^12^,^13^. More recently, diquafosol sodium and rebamipide, which promote tear and mucin secretion, have been reported to be effective in improving the clinical symptoms of various dry eye diseases^14^,^15^. Although these types of eye-drop treatments give patients more therapeutic options, new treatments with enhanced clinical effectiveness are needed to improve the symptoms in these patients.

JBP485 (cyclo-trans-4-L-hydroxyprolyl-L-serine) is a dipeptide that was first isolated from placental extract (PE) as a mitogen for a kidney cell line, and it has subsequently been synthesized by chemical means^16^,^17^. Previous studies have demonstrated that JBP485 has anti-inflammatory and anti-apoptotic effects and that it exhibits protective properties for liver and gastrointestinal cells^17^,^18^,^19^,^20^,^21^. Recently, we discovered that JBP485 promotes the cell proliferation of corneal epithelium during wound healing (unpublished data). In that study, the findings of the corneal wound experiments unexpectedly revealed that rabbit eyes showed an increased level of tear secretion with JBP485. This led us to the interesting hypothesis that JBP485 might promote tear and mucin secretion in ocular surface epithelium and be a potential new treatment for DES.

The purpose of this present study was to investigate the effect of JBP485 on tear and mucin secretion in ocular surface epithelium. The findings of this study reveal for the first time that JBP485 dramatically promotes the secretion of not only ocular mucin, but also aqueous tears. In addition, we found that JBP485 repairs corneal epithelial damage in a mouse dry eye model. Thus, our data provides new insights into the function of JBP485 on the homeostasis of the ocular surface.

## Results

### JBP485 promotes the expression level of Muc5ac in conjunctival epithelium

**Figure 1:**
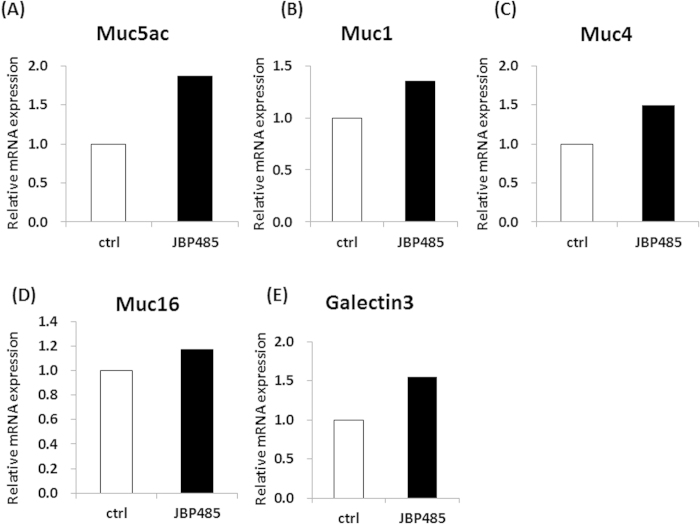
JBP485 promotes the expression level of mucin-related molecules in conjunctival epithelium. Relative expression of mucin-related molecules [mucin 5ac (Muc5ac), Muc1/4/16, and Galectin-3] in the control- and JBP485-treated conjunctival epithelial cells (A–E, 4 cell membrane filters, mixed). ctrl: control.

To investigate the effect of JBP485 on conjunctival epithelial cells, we first examined the expression level of secretory-mucin Muc5AC in rabbit conjunctival epithelial cells (CjECs) (*ex vivo* model). For this experiment, whole rabbit eyes were first incubated in the control solution [Hank’s balanced salt solution (HBSS)] and in JBP485 (100 μM) for 6 hours, and the CjECs were then collected using cell membrane filters from 4 different areas of the conjunctiva (the upper, lower, nasal, and temporal areas). The resultant 4-cell membrane filters were then mixed together and used for experiment. Interestingly, real-time polymerase chain reaction (PCR) tended to show an elevated level of Muc5ac expression in the JBP485-treated CjECs (Fig. 1A). In addition, we examined the expression of cell surface-associated mucin-related molecules (Muc1/4/16 and Galectin-3) and found that the expression levels of CjECs treated with JBP485 tended to be slightly increased compared to the controls (Fig. 1B–E). These findings suggest that JBP485 plays some critical role in inducing the expression of ocular surface mucins.

### JBP485 accelerates the secretion of Muc5ac in conjunctival epithelium

**Figure 2:**
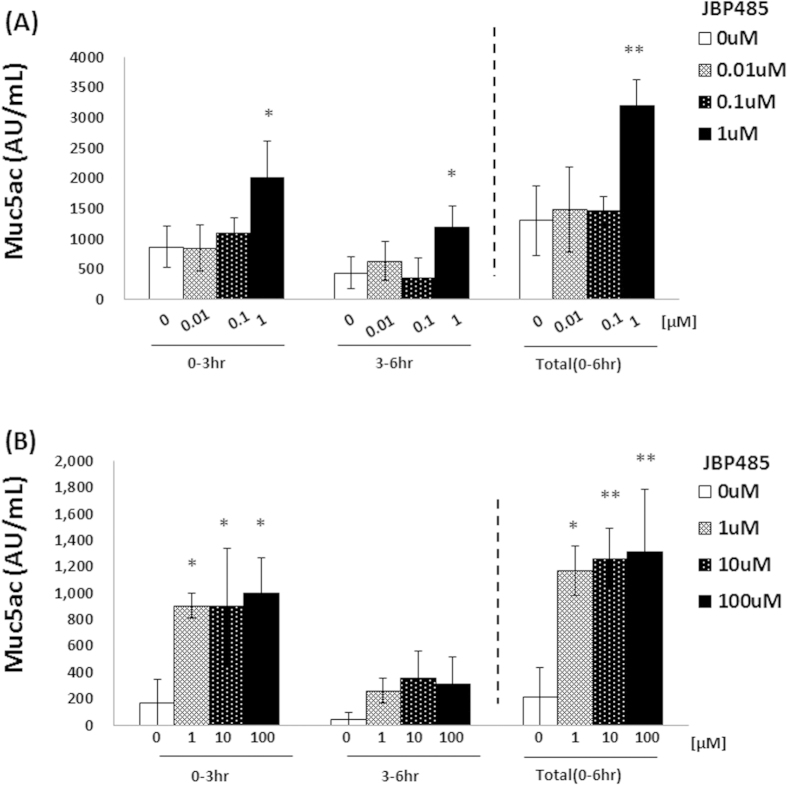
#JBP485 accelerates the secretion of Muc5ac in conjunctival epithelium (*ex vivo*). Protein levels of Muc5ac in conjunctival epithelium treated with JBP485 (for up to 6 hours) were examined by enzyme-linked immunosorbent assay. The concentration of JBP485 was less than 1 μM (A, n = 3) and more than 1 μM (B, n = 4). * *p *< 0.05, ** *p* < 0.01.

Muc5ac is a mucin that is secreted from conjunctival goblet cells. To elucidate whether JBP485 induces the secretion of Muc5ac in CjECs, quantification of Muc5ac was performed using enzyme-linked immunosorbent assay (ELISA). For this experiment, rabbit conjunctiva was obtained by use of surgical trephine (3 mm diameter) and then incubated in HBSS with or without JBP485 for up to 6 hours. At each time point (at 3 and 6 hours), supernatant was used for ELISA. After the first 3 hours of incubation, the expression level of Muc5ac in the samples treated with JBP485 (1 μM) was higher than that in the other samples (less than 1 μM) (Fig. 2A). The HBSS was then replaced with new HBBS, with or without JBP485 as above. After a further 3–6 hours of incubation, the expression level of Muc5ac in the JBP485-treated samples (1 μM) was higher than in the other samples, although the quantity of Muc5ac was less than that of at after 3 ours of incubation (Fig. 2A). We also examined the expression level of Muc5ac in samples treated with more than 1 μM of JBP485 and found that the expression patterns were similar to those in samples treated with 1 μM of JBP485 (Fig. 2B).

**Figure 3:**
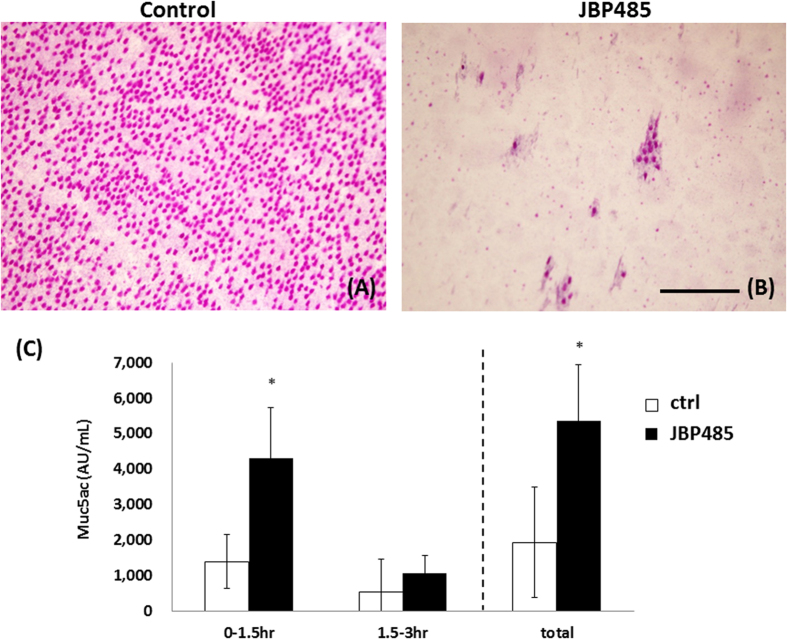
JBP485 accelerates the secretion of Muc5ac in conjunctival epithelium (*in vivo*). Representative Periodic Acid Schiff staining of conjunctival epithelium 30 minutes after a topical application of saline solution (**A**) and JBP485 (**B**). Protein levels of Muc5ac in the JBP485-treated (100 μM) conjunctival epithelium were examined by enzyme-linked immunosorbent assay (**C**). * *p* < 0.05 (n = 6). Scale bar, 100 μm.

To directly examine the secretion of Muc5ac from the CjECs, we performed Periodic Acid Schiff (PAS) staining (that can be used to detect secretory mucin) by use of impression cytology. Briefly, 30 minutes after the topical application of JBP485 or saline solution (control), the CjECs were obtained by use of impression cytology and the PAS staining was performed. We observed that PAS stained (+) cells were distributed in almost all areas of the eyes treated with saline solution, whereas the number of those cells (not goblet cells) decreased in the JBP485-treated eyes, indicating that JBP485 promotes mucin secretion (Fig. 3A, B).

Finally, we investigated whether or not a topical application of JBP485 on the eye would induce mucin secretion. Briefly, we first applied JBP485 or saline solution on the eyes 4-times daily for 3 days. Conjunctiva was then obtained from those eyes and incubated in HBSS for ELISA assay for up to 3 hours. As expected, the expression level of Muc5AC in the JBP485-treated samples (100 μM) was higher than that in the control samples (Fig. 3C). These findings clearly indicate that JBP485 accelerates the secretion of Muc5ac in CjECs.

### JBP485 elevates the expression level of cell surface-associated mucin in corneal epithelium in an *in vitro* model

**Figure 4:**
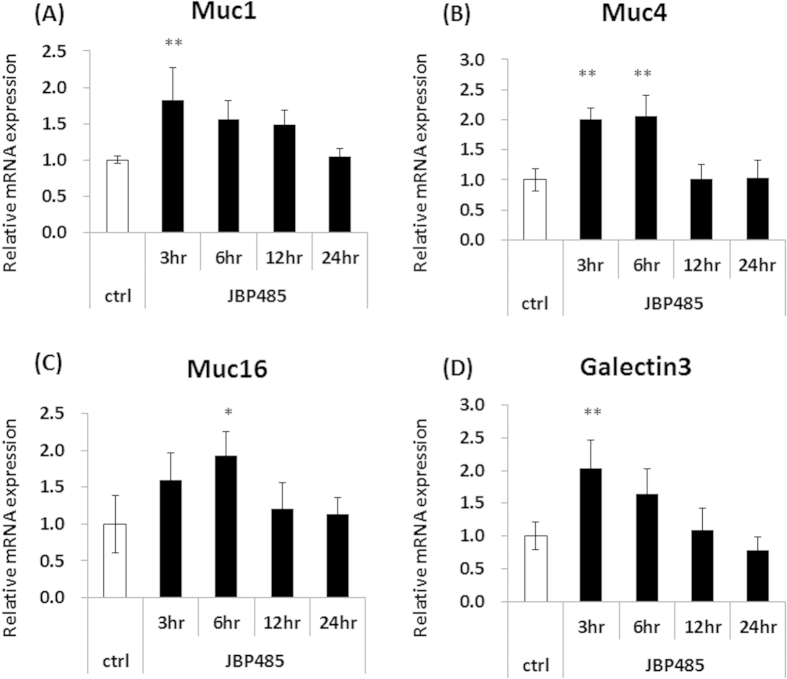
JBP485 elevates the expression level of cell surface-associated mucin in cultured corneal epithelium. Relative expression of mucin-related molecules (*Muc1/4/16* and *Galectin-3*) in the control- and JBP485-treated cultivated corneal epithelial cells (A–D). ctrl: control. * *p* < 0.05, ** *p* < 0.01 (n = 3).

To investigate the effect of JBP485 on corneal epithelial cells (CECs), we first examined the expression level of cell surface-associated mucin related molecules (Muc1/4/16 and Galectin-3) in rabbit primary cultured CECs. At 2  hours before sampling, we replaced the culture medium with DMEM/F12 and incubated the cells with or without JBP485 for each time point (3, 6, 12, and 24 hours). The cells were incubated with DMEM/F12 for 24 hours as a control. For the next assay, all samples were obtained simultaneously. Real-time PCR showed that after incubation with JBP485 for 3–6 hours, relative mRNA expression of Muc1/4/16 and Galectin-3 in the cultured CECs was higher than that in the controls (Fig. 4A–D). Moreover, we noticed that at 24 hours after incubation with JBP485, the relative expression levels were similar to those in the controls (Fig. 4A–D).

### *Ex vivo* model

**Figure 5:**
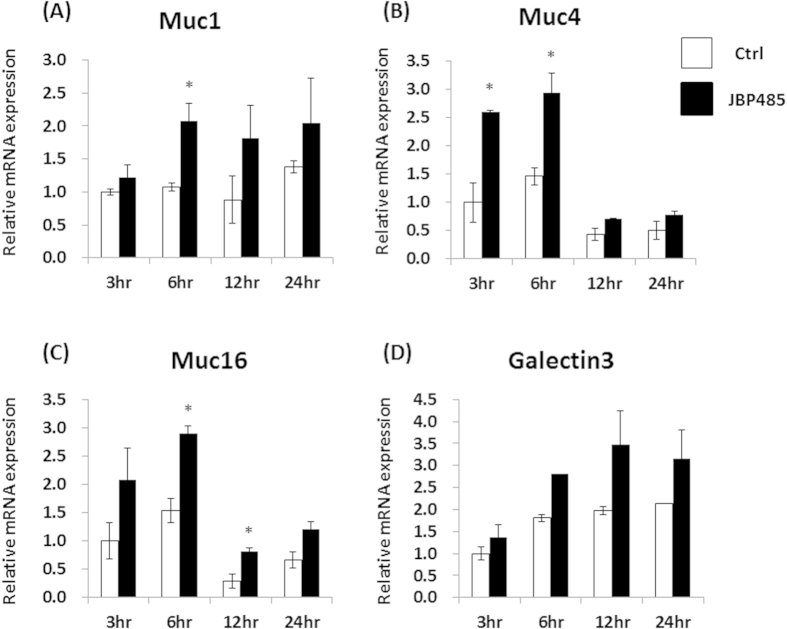
JBP485 elevates the expression level of cell surface-associated mucin in *ex vivo* corneal epithelium. Relative expression of mucin-related molecules (*Muc1/4/16* and *Galectin-3*) in the control- and JBP485-treated *ex vivo* corneal epithelium (A–D). ctrl: control. * *p* < 0.05 (n = 2).

To further investigate the effect of JBP485 on CECs, we examined the expression level of cell surface-associated mucin related molecules (Muc1/4/16 and Galectin-3) in *ex vivo* CECs. Briefly, whole rabbit eyes were incubated in JBP485 (100 uM), and CECs were then collected by mechanical scraping at 4 time points (after 3, 6, 12, and 24 hours of incubation). Real-time PCR showed that after incubation with JBP485 for 3–6 ours, the relative mRNA expression of Muc1/4/16 in *ex vivo* CECs tended to be higher than that in the control samples (Fig. 5A–D).

### *In vivo* model

**Figure 6:**
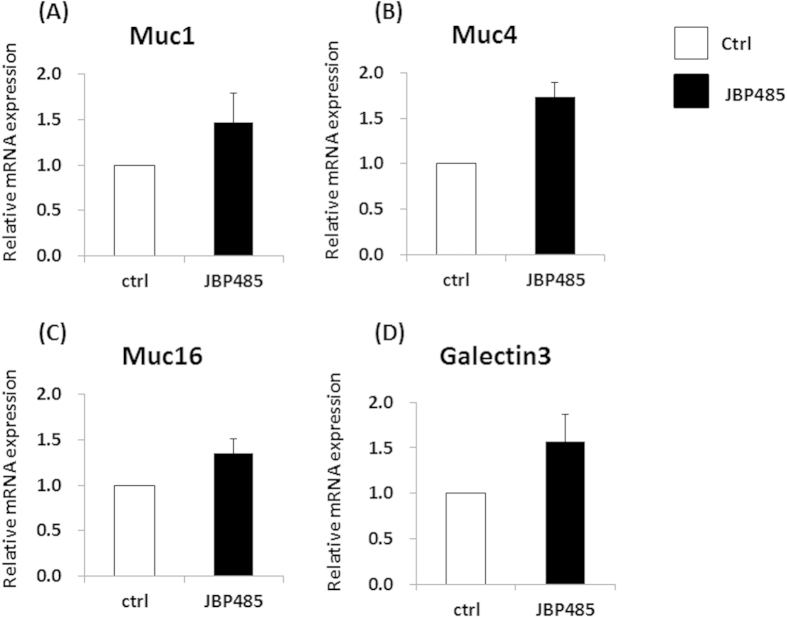
JBP485 elevates the expression level of cell surface-associated mucin in *in vivo* corneal epithelium. Relative expression of mucin-related molecules (*Muc1/4/16* and *Galectin-3*) in the control- and JBP485-treated *in vivo* corneal epithelium (A–D). ctrl: control (n = 3).

Finally, the effect of JBP485 on corneal CECs was investigated by examining the expression level of cell surface-associated mucin related molecules (Muc1/4/16 and Galectin-3) in *in vivo* CECs. Briefly, JBP485 (100 μM) or saline solution was topically applied 4-times daily for 12 days, and then the CECs were obtained by mechanical scraping. As expected, the expression levels of Muc1/4/16 and Galectin-3 in the JBP485-treated CECs tended to be higher than in the controls (Fig. 6A–D). These findings clearly indicate that JBP485 promotes the expression level of cell surface-associated mucins in CECs.

### JBP485 induces tear secretion in a rabbit model

**Figure 7:**
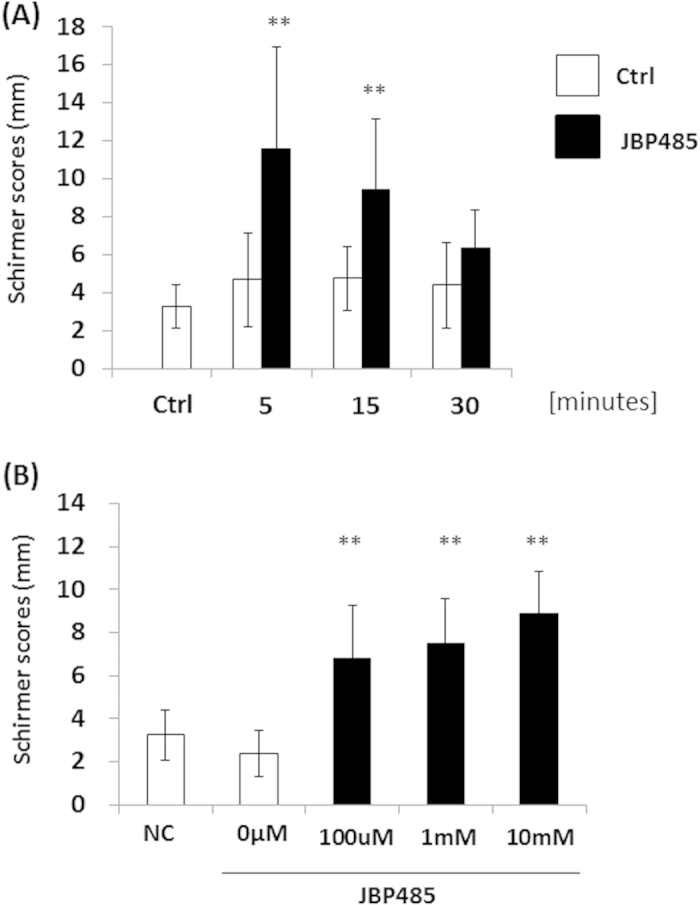
JBP485 induces tear secretion in a rabbit model. Schirmer test scores at different time points (up to 30 minutes) after the topical application of JBP485 solution (A, n = 8–9). Schirmer test scores for different concentrations of JBP485 after the topical application of JBP485 solution (B, n = 8-10). ** *p* < 0.01.

To investigate the effect of JBP485 on aqueous tear-fluid secretion, we performed the Schirmer test using a rabbit model. Topical application of JBP485 (100 μM) significantly increased aqueous tear volume from 5 minutes post application, and that effect gradually decreased between 5 and 30 minutes post application (Fig. 7A). Next, we examined the effect of different concentrations of JBP485 and found that JBP485 clearly increased aqueous tear volume in a concentration-dependent manner (up to 10 mM) in rabbit eyes (Fig. 7B). These results confirm that JBP485 accelerates tear secretion in a rabbit model.

### JBP485 repairs corneal epithelial damage in a mouse dry eye model

**Figure 8:**
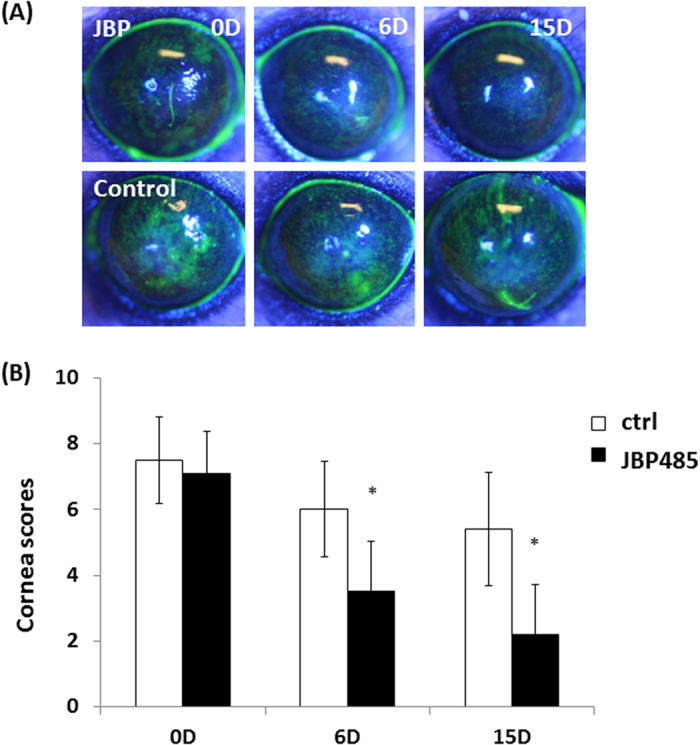
JBP485 repairs corneal epithelial damage in a mouse dry eye model. Fluorescent slit-lamp photographs of mouse eyes with or without JBP485 treatment for up to 15 days (**A**). Corneal fluorescein grading score at each time point after the topical application of JBP485 (**B**). * *p* < 0.05 (n = 10).

Following our findings demonstrating that JBP485 accelerates not only mucin secretion but also aqueous tear production, we investigated whether or not a topical application of JBP485 might be useful for the treatment of dry eye disease. Due to the fact that a rabbit dry eye model is difficult to establish and is too invasive and unstable to be used for evaluation, we used a simple mouse model of experimentally induced dry eye disease. Before the experiment, we confirmed that all mice showed reduced tear volume and resultant superficial punctate keratitis (SPK) on the cornea (Fig. 8A). Six days after the topical application of JBP485 (100 μM), SPK on the mice corneas were reduced in comparison to the controls (Fig. 8A). Fifteen days after the topical application of JBP485, SPK on the mice corneas was further reduced and corneal damage had clearly improved (Fig. 8A). In fact, we found that his effect was statistically significant (Fig. 8B). These findings suggest that JBP485 is useful in treating experimental dry eye conditions.

## Discussion

In the current information-based society, most external information is gained through eyesight, thus leading us to we overwork our eyes. Consequently, dry eye has become one of the most common ocular diseases, and particular attention has recently been focused on the treatment of DES. Cyclosporine ophthalmic emulsion, which has an anti-inflammatory effect, is the major drug used for the treatment of dry eye in the United States^22^. Diquafosol sodium and rebamipide promote the secretion of mucin and tears, and they are quickly becoming the primary eye drops used for the treatment of DES in Japan^14^,^15^. Although these treatments are clinically effective for many patients, alternative treatment options are still needed. The results of this study demonstrate for the first time that JBP485 derived from PE clearly promotes the expression and secretion of mucin and tears and that it has the potential to be used as a novel treatment for dry eye.

Ocular mucins are present on the most apical surface of all mucosal epithelium in either gel-forming or cell surface-associated forms. Gel-forming secreted mucin (Muc5ac) has no transmembrane spanning domains and is specialized in various kinds of epithelial glands^10^. Our results demonstrate that JBP485 dramatically accelerates the secretion of Muc5ac from conjunctival goblet cells. In the clinical setting, the reaction time of a drug is critical for evaluating its usefulness. From our PAS staining and ELISA experiments, we found that the effect of JBP485 on conjunctival goblet cells was very fast (within 30 minutes), and these results are similar to the findings in a previous report that the topical application of INS365 (P2Y2 receptor agonist, Diquafosol sodium) dose-dependently decreased the PAS staining area up to 15 minutes^23^. Therefore, JBP485 might be a good candidate for topical application on the ocular surface. We posit that the topical administration of JBP485 must be correlated with *in vivo* analyses of Muc5ac mRNA in the conjunctiva and Muc5ac protein in tears. Unfortunately, based on our current findings, we were unable to fully demonstrate these mechanisms, and further investigation is needed to clarify these points.

Cell surface-associated mucins have a single transmembrane domain including a short cytoplasmic tail and a large glycosylated extracellular domain; they are observed in the glycocalyx of apical cell membranes of the epithelium^10^. Currently, 10 cell surface-associated mucins have been reported, and each may have a different biological function. Among them, Muc1, Muc4, and Muc16 are reportedly expressed in the ocular surface epithelium^24^,^25^,^26^,^27^. It has been proposed that these molecules have multiple functions, including protection, barrier function and lubrication of the ocular surface epithelium, cell signaling (EGF and Wnt), and hypothetically, osmosensors^28^,^29^. DES is reportedly associated with alterations in mucin expression, and expression of Muc1 and Muc16 are known to be decreased in patients with Sjogren and non-Sjogren syndrome, respectively^8^,^9^. Our *in vitro, ex vivo,* and *in vivo* experiments clearly showed that JBP485 elevates the expression level of these cell surface-associated mucins, suggesting that JBP485 might have the ability to normalize the apical surface of corneal and conjunctival epithelium.

Aqueous tears are vital for maintaining ocular-surface homeostasis. To the best of our knowledge, diquafosol sodium is the only eye drop that can be used to increase tear secretion^14^. Thus, additional clinical options need to be developed for the treatment of dry eye. Our results demonstrate that JBP485 accelerates tear secretion in a rabbit model in a dose-dependent manner. Interestingly, 5 minutes after the topical application of JBP485, the rabbit eyes clearly showed an increased tear volume level, and throughout the *in vivo* experiments we never observed any adverse effects such as injection, neovascularization, or inflammation. This indicates that the effect of JBP485 on the ocular surface is both safe and prompt. We theorize that the mechanism by which the level of tears is improved is an important aspect when evaluating the clinical efficacy of JBP 485. For instance, we investigated the expression of the P2Y2 receptor with or without JBP485 treatment, and found that there were no significant differences between them (data not shown). Unfortunately, we do not precisely know its mechanism, and extensive further experiments are needed to clarify these points.

JBP485 was first found as a defined hydrolysate from human placenta. Extensive and accumulating investigations demonstrate that human placenta has multiple biological functions, including anti-inflammatory effects, anti-platelet aggregation, protection and regeneration of hepatocytes, regulation of hormonal balance, modulation of immune reaction, and promotion of wound healing^30^,^31^,^32^,^33^,^34^. JBP485 has also consistently shown the same kinds of biological effects. However, no reports exist regarding the biological effects of JBP485 on the secretion of mucin and aqueous tear in the ocular surface epithelium. Goblet cells and glandular tissues exist in great numbers throughout the body. Therefore, our findings might provide new insight into the therapeutic effects of JBP485 not only on ocular surface epithelium but also on other related tissues and organs.

Our investigation demonstrates that JBP485 promotes the expression and secretion of mucin and aqueous tear, but using a proper animal model that simulates dry eye conditions is an important next step for the evaluation of future therapeutic treatments of dry eye diseases. Numerous mouse dry eye models have been developed to reflect the pathogenesis involved in dry eye syndrome. We decided to use a model employing the excision of the exorbital lacrimal gland simply because it is quick and easy to do. Importantly, JBP485 successfully treated the damaged corneal surface in the mouse dry eye model, providing direct evidence of the clinical efficacy of JBP485 in *in vivo* situations. However, we have not fully demonstrated that JBP485 also promotes the tear and mucin secretion in mouse ocular surface epithelia and further investigations using mouse cornea and conjunctival epithelia are needed to clarify these points.

Regarding the pathogenesis of dry eye disease, tear hyperosmolarity, tear film instability, and inflammation have been identified as core mechanisms associated with the development of the disease. Ocular inflammation may initially be accompanied by an increase in blinking and the tear reflex. This may result in a reduction in corneal sensation, leading to increased evaporation and tear-film instability. Thus, it is important to control ocular inflammation in patients with DES. Previous studies have demonstrated that JBP485 exhibits anti-inflammatory effects in the liver and intestine^17^,^18^,^19^,^20^,^21^, and our recent findings show that JBP485 reduces inflammation in an experimental model of cultured CECs (data not shown), thus providing additional evidence that JBP485 might have advantages over the current available treatments for dry eye disease.

In conclusion, the findings of our investigation show that PE-derived JBP485 efficiently accelerates mucin and aqueous tear secretion in ocular surface epithelium. Moreover, JBP485 was found to be effective for the treatment of corneal damage in a mouse dry eye model. Although further investigation is needed to elucidate the molecular mechanisms of its biological function, our data provides new insights into the therapeutic potential of JBP485 in the most common type of dry eye disease.

## Materials and Methods

### Reagent

The JBP485 used in this study was provided by Japan Bioproducts Industry Co., Ltd. (Tokyo, Japan)^16^,^17^,^18^,^19^,^20^,^21^. JBP485 powder was dissolved by the use of warmed saline (Otsuka Pharmaceutical Co., Ltd., Tokyo, Japan) into a 50 mM stock solution at the temperature of 50–60 °C. The solution was treated with filtration sterilization (0.22 μm), and then diluted and adjusted to the required concentration with saline for instillation or for use as a culture medium.

### Animals

All animals used in this study were treated in accordance with the ARVO Statement on the Use of Animals in Ophthalmic and Vision Research. All experimental procedures were approved by the Committee for Animal Research at Kyoto Prefectural University of Medicine. Samples were taken from adult albino rabbits (2–2.5 kg in weight) under general anesthesia induced by an intramuscular injection of xylazine hydrochloride (5 mg/ml) and ketamine hydrochloride (50 mg/ml).

### Cell culture

Japanese white rabbit eyes were purchased from Funakoshi Corporation (Tokyo, Japan). Rabbit corneal epithelial cells were prepared and cultured according to a previously reported method^35^. Briefly, the corneas were removed from the eyes, and the half-layers of the endothelial side were then mechanically removed by use of surgical scissors. The remaining corneal tissues were transferred to a minimum essential keratinocyte serum-free medium (Life Technologies, Carlsbad, CA) containing dispase I (Wako Pure Chemical Industries, Osaka, Japan). The tissues were then incubated at 4 °C for 24 hours and at 37 °C for 1 hour. The resultant corneal epithelium was then peeled and further incubated with TrypLE^TM^ Select (Life Technologies) trypsin replacement enzyme at 4 °C for 30 minutes and at 37 °C for 5 minutes to dissociate the cells. The prepared rabbit CECs were suspended in CnT-20 medium (CELLnTEC, Santa Cruz, CA) and plated in collagen-coated tissue culture plates (AGC Techno Glass Co., Ltd., Shizuoka, Japan).

### Real-time PCR

Real-time PCR was performed following our previously described method^36^. All samples were homogenized in lysis buffer (Buffer RLT; QIAGEN, Inc., Valencia, CA), and total RNA was eluted by use of the RNeasy^®^ Mini Kit (QIAGEN) according to the manufacturer’s instructions. The relative abundance of transcripts was detected by use of SYBR^®^ Green PCR Master Mix (Applied Biosystems, Inc., Foster City, CA) according to the manufacturer’s instructions. The primers that were used are shown in Supplemental Table 1. Sequence homology data for mucins and galectin in humans and rabbits are shown in Supplemental Information 1–5. We confirmed that our designed primers properly amplified the objective sequences using PCR and sequencing (Supplemental Fig. 1).

### ELISA

Quantification of the mucin 5ac (Muc5ac) expression level was performed using ELISA by our previously reported method^37^. Each sample was incubated with HBSS (1ml/well, 24-well plates) at 37 °C. The resultant supernatant solution was incubated overnight at 40 °C. The wells were washed 3 times with 0.05% tris-buffered saline with tween20 (TBST) and incubated with 1% BSA for 1 hour at room temperature (RT). Next, the wells were incubated with anti-Muc5ac antibody (X100, Thermo Fisher Scientific, Inc., Waltham, MA) (Supplemental Fig. 2) for 1 hour at RT. The wells were then washed 3 times with TBST and incubated with secondary antibody (sheep anti-mouse IgG-HRP; GE Healthcare, Little Chalfont, UK) for 1 hour at RT. Finally, the wells were incubated with Tetramethylbenzidine (Sigma-Aldrich, St. Louis, MO) for 30 minutes at RT. The reaction was stopped by adding 0.5 M sulfuric acid solution (Nacalai Tesque Co., Kyoto, Japan). The absorbances were read at 450 nm using a microplate reader. For the experiment, the samples were run in triplicate. We set up the standard curve using an optional control well. The value shown in “Muc5ac (AU/mL)” was then calculated by the established standard curve [y = 0.0071x + 0.2727 (R^2^ = 0.9937) (Fig. 2A), y = 0.026x + 0.326 (R^2^ = 0.9969) (Fig. 2B)].

### Impression cytology and PAS staining

After administration of the JBP485 and saline solution, membrane filters (Millipore GSWP, Billerica, MA) were placed directly on the rabbit conjunctiva for 30 seconds. The resultant membranes were then fixed in 10% formalin and oxidized in 0.5% periodic acid solution for 10 minutes. After washing with distilled water, the membranes were placed in Schiff reagent for 15 minutes. After counterstaining in hematoxylin for 1 minute, the samples were dehydrated and mounted under a coverslip using a mounting medium.

### Tear fluid secretion

To measure tear secretion, we performed the Schirmer tear test (Sterilized Tear Production Measuring Strip; Showa, Tokyo, Japan) using rabbit eyes (Japanese albino rabbits, 2.5–3 kg in weight, n = 8~10)^38^. First, topical anesthesia with 0.4% oxybuprocaine hydrochloride was applied onto the rabbit eyes. Next, at 5 minutes after the administration of 50 μL JBP485 (100 μM) or saline solution, paper strips for the Schirmer tear test were inserted into the temporal conjunctival sac of the lower eyelid for 1 minute to measure tear production. The test paper was then removed from the lower palpebral conjunctiva and the amount of moisture was measured. All tests were performed after the rabbits were attached to a fixation device and had grown quiet.

### Mouse dry eye model

Excision of the exorbital lacrimal gland was performed on C57BL/6 mice at 6 weeks before examination (n = 20 eyes)^39^. Before starting the experiment, we confirmed that tear production in the mice was significantly decreased after gland excision and that SPK was clearly observed. JBP485 solution (100 μM, n = 10 eyes) and saline solution (n = 10 eyes) was topically applied on the mice eyes 4 times daily. To evaluate dry eye symptoms, fluorescein staining of the mice corneas was performed at 0, 6 and 15 days after treatment. A corneal fluorescein grading score (0: none, 1: mild, 2: moderate and 3: severe) was assigned at 3 different corneal areas (the upper, middle, and lower areas). A score of 9 points indicated severe SPK.

### Statistical analysis

The statistical significance (*P*-value) in mean values of the two-sample comparison was determined by use of the Student’s *t*-test. The statistical significance in the comparison of multiple sample sets was analyzed by use of the Dunnett’s multiple-comparisons test. Values shown on the graphs represent the mean ± SEM.

## Author Contributions

T.N.: conception and design; T.N., Y.H. and M.N.: collection and/or assembly of data; T.N., S.Y., T.K., N.Y. and S.K.: data analysis and interpretation; T.N.: writing manuscript.

## Additional Information

**How to cite this article**: Nakamura, T. *et al.* JBP485 promotes tear and mucin secretion in ocular surface epithelia. *Sci. Rep.* **5**, 10248; doi: 10.1038/srep10248 (2015).

## Supplementary Material

Supporting Information

## References

[b1] Peral A., Dominguez-Godinez C. O., Carracedo G. & Pintor J. Therapeutic targets in dry eye syndrome. Drug News Perspect 21, 166–76 (2008). 18560615

[b2] Gayton J. L. Etiology, prevalence, and treatment of dry eye disease. Clin. Ophthalmol. 3, 405–12 (2009). 19688028 10.2147/opth.s5555 PMC2720680

[b3] Tavares F. de P., Fernandes R. S., Bernardes T. F., Bonfioli A. A. & Soares E. J. Dry eye disease. Semin. Ophthalmol. 25, 84–93 (2010). 20590418 10.3109/08820538.2010.488568

[b4] The definition and classification of dry eye disease: report of the Definition and Classification Subcommittee of the International Dry Eye WorkShop (2007). Ocul. Surf. 5, 75–92 (2007). 17508116 10.1016/s1542-0124(12)70081-2

[b5] Lemp M.A. Advances in understanding and managing dry eye disease. Am. J. Ophthalmol. 146, 350–356 (2008). 18599017 10.1016/j.ajo.2008.05.016

[b6] Gipson I. K. The ocular surface: the challenge to enable and protect vision: the Friedenwald lecture. Invest Ophthalmol. Vis. Sci. 48, 4390; 4391–8 (2007). 10.1167/iovs.07-0770 PMC2886589 17898256

[b7] Corfield A., Carrington S., Hicks S., Berry M. & Ellingham R. Ocular mucins: purification metabolism and function. Progress in Retinal and Eye Research 16, 627 - 56 (1997).

[b8] Danjo Y. *et al.* Alteration of mucin in human conjunctival epithelia in dry eye. Invest Ophthalmol. Vis. Sci. 39, 2602–9 (1998). 9856770

[b9] Argueso P. *et al.* Decreased levels of the goblet cell mucin MUC5AC in tears of patients with Sjogren syndrome. Invest Ophthalmol. Vis. Sci. 43, 1004–11 (2002). 11923240

[b10] Gipson I. K., Hori Y. & Argueso P. Character of ocular surface mucins and their alteration in dry eye disease. Ocul. Surf. 2, 131–48 (2004). 17216084 10.1016/s1542-0124(12)70149-0

[b11] Shimmura S. *et al.* Sodium hyaluronate eyedrops in the treatment of dry eyes. Br. J. Ophthalmol. 79, 1007–11 (1995). 8534643 10.1136/bjo.79.11.1007 PMC505317

[b12] Tsubota K. *et al.* Treatment of dry eye by autologous serum application in Sjogren’s syndrome. Br. J. Ophthalmol. 83, 390–5 (1999). 10434857 10.1136/bjo.83.4.390 PMC1723012

[b13] Condon P. I. *et al.* Double blind, randomised, placebo controlled, crossover, multicentre study to determine the efficacy of a 0.1% (w/v) sodium hyaluronate solution (Fermavisc) in the treatment of dry eye syndrome. Br. J. Ophthalmol. 83, 1121–4 (1999). 10502570 10.1136/bjo.83.10.1121 PMC1722832

[b14] Takamura E., Tsubota K., Watanabe H. & Ohashi Y. A randomised, double-masked comparison study of diquafosol versus sodium hyaluronate ophthalmic solutions in dry eye patients. Br. J. Ophthalmol. 96, 1310–5 (2012). 22914501 10.1136/bjophthalmol-2011-301448 PMC3463860

[b15] Kinoshita S. *et al.* Rebamipide (OPC-12759) in the treatment of dry eye: a randomized, double-masked, multicenter, placebo-controlled phase II study. Ophthalmology 119, 2471–8 (2012). 23009892 10.1016/j.ophtha.2012.06.052

[b16] Yagi A., Nagao M., Okamura N., Ishizu T. & Itoh H. Effect of cyclo (trans-4-L-hydroxyprolyl-L-serine) from hydrolysate of human placenta on baby hamster kidney -21/C-13 cells. Natural medicines 52, 156–9 (1998).

[b17] Liu K. X. *et al.* Hydroxyprolylserine derivatives JBP923 and JBP485 exhibit the antihepatitis activities after gastrointestinal absorption in rats. J. Pharmacol. Exp. Ther. 294, 510–5 (2000). 10900226

[b18] Wu J. *et al.* Protective effect of JBP485 on concanavalin A-induced hepatocyte toxicity in primary cultured rat hepatocytes. Eur. J. Pharmacol. 589, 299–305 (2008). 18571156 10.1016/j.ejphar.2008.04.066

[b19] Yang T. *et al.* Protective effect of JBP485 on concanavalin A-induced liver injury in mice. J. Pharm. Pharmacol. 61, 767–74 (2009). 19505367 10.1211/jpp.61.06.0009

[b20] Cang J. *et al.* Pharmacokinetics and mechanism of intestinal absorption of JBP485 in rats. Drug. Metab. Pharmacokinet 25, 500–7 (2010). 20877133 10.2133/dmpk.dmpk-10-rg-045

[b21] Wang W. *et al.* Effects of JBP485 on the expression and function of PEPT1 in indomethacin-induced intestinal injury in rats and damage in Caco-2 cells. Peptides 32, 946–55 (2011). 21310202 10.1016/j.peptides.2011.01.031

[b22] Laibovitz R. A., Solch S., Andriano K., O’Connell M. & Silverman M. H. Pilot trial of cyclosporine 1% ophthalmic ointment in the treatment of keratoconjunctivitis sicca. Cornea 12, 315–23 (1993). 8339560 10.1097/00003226-199307000-00007

[b23] Fujihara T., Murakami T., Nagano T., Nakamura M. & Nakata K. INS365 suppresses loss of corneal epithelial integrity by secretion of mucin-like glycoprotein in a rabbit short-term dry eye model. J. Ocul. Pharmacol. Ther. 18, 363–70 (2002). 12222766 10.1089/10807680260218524

[b24] Inatomi T., Spurr-Michaud S., Tisdale A. S. & Gipson I. K. Human corneal and conjunctival epithelia express MUC1 mucin. Invest Ophthalmol. Vis. Sci. 36, 1818–27 (1995). 7635656

[b25] Inatomi T. *et al.* Expression of secretory mucin genes by human conjunctival epithelia. Invest Ophthalmol. Vis. Sci. 37, 1684–92 (1996). 8675412

[b26] Argueso P., Spurr-Michaud S., Russo C. L., Tisdale A. & Gipson I. K. MUC16 mucin is expressed by the human ocular surface epithelia and carries the H185 carbohydrate epitope. Invest Ophthalmol. Vis. Sci. 44, 2487–95 (2003). 12766047 10.1167/iovs.02-0862

[b27] Pflugfelder S. C. *et al.* Detection of sialomucin complex (MUC4) in human ocular surface epithelium and tear fluid. Invest Ophthalmol. Vis. Sci. 41, 1316–26 (2000). 10798646

[b28] Hollingsworth M. A. & Swanson B. J. Mucins in cancer: protection and control of the cell surface. Nat. Rev. Cancer 4, 45–60 (2004). 14681689 10.1038/nrc1251

[b29] Govindarajan B. & Gipson I. K. Membrane-tethered mucins have multiple functions on the ocular surface. Exp. Eye. Res. 90, 655–63 (2010). 20223235 10.1016/j.exer.2010.02.014 PMC2893012

[b30] Sur T. K., Biswas T. K., Ali L. & Mukherjee B. Anti-inflammatory and anti-platelet aggregation activity of human placental extract. Acta. Pharmacol. Sin. 24, 187–92 (2003). 12546729

[b31] Liu K. X., Kato Y., Kaku T. & Sugiyama Y. Human placental extract stimulates liver regeneration in rats. Biol. Pharm. Bull. 21, 44–9 (1998). 9477167 10.1248/bpb.21.44

[b32] Kong M. H. *et al.* Effect of human placental extract on menopausal symptoms, fatigue, and risk factors for cardiovascular disease in middle-aged Korean women. Menopause. 15, 296–303 (2008). 18090035 10.1097/gme.0b013e3181405b74

[b33] Banerjee K. K., Bishayee A. & Chatterjee M. Effects of human placental extract on brain monoamines and monoamine oxidase activity in rats. Tohoku. J. Exp. Med. 176, 17–24 (1995). 7482515 10.1620/tjem.176.17

[b34] Tiwary S. K. *et al.* Effect of placental-extract gel and cream on non-healing wounds. J. Wound Care 15, 325–8 (2006). 16869202 10.12968/jowc.2006.15.7.26937

[b35] Nakamura T. *et al.* Long-term phenotypic study after allogeneic cultivated corneal limbal epithelial transplantation for severe ocular surface diseases. Ophthalmology 117, 2247–2254 (2010). 20673588 10.1016/j.ophtha.2010.04.003

[b36] Nakamura T. *et al.* LRIG1 inhibits STAT3-dependent inflammation to maintain corneal homeostasis. J. Clin. Invest 124, 385–97 (2014). 24316976 10.1172/JCI71488 PMC3871248

[b37] Kobayashi M. *et al.* Ocular surface reconstruction with a tissue-engineered nasal mucosal epithelial cell sheet for the treatment of severe ocular surface diseases. Stem Cells Transl. Med. Nov. 19. Epub ahead of print (2014). 10.5966/sctm.2014-0169 PMC4275014 25411478

[b38] Abrams K. L., Brooks D. E., Funk R. S. & Theran P. Evaluation of the Schirmer tear test in clinically normal rabbits. *Am. J. Vet. Res.* 51, 1912–3 (1990). 2085216

[b39] Shinomiya K. *et al.* Usefulness of a Dry-eye Mouse Model Produced by Exorbital Lacrimal Gland Excision and Analysis of the Increase of Caspase-1 Independent IL-1 β in the Tear Fluid of Those Mice. Invest Ophthalmol. Vis. Sci. 53, e-abstract 2335. (2012).

