# Supplementary material for: JBP485 promotes tear and mucin secretion in ocular surface epithelia
Source: Sci Rep. 2015 May 21;5:10248. doi: 10.1038/srep10248 (PMC4440520; doi:10.1038/srep10248)
Supplement: Supporting Information [file srep10248-s1.pdf]

Supplementary Information

JBP485 promotes tear and mucin secretion in ocular surface epithelia

Takahiro Nakamura, Yuiko Hata, Maho Nagata, Norihiko Yokoi, Shumpei Yamaguchi, Taiichi Kaku, Shigeru Kinoshita

Figure S1.

PCR for Muc1/Muc4/Muc16/Muc5ac and Galectin 3 in rabbit conjunctiva and cornea.

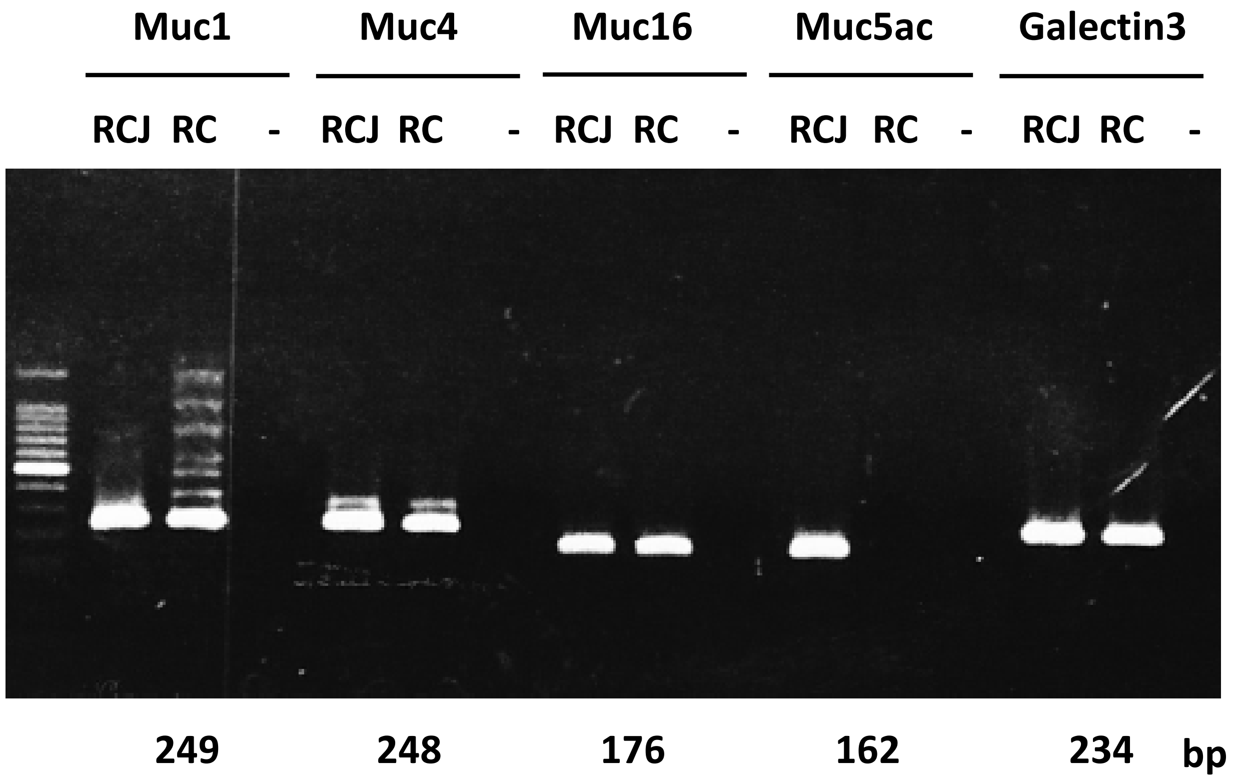

RCJ: Rabbit Conjunctiva

RC: Rabbit Cornea

-: Negative control

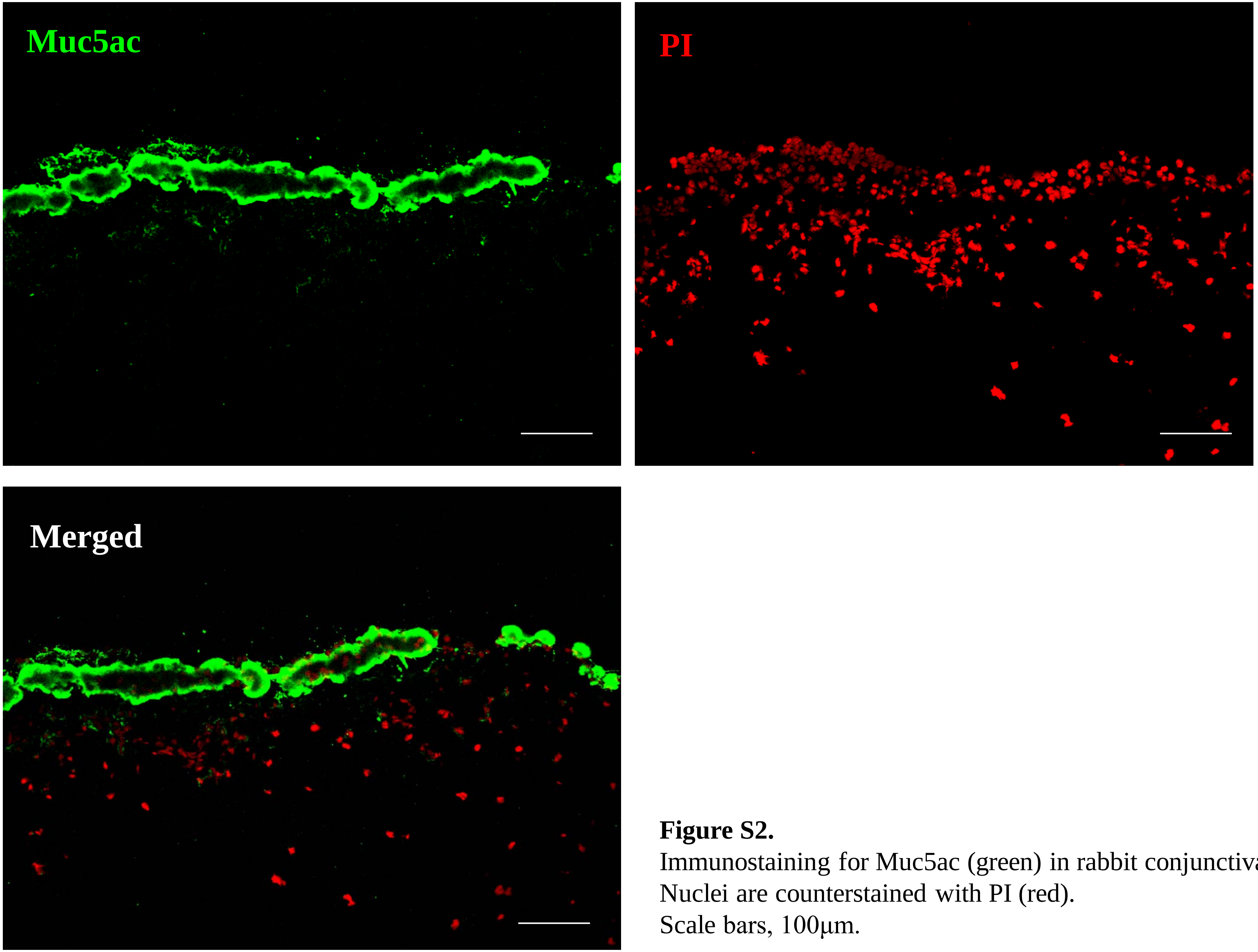

Figure S2.
Immunostaining for Muc5ac (green) in rabbit conjunctiva.
Nuclei are counterstained with PI (red).
Scale bars, 100µm.

Supplemental Table 1. Sequence for PCR

Muc1	F	GGAATGTTTCGGCACTGATT	
	R	GAACTCCAGACTGGGCAGAG	size 249
Muc4	F	GTGCCTTTCTCTGGAACAGC	
	R	CACCCTTGAGATGGGGTAGA	size 248
Muc16	F	TGCCAAATACCAGTGGAACA	
	R	GTCTCCGAGCCAAAGATGAG	size 176
Galectin3	F	ACTTTAACCCCCGCTTCAAT	
	R	TTCCCAGCTTGTTGATTTCC	size 234
Muc5ac	F	CCCCAACGTCAAGAACAACT	
	R	TCAAACAGGCAGTTCGAGTG	size 162

F:Forward, R: Reverse

Supplemental Information 1.

CLUSTAL 2.1 multiple sequence alignment

Muc1

```
human           CGCTCCACCTCTCAAGCAGCCAGCGCCTGCCTGAATCTGTTCTGCCCCCTCCCCACCCAT
rabbit          ---------------GCCACCAGCCCCG--------------------------------
                               **..***** **

human           TTCACCACCACCATGACACCGGGCACCCAGTCTCCTTTCTTCCTGCTGCTGCTCCTCACA
rabbit          ------TCCACGAAGTCACC----------------------------------------
                      :**** *:*:****

human           GTGCTTACAGTTGTTACGGGTTCTGGTCATGCAAGCTCTACCCCAGGTGGAGAAAAGGAG
rabbit          ------------------------------------TCTGCCCCGG--------------
                                                    ***.****.*

human           ACTTCGGCTACCCAGAGAAGTTCAGTGCCCAGCTCTACTGAGAAGAATGCTGTGAGTATG
rabbit          ------------------------------------------------------------


human           ACCAGCAGCGTACTCTCCAGCCACAGCCCCGGTTCAGGCTCCTCCACCACTCAGGGACAG
rabbit          -------------------CCACCAGCCCCACGTCGGTCTCAGCCACCAGCCCCGTCCAC
                                    *..*******.  **.* ***. ******  *. * .**

human           GATGTCACTCTGGCCCCGGCCACGGAACCAGCTTCAGGTTCAGCTGCCACCTGGGGACAG
rabbit          GAAGTCACCTCTGCCCCAGCCACCAGCCCTGCATCAGACTTGGCCACCAGCCCTGTCCAT
                **:*****    *****.***** ...**:**:****. * .** .*** *   * .**

human           GATGTCACCTCGGTCCCAGTCACCAGGCCAGCCCTGGG------CTCCACCACCCCGCCA
rabbit          GAAGTCACCTCTGCCCCTGCCACCAGCCCCACCCCCACGTCGGTCTCAGCCACCTCCCCC
                **:******** * ***:* ****** **..***  .       ***..***** * **.

human           GCCCACGATGTCACCTCAGCCCCGGACAACAAGCCAGCCCCGGGCTCCACCGCCCCCCCA
rabbit          GTTCACGAAGTCACCTCGGCCCCGGCCACCAGCCCCACGTCGGTCTCAGATACCAGCCCC
                *  *****:********.*******.**.**. **..*  *** ***... .**. ***.
```

```
human      GCCCACGGTGTCACCTCGGCCCCGGACACCAGGCCGGCCCCGGGCTCCACCGCCCCCCCA
rabbit     GTCCATGAAGTCACCTCGGCCCTGACCACCAGCCCCACGTCGGTCTCAGCCACCAGCCCC
           * *** *.:************* *..****** ** .*  *** ***..**.**.  ***.

human      GCCCATGGTGTCACCTCGGCCCCGGACAACAGGCCCGCCTTGGGCTCCACCGCCCCTCCA
rabbit     GTCCACGAAGTTACCTCTGCCCCGGCCACCAGCCCCACGTCGGTCTCAGCCACCAGCCCC
           * *** *.:** ***** *******.**.*** ***.* * ** ***..**.**.   **.

human      GTCCACAATGTCACCTCG------GCCTCAGGCTCTGCATCAGGCTCAGCTTCTACTCTG
rabbit     GTCCATGAAGTCACCTCGGCCCCGGCCACCAGCCCCACGTCGGTCTCAGCCACCTCCCCC
           ***** .*:*********      ***:*..** * .*.**.* ****** :* :* *

human      GTGCACAACGGCACCTCTGCCAGGGCTACCACAACCCCAG---CCAGCAAGAGCACTCCA
rabbit     GTCCACGAAGTCACCTCTGCCCTGGCCACCACCATGCCAGTTGGCACTCTGAGCACACCA
           ** ***.*.* **********.  *** *****.*   ****     **   .:******:***

human      TTCTCAATTCCCAGCCACCACTCTGATACTCCTACCACCCTTGCCAGCCATAGCACCAAG
rabbit     TCTTCGGTTCCCAG---CCACACTGGCACTCCCACCACCCTTCCCAGCCCCAGCCACAG-
           *  **..*******   ****:***.  ***** ********* ******.  ***..**.

human      ACTGATGCCAGTAGCACTCACCATAGCACGGTACCTCCTCTCACCTCCTCCAATCACAGC
rabbit     --------------------------CACGGGTTCTCCTGTCACCACTTCGACTCAGAGC
                                     ***** : ***** *****:* ** *.*** ***

human      ACTTCTCCCCAGTTGTCTACTGGGGTCTCTTTCTTTTTCCTGTCTTTTCACATTTCAAAC
rabbit     ACTGCTCCCCAGGTGTCTGCTGGTTTGTCTTTCTTCTTCCTGTCTTTTTACATCACCAAC
           *** ******** *****.****  * ******** ************ **** :*.***

human      CTCCAGTTTAATTCCTCTCTGGAAGATCCCAGCACCGACTACTACCAAGAGCTGCAGAGA
rabbit     CTGCAGTTCAATTCTTCCCTGGAGGATCCAAGCAGCAAGTACTATCAAGAGCTGCAGAGG
           ** ***** ***** ** *****.*****.**** *.* ***** **************.

human      GACATTTCTGAAATGTTTTTGCAGATTTATAAACAAGGGGGTTTTCTGGGCCTCTCCAAT
rabbit     AATGTTTCGGCACTGATTTCACAGATCTATGGACAGAAGAACTTCCTGGGCCTCTCTGGT
           .* .**** *.*.**:*** .***** ***..***...*..  ** *********** ..*
```

```
human      ATTAAGTTCAGGCCAGGATCTGTGGTGGTACAATTGACTCTGGCCTTCCGAGAAGGTACC
rabbit     ATCAGGTTCAGGCCAGGATCCGTGGTGGTGGACTTGATTCTGGCCTTCCAGGAGGGCAGC
           ** *.*************** ********.  *.**** ***********..**.** * *

human      ATCAATGTCCACGACGTGGAGACACAGTTCAATCAGTATAAAACGGAAGCAGCCTCTCGA
rabbit     ACCAATGAAGTCACGGTGCAGTCACAGTTTATTCAGAATATACCGCAAGCAGCCAG---G
           * *****:.  :*..  *** **:******* *:****:***:*.** ********:     .

human      TATAACCTGACGATCTCAGACGTCAGCGTGAGTGATGTGCCATTTCCTTTCTCTGCCCAG
rabbit     TATAACCTGAACATTTCAAGAGTCACAGTGCGTGATGTTCTGTCTCCCTCCTCTGCCCAG
           **********.  ** ***...**** .***.******* * .* *** * **********

human      TCTGGGGCTGGGGTGCCAGGCTGGGGCATCGCGCTGCTGGTGCTGGTCTGTGTTCTGGTT
rabbit     TCTGG------AGTTCCAGGCTGGGGGATCGCCCTGCTCGTGCTGGTCTGTGTCCTGCTT
           *****      .** *********** ***** ***** ************** *** **

human      GCGCTGGCCATTGTCTATCTCATTGCCTTGGCTGTCTGTCAGTGCCGCCGAAAGAACTAC
rabbit     GCGCTGGCCATCATCTATTTCATAGCCCTGGCCGTGTGTCAGTGCCGTCGCAAGAACTGC
           *********** .***** ****:*** **** ** *********** **.*******.*

human      GGGCAGCTGGACATCTTTCCAGCCCGGGATACCTACCATCCTATGAGCGAGTACCCCACC
rabbit     GGGCAGCTGGATCTCTTCCCGACCCGGGATACCTACCACCCCATGAGTGAGTACCCTACC
           *********** .**** **..**************** ** ***** ******** ***

human      TACCACACCCATGGGCGCTATGTGCCCCCTAGCAGTACCGATCGTAGCCCCTATGAGAAG
rabbit     TACCACACCCACGGCCGCTACGTGCCCCCTGGCAGCACCAGACGGAGCCCATATGAGGAG
           *********** ** ***** *********.**** ***..:** *****.******.**

human      GTTTCTGCAGGTAATGGTGGCAGCAGCCTCTCTTACACAAACCCAGCAGTGGCAGCCACT
rabbit     GAATCTGCGGGCAACGGGGGTAGCGGCCTTTCTTACACGAGTCCACCCGTGGCAGCCACT
           *::*****.** ** ** ** ***.**** ********.*.  *** *.************

human      TCTG-CCAACTTGTAGGGGCACGTCGCCCGCTGAGCTGAGTGGCCAGCCAGTGCCATTCC
rabbit     TCTGCCCATCTTGCTGGGACATG-----TACTTTTACCAAGGGCCAGCCCGGGGCTGGCT
           **** ***:**** :***.** *      .** :  .   *.  ********.* * *:  *
```

```
human           ACTCCACTCAGGTTCTTCAGGGCCAGAGCCCCTGCACCCTGTTTGGGCTGGTGAGCTGGG
rabbit          ------------------------------------------------------------


human           AGTTCAGGTGGGCTGCTCACAGCCTCCTTCAGAGGCCCCACCAATTTCTCGGACACTTCT
rabbit          ------------------------------------------------------------


human           CAGTGTGTGGAAGCTCATGTGGGCCCCTGAGGGCTCATGCCTGGGAAGTGTTGTGGTGGG
rabbit          ------------------------------------------------------------


human           GGCTCCCAGGAGGACTGGCCCAGAGAGCCCTGAGATAGCGGGGATCCTGAACTGGACTGA
rabbit          ------------------------------------------------------------


human           ATAAAACGTGGTCTCCCACTGCGCCAA
rabbit          ---------------------------
```

Underline fields are primer sequences we used.

Supplemental Information 2.

CLUSTAL 2.1 multiple sequence alignment

Muc4

```
human           CTCTTTTGTCCTCTTCCCAGGTTCCCTGGCCCCTTCGGAGAAACGCACTTGGTTCGGGCC
rabbit          ------------------------ACATCTCCCTCCAAATTTAGCCAGATG-------CC
                                        .*:   **** *..* ::*  ** :**       **

human           AGCCGCCTGAGGGGACGGGCTCACGTCTGCTCCTCACACTGCAGCTGCTGGGCCGTGGAG
rabbit          ATCAACAACATCCGAAGTGCTCACAACACCAACTTCTACATATTCAACTG----------
                * *..*.: *   **.* ******.:*: *:.** .  **: .: *:.***

human           CTTCCCCAGGGAGCCAGGGGGACTTTTGCCGCAGCCATGAAGGGGGCACGCTGGAGGAGG
rabbit          ------------------------------------------------------------


human           GTCCCCTGGGTGTCCCTGAGCTGCCTGTGTCTCTGCCTCCTTCCGCATGTGGTCCCAGGA
rabbit          -------------------------------------------------------TAGAA
                                                                        **.*

human           ACCACAGAGGACACATTAATAACTGGAAGTAAAACTGCTGCCCCAGTCACCTCAACAGGC
rabbit          ACAACAGAAGAAACATCATCACCCAAAACTGGGAGTCCTCCTCCAGTCACCTCAGGAGTC
                **.*****.**.**** *: *.* ..** *...* * ** * ************.  ** *

human           TCAACAACAGCGACACTAGAGGGACAATCAACTGCAGCTTCTTCAAGGACCTCTAATCAG
rabbit          TCCACAACAAC------------------------AACTTCTTCAACAACCTCCACTCAG
                **.******.*                        *.********* .***** *.****

human           GACATATCAGCTTCATCTCAGAACCACCAGACTAAGAGCACGGAGACCACCAGCAAAGCT
rabbit          GAAACATCAGCAGTTTCTCAGAATCCACAGACTCAGGGTAGGGAGACCACCAGAGGATCT
                **.* ******:   :******** *..******.**.* * ************...* **

human           CAAACCGACACCCTCACGCAGATGATGACATCAACTCTTTTTTCTTCCCCAAGTGTACAC
rabbit          CCAACCAGCACCCATGTGGAGGTGACTACATCAACACCTCCATCCTATCCAGGTGAAAAC
                *.****..*****: .  * **.***  ********:* *  :** *.  ***.***:*.**
```

```
human          AATGTGATGGAGACAG------------CTCCTCCAGATGAAATGACCACATCATTTCCC
rabbit         ACTCTGACAGAGACTGTCTCTCAGGCGACATCCCCTCCAGGTGAAACAGCCACATCTCCC
               *.* *** .*****:*            *: * **: .:*.:.:.**..*.:*** ****

human          TCCAGTGTCACCAACACACTCATGATGACATCAAAGACTATAACAATGACAACCTCCACA
rabbit         TCCAGTGTTAGCCAGACATCAAC---AACATCAGAAGTGCTCAAAACTCCAACCTCCTCA
               ******** * *.* ***  .*    .******.*..  .*.*.**  .********:**

human          GACTCCACTCTTGGAAACACAGAAGAGACATCAACAGCAGGAACTGAAAGTTCTACCCCA
rabbit         TATTCAACTGTGGAAACAACCGAAGAAACGTCAACATCTAAAACTGGGAGTCCTCCTCCA
                * **.*** * *.**..**.*****.**.****** *:..*****..*** **.* ***

human          GTGACCTCAGCAGTCTCAATAACAGCTGGACAGGAAGGACAATCACGAACAACTTCCTGG
rabbit         GTCACCTCAGCAGTCTCCTCAACAG------------------------CAACTTCTTCA
               ** **************.: *****                        ******* * .

human          AGGACCTCTATCCAAGACACATCAGCTTCTTCTCAGAACCACTGGACTCGGAGCACGCAG
rabbit         ACAACCTCCACTCAGGAAACATCAGCCGTTTCTCAGAATCCACAGACTCAGGGTGGGGAG
               * .***** *  **.**.********   ********* *.. .*****.*.* . * **

human          ACCACCAGGGAATCTCAAACCAGCACCCTAACACACAGAACCACTTCAACTCCTTCTTTC
rabbit         ACCACCAGAGGATCTCCAACCAGCACCCATGTGGAGGTGACTACATCAGCACCTCCATCC
               ********.*.*****.***********::. . * . .** **:***.*:*** *:* *

human          TCTCCAAGTGTACACAATGTGACAGGGACTGTTTCTCAGAAGACATCTCCTTCAGGTGAA
rabbit         TCCCCAGGTAGACATAGTGTGTCACTGACAATTTCTCAGGCAACATCCCCTCTAGGTGAA
               ** ***.**. *** *.****:**  ***:.********...***** ***  *******

human          ACAGCTACCTCATCCCTCTGTAGTGTCACAAACACATCCATGATGACATCAGAGAAGATA
rabbit         ACAGCCACCACATCTGTTTCCATAGTCAGCAAGACACCCCTGAGAACAGCAGGAGTGATG
               ***** ***:****  * *  * :**** .** *** **.*** .*** ***...:***.

human          ACAGTGACAACCTCCACAGGCTCCACTCTTGGAAACCCAGGGGAGACATCATCAGTACCT
rabbit         ACAATGACAACTTCTGCACCCTCAACTCTGGCAACCACAGAAGG---TACGTCATCCTCT
               ***.******* ** .**  ***.***** * **.*.***..*.   ::*.***  . **
```

```
human     GTTACTGGAAGTCTTATGCCAGTCACCTCAGCAGCCTTAGTAACATTTGATCCAGAAGGA
rabbit    AACACTGGGAGTCCTCCTCCAGTCACCTCAGCAGTCTCCACAACAGCTGCAACAGAAGAA
          .: *****.**** *.  **************** **  .. ****  **.:.******.*

human     CAATCACCAGCAACTTTCTCAAGGACTTCTACTCAG---GACACAACAGCTTTTTCTAAG
rabbit    AAATCTGCATCAACCTCTTTAACGACCTCCACTCTGGATGGAACATTAGCATTTTCATGG
          .****: ** **** *  * ** *** ** ****:*   *..***: ***:*****::.*

human     AACCACCAGACTCAGAGCGTGGAGACCACCAGAGTATCTCAAATCAACACCCTCAACACC
rabbit    AACCCCCAGACTCAGAGCATGGGTACCAGCACAGCATCCCACACCAG---------TGCC
          ****.*************.***. **** ** ** *** **.* **.          .**

human     CTCACACCGGTTACAACATCAACTGTTTTATCCTCACCAAGTGGATTCAACCCAAGTGGA
rabbit    CCTACAGAGGAGACTGCATCAACTCCTACATCTTCTCTGAGTGGACACACTCTGACAGAG
          *  *** .**: **:.********  *: *** **:* .****** :**. * .* :*..

human     ACAGTTTCTCAGGAGACATTCCCTTCTGGTGAAACAACCACCTCATCCCCTTCCAGTGTC
rabbit    ACCATTTCCCAGGAGACAATCCCTCCAACTAAAATGGCCACTATACCTCCTTCCAGTACC
          **..**** *********:***** *:. *.*** ..**** : * * *********. *

human     AGCAATACATTCCTGGTAACATCAAAGGTGTTCAGAATGCCAACCTCCAGAGACTCTACT
rabbit    CACAACACAGACCCAACAACAATGGAGGTATCCAAAATGCCAACCTCCCCAGACTCCACT
          ..*** *** :** .. ****: ..****.* **.*************. ****** ***

human     CTTGGAAACACAGAGGAGACATCACTATCTGTAAGTGGAACCATTTCTGCAATCACTTCC
rabbit    GTGGAAAACAGAGGGCACACATCACTACCTACAACTGGGAACATGCCTGTATTCACTTCT
           * *.***** **.* * ********* **. ** ***.*.***  *** *:*******

human     AAAGTTTCAACCATATGGTGGTCAGACACTCTGTCAACAGCACTCTCCCCCAGTTCTCTA
rabbit    AAAGTGTCAACCACAC------------------------CCCTCTCAACTAGTTCTTTA
          ***** ******* *                         *.*****..* ****** **

human     CCTCCAAAAATATCCACAGCTTTCCACACCCAGCAGAGTGAAGGTGCAGAGACCACAGGA
rabbit    ACTTCAAAAACATTAACAGTTTTCCCCACCTACCAAAGAAAAGGCACAGAGACTCCAAGA
          .** ****** ** .**** *****.**** * **.**:.**** .******* .**.**
```

```
human           CGGCCTCATGAGAGGAGCTCATTCTCTCCAGGTGTGTCTCAAGAAATATTTACTCTACAT
rabbit          TGGTCTCATGACAGCAGCCTGATTTCTACAAGTATGTCCCAGGAGACATTCATTACAGAG
                 ** ******* ** ***  .:* ***.**.**.**** **.**.* *** * *. * *

human           GAAACAACAACATGGCCTTCCTCATTCTCCAGCAAAGGCCACACAACTTGGTCACAAACA
rabbit          AAAATGACAATGGTGACATCATCATTCTCTGTTGATAACAACACAACTCAGCTTCCAAAA
                .*** .**** .  *.*:**.******** .  .*:..*.******** .*  :*.**.*

human           GAACTGCCCTCAACATCAACAGGTGCTGCCACTAGGCTTGTCACAGGAAATCCATCTACA
rabbit          GAACTATTGTCAATGCCAACAGGAGTTACCTGTTACCTTGTCACCAAAAGCCCAACTCCT
                *****.   **** . *******:* *.**: *:. ********...**. ***:**.*:

human           GGGACAGCTGGCACTATTCCAAGGGTCCCCTCTAAGGTCTCAGCAATAGGGGAACCAGGA
rabbit          TCAACAACTGGCACCACAGTTTGGGTCACACCTGAGGTCCCAACAACAGGAGAATTAGGA
                  .***.******* * :  ::*****.*. **.***** **.*** ***.***  ****

human           GAGCCCACCACATACTCCTCCCACAGCACAACTCTCCCAAAAACAACAGGGGCAGGCGCC
rabbit          GAGTCAACCACACAGCCTGATATGAGCCCATCTACTCAGAAAACCATGATAGCAGTTTCC
                *** *.****** *  *  . .: ***.**:**.  *..*****.* .. .****   **

human           CAGACACAATGGACACAAGAAACGGGGACCACTGGAGAGGCTCTTCTCAGCAGCCCAAGC
rabbit          CAGACACAATGGACACAAGGTCCAAGGACCACTGGAGAGATTCACATCAACAGTCCGAGC
                *******************.:.*..**************. **: .***.*** **.***

human           TACAGTGTGACTCAGATGATAAAAACGGCCACATCCCCATCTTCTTCACCTATGCTGGAT
rabbit          TCTGGTTCAACTATGGTCATGGAAACAGTGGCATCCCCATCTCCTTCATCAACAATAAGC
                *. .**  .***.:*.* **..****.*  .*********** ***** *:* ..*...

human           AGACACACATCCCAACAAATTACAACGGCACCATCAACAAATCATTCAACAATACATTCC
rabbit          AGACATACATCTCAAGAAACTATCACAGCATGGCCAAAAGCTCAGTCCACACCACTCCCA
                ***** ***** *** *** ** .**.***  . ***.*..*** **.***. **:  *.

human           ACAAGCACCTCTCCTCAGGAATCACCAGCTGTTTCCCAAAGGGGTCACACTCAAGCCCCG
rabbit          CCCAGCAGCTTTCCTCGGGAATCCTTGTCTATGCCCAGCATAGGTCACACTCACGCCACA
                .*.**** ** *****.******.  . **.*  **...* .***********.***.*.
```

```
human           CAGACCACACAAGAATCACAAACCACGAGGTCCGTCTCCCCCATGACTGACACCAAGACA
rabbit          CTGACCACACCAGAATCCCAAGCCATGAGTTCAATCACCCCCGAGACTGACACACCCAAT
                *:********.******.***.*** *** **..**:*****.:*********... *.:

human           GT---CACCACCCCAGGTTCTTCCTTCACAGCCAGTGGGCACTCGCCCTCAGAAATTGTT
rabbit          GTGGCAACATCCGCAGCCTCTTCTGCCACACCAAGTGGACCCTCATCCTCAGAAAGCACT
                **   .**.:** ***  *****   **** *.*****.*.***. *********  . *

human           CCTCAGGACGCACCCACCATAAGTGCAGCAACAACCTTTGCCCCAGCTCCCACCGGGGAT
rabbit          CCTCAGGAGGCATCCACCGTGGCTGCGGTGACAACCTCCATCCCTGCACCCTCCAGG---
                ******** *** *****.*.. ***.* .*******  . ***:**:***:**.**

human           GGTCACACAACCCAGGCCCCGACCACAGCACTGCAGGCAGCACCCAGCAGCCATGATGCC
rabbit          ---------GACAGCCACCCAGCCACAGGACCACGGCCAGCACCACCCAGCCCCAGCCCC
                         ..*..  .***..****** ** .*.* *******.. *****. ..  **

human           ACCCTGGGGCCCTCAGGAGGCACGTCACTTTCCAAAACAGGTGCCCTTACTCTGGCCAAC
rabbit          ACCCTCCGGCCCTCCGGAGCCCCATCACTCTCCACAGCAAGCACGGTTTCCCAGGACGCT
                *****  *******.**** *.*.***** ****.*.**.* .*  **:* *:**.*..

human           TCTGTAGTGTCAACACCAGGGGGCCCAGAAGGACAATGGACATCAGCCTCTGCCAGCACC
rabbit          GCTGTCACCTCCCCATCAGGGGGCCCAGGAGGACAGCAGGCATTGCCTTTTGCAAGCACC
                 ****..  **..** ************.******. .*.*** . * * ***.******

human           TCACCTGACACAGCAGCAGCCATGACCCATACCCACCAGGCTGAGAGCACAGAGGCCTCT
rabbit          TCCCCTGACACAGCCCAAGTCATTTCCCAAACCCAGCAGACTCGAGGCACAGAGACCACC
                **.***********. .** *** :****:***** ***.** ...********.**:*

human           GGACAAACACAGACCAGCGAACCGGCCTCCTCAGGGTCACGAACCACCTCAGCGGGCACA
rabbit          GGGGAGGCTCAGCCCAGCACACCAGCCCCCTCAGTGCCAGACCCCACCGCCGCAGCCATG
                **. *..*:***.*****..***.*** ****** * ** ...***** *.**.* ** .

human           GCTACCCCTTCCTCATCCGGGGCGAGTGGCACAACACCTTCAGG---AAGCGAAGGAATA
rabbit          GCTGCCACGTCTTCCCCCACGGCAAGGGGCACAACCTTTGGAAGGGCTTTCCCGGAAACG
                ***.**.* ** **. **. ***.** ********.  *  *.*   :: * ..*.** .
```

```
human      TCCACCTCAGGAGAGACGACAAGGTTTTCATCAAACCCCTCCAGGGACAGTCACACAACC
rabbit     GCCACTGCAGCCAAGACCACCGACTTTCCCACAACTCCCTCCAGCAGCAGCCACGCAGCT
            ****  *** ..**** **... *** *.:***. ******** ..*** ***.**.*

human      CAGTCAACAACCGAATTGCTGTCCGCCTCAGCCAGTCATGGTGCCATCCCAGTAAGCACA
rabbit     CCGCCAACGGCTGAGCTGTTGTCCTCTGGCACCAGTCCTGACGCTACCCCTGCAGCCGTA
           *.* ****..* **. ** ***** *   ..******.**. ** * ***:* *. *. *

human      GGAATGGCGTCTTCGATCGTCCCCGGCACCTTTCATCCCACCCTCTCTGAGGCCTCCACT
rabbit     GGAATGAGGTCTCCTGCTGTACCCAGCACCTCTCCTCCTACCATCACTGGGGTCCCGACT
           ******. **** * .  **.***.******* **.*** ***.**:***.** * * ***

human      GCAGGGAGACCGACAGGACAGTCAAGCCCAACTTCTCCCAGTGCCTCTCCTCAGGAGACA
rabbit     GATTGGACAGCGTCTTCCAG------------------------GACCCCTCAGGAGAAG
           *.: *** * **:*:  ...                         :* **********..

human      GCCGCCATTTCCCGGATGGCCCAGACTCAGAGGACAAGAACCAGCAGAGGGTCTGACACT
rabbit     CCAGCTGTCTCCCAGATGGCTCACACTCCAAGTGCAGGAGCCACCAGAGGACCTGAAGTC
            *.** .* ****.****** ** ****..** .**.**.*** ******. ****..

human      ATCAGCCTGGCGTCCCAGGCAACCGACACCTTCTCAACAGTCCCACCCACACCTCCATCG
rabbit     AGCACCTTGGCCTGGCAGCTCACTGACACCTTCTCAGCAGTCACAGCGCCACCACCCTCG
           * ** * **** *  ***  .** ************.*****.** * .****:**.***

human      ATCACATCCACTGGGCTTACATCTCCACAAACCGAGACCCACACTCTGTCACCTTCAGGG
rabbit     CCCACAACCACG------------------------------------------------
           . ****:****

human      TCTGGTAAAACCTTCACCACGGCCCTCATCAGCAACGCCACCCCTCTTCCTGTCACCTAC
rabbit     ------------------------------------------------------------

human      GCTTCCTCGGCATCCACAGGTCACACCACCCCTCTTCATGTCACCGATGCTTCCTCAGTA
rabbit     ------------------------------------------------------------
```

```
human           TCCACAGGTCACGCCACCCCTCTTCCTGTCACCAGCCCTTCCTCAGTATCCACAGGTCAC
rabbit          ------------------------------------------------------------

human           ACCACCCCTCTTCCTGTCACCGACACTTCCTCAGAATCCACAGGTCACGTCACCCCTCTT
rabbit          ------------------------------------------------------------

human           CCTGTCACCAGCTTTTCCTCAGCATCCACAGGTGACAGCACCCCTCTTCCTGTCACTGAC
rabbit          ------------------------------------------------------------

human           ACTTCCTCAGCATCCACAGGTCACGTCACCCCTCTTCCTGTCACCAGCCTTTCCTCAGCA
rabbit          ------------------------------------------------------------

human           TCCACAGGTGACACCACCCCTCTTCCTGTCACTGACACTTCCTCAGCATCCACAGGTCAC
rabbit          ---CCCACTGAGCACACATCACAAACCGAGACTCGCACTCCCTCACCTTCAGGCAGCAGC
                   .*.. *** ..***. *:*::.* *: *** .**** ***** *:**.. ..* ..*

human           GCCACCTCTCTTCCTGTCACCGACACTTCCTCAGTATCCACAGGTCACACCACCCCTCTT
rabbit          ACAGCCTCTATTAATTCTTCATTGTCTCAGTCTGTACCTGGCCCTCCCAGCACCGGGAGT
                .*..*****.**..*   :*. : :** . **:*** * . .  **.** ****   . *

human           CCTGTCACCGACACTTCCTCAGCATCCACAGGTCACGCCACCTCTCTTCCTGTCACCGAC
rabbit          CCT---------------------------------------------------------
                ***

human           ACTTCCTCAGTATCCACAGGTCACACCACCCCTCTTCATGTCACTGATGCTTCCTCAGCA
rabbit          ---CCTACCCTAGTTCCAGGTCACACTGCTCCCCTGCCAGTTACAACTACTTCCTCCCCA
                    * :*. **   .********** .* ** ** *.:** **:..*.*******. **

human           TCCACAGGTCAGGCCACCCCTCTTCCTGTCACCAGCCTTTCCTCAGTATCCACAGGTGAC
rabbit          CCCACAG-----------------------------------------------------
                 ******
```

```
human      ACCACGCCTCTTCCTGTCACTAGCCCTTCCTCAGCATCCACAGGTCACGCCACCCCTCTT
rabbit     ------------------------------------------------------------

human      CTTGTCACCGACACTTCCTCAGCATCCACAGGACACGCCACCCCTCTTCCTGTCACCGAC
rabbit     ------------------------------------------------------------

human      GCTTCCTCAGTGTCCACAGATCACGCCACCTCTCTTCCTGTAACCATCCCTTCCGCAGCA
rabbit     ------------------------------------------------------------

human      TCCACAGGTCACACCACCCCTCTTCCTGTCACCGACACTTCCTCAGCATCCACAGGTCAG
rabbit     ------------------------------------------------------------

human      GCCACCTCTCTTCTTGTCACCGACACTTCCTCAGTATCCACAGGTGACACCACGCCTCTT
rabbit     ------------------------------------------------------------

human      CCTGTCACTAGCACTTCCTCAGCATCCACAGGTCACGTCACTCCTCTTCATGTCACCAGC
rabbit     ------------------------------------------------------------

human      CCTTCCTCAGCATCCACAGGTCACGCCACCCCTCTTCCTGTCACCAGCCTTTCCTCAGCA
rabbit     ------------------------------------------------------------

human      TCCACAGGTGACACCATGCCTCTTCCTGTCACTAGCCCTTCCTCAGCATCCACAGGTGAC
rabbit     ------------------------------------------------------------

human      ACCACCCCTCTTCCTGTCACCGACGCTTCCTCAGTATCCACAGGTCACACCACCCCTCTT
rabbit     ------------------------------------------------------------
```

```
human           CATGTCACTGATGCTTCCTCAGCATCCACAGGTCAGGCCACCCCTCTTCCTGTCACCAGC
rabbit          ------------------------------------------------------------

human           CTTTCCTCAGTATCCACAGGTGACACCACGCCTCTTCCTGTCACTAGCCCTTCCTCAGCA
rabbit          ------------------------------------------------------------

human           TCCACAGGTCACGCCACCCCTCTTCTTGTCACCGACACTTCCTCAGCATCCACAGGACAC
rabbit          ------------------------------------------------------------

human           GCCACCCCTCTTCCTGTCACCGACGCTTCCTCAGTGTCCACAGATCACGCCACCTCTCTT
rabbit          ------------------------------------------------------------

human           CCTGTAACCATCCCTTCCGCAGCATCCACAGGTCACACCACCCCTCTTCCTGTCACCGAC
rabbit          ------------------------------------------------------------

human           ACTTCCTCAGCATCCACAGGTCAGGCCACCTCTCTTCTTGTCACCGACACTTCCTCAGTA
rabbit          ------------------------------------------------------------

human           TCCACAGGTGACACCACGCCTCTTCCTGTCACTAGCACTTCCTCAGCATCCACAGGTCAC
rabbit          ------------------------------------------------------------

human           GTCACTCCTCTTCATGTCACCAGCCCTTCCTCAGCATCCACAGGTCACGCCACCCCTCTT
rabbit          ------------------------------------------------------------

human           CCTGTCACCAGCCTTTCCTCAGCATCCACAGGTGACACCATGCCTCTTCCTGTCACTAGC
rabbit          ------------------------------------------------------------
```

```
human           CCTTCCTCAGCATCCACAGGTGACACCACCCCTCTTCCTGTCACCGACGCTTCCTCAGTA
rabbit          ------------------------------------------------------------

human           TCCACAGGTCACACCACCCCTCTTCCTGTCACCAGCCCTTCCTCAGCATCTACAGGTCAC
rabbit          ------------------------------------------------------------

human           ACCACCCCTCTTCCTGTCACCGACACTTCCTCAGCATCCAAAGGTGACACCACCCCTCTT
rabbit          ------------------------------------------------------------

human           CCTGTCACCAGCCCTTCCTCAGCATCTACAGGTCACACCACCCCTCTTCCTGTCACCGAC
rabbit          ------------------------------------------------------------

human           ACTTCCTCAGCATCCACAGGTGACACCACCCCTCTTCCTGTCACCAATGCTTCCTCATTA
rabbit          ------------------------------------------------------------

human           TCCACAGGTCACGCCACCCCTCTTCATGTCACCAGCCCTTCCTCAGCATCCACAGGTCAC
rabbit          ------------------------------------------------------------

human           GCCACCCCTCTTCCTGTCACCAGCACTTCCTCAGCATCCACCGGTCACGCCACCCCTCTT
rabbit          ------------------------------------------------------------

human           CCTGTCACCGGCCTTTCCTCAGCTACCACAGATGACACCACCCGTCTTCCTGTCACCGAC
rabbit          ------------------------------------------------------------

human           GTTTCCTCGGCATCCACAGGTCAGGCCACCCCTCTTCCTGTCACCAGCCTTTCCTCAGTA
rabbit          ------------------------------------------------------------
```

```
human      TCCACAGGTGACACCACGCCTCTTCCTGTCACTAGCCCTTCCTCAGCATCCACAGGTCAC
rabbit     ------------------------------------------------------------

human      GCCAGCCCTCTTCTTGTCACTGACGCTTCCTCAGCATCCACAGGTCAGGCCACCCCTCTT
rabbit     ------------------------------------------------------------

human      CCTGTCACCGACACTTCCTCAGTATCCACAGCTCACGCCACCCCACTTCCTGTCACCGGC
rabbit     ------------------------------------------------------------

human      CTTTCTTCAGCTTCCACAGATGACACCACCCGTCTTCCTGTCACCGACGTTTCCTCGGCA
rabbit     ------------------------------------------------------------

human      TCCACAGGTCAGGCCATCCCTCTTCCTGTCACCAGCCCTTCCTCAGCATCCACAGGTGAC
rabbit     ------------------------------------------------------------

human      ACCACCCCTCTTCCTGTCACCGACGCTTCCTCAGCATCCACAGGTGACACCACCTCTCTT
rabbit     ------------------------------------------------------------

human      CCTGTCACCATCCCTTCCTCAGCATCTTCAGGTCACACCACCTCTCTTCCTGTCACCGAC
rabbit     ------------------------------------------------------------

human      GCTTCCTCAGTGTCCACAGGTCACGCCACCTCTCTTCTTGTCACCGACGCTTCCTCAGTA
rabbit     ------------------------------------------------------------

human      TCCACAGGTGACACCACCCCTCTTCCTGTCACCGACACTAACTCAGCATCCACAGGTGAC
rabbit     ------------------------------------------------------------
```

```
human     ACCACCCCTCTTCATGTCACCGACGCTTCCTCAGTATCCACAGGTCACGCCACCTCTCTT
rabbit    ------------------------------------------------------------

human     CCTGTCACCAGCCTTTCCTCAGCATCCACAGGTGACACCACGCCTCTTCCTGTCACTAGC
rabbit    ------------------------------------------------------------

human     CCTTCCTCAGCATCCTCAGGTCACACCACCCCTCTTCCTGTCACCGACGCTTCCTCAGTA
rabbit    ------------------------------------------------------------

human     CCCACAGGTCACGCCACCTCTCTTCCTGTCACCGACGCTTCCTCAGTGTCCACAGGTCAC
rabbit    ------------------------------------------------------------

human     GCCACCCCTCTTCCTGTCACCGACGCTTCCTCAGTGTCCACAGGTCATGCCACCCCTCTT
rabbit    ------------------------------------------------------------

human     CCGGTCACCGACACTTCCTCAGTATCTACAGGACAGGCCACCCCTCTTCCTGTCACCAGC
rabbit    ------------------------------------------------------------

human     CTTTCCTCAGCATCCACTGGTGACACCACGCCGCTTCCTGTCACCGATACTTCCTCAGCA
rabbit    ------------------------------------------------------------

human     TCCACAGGTCAGGACACCCCTCTTCCTGTCACCAGCCTTTCCTCAGTATCCACAGGTGAC
rabbit    ------------------------------------------------------------

human     ACCACGCCTCTTCCTGTCACTAACCCTTCCTCAGCATCCACAGGTCACGCCACCCCTCTT
rabbit    ------------------------------------------------------------
```

human CTTGTCACCGACGCTTCCTCAATATCCACAGGTCACGCCACCTCTCTTCTTGTCACCGAC
rabbit --

human GCTTCCTCAGTATCCACAGGTCACGCCACCGCTCTTCATGACACCGATGCTTCCTCATTA
rabbit --

human TCCACAGGGGACACCACCCCTCTTCCTGTCACCAGCCCTTCCTCAACATCCACAGGTGAC
rabbit --

human ACCACCCCTCTTCCTGTCACCGAAACTTCCTCAGTATCCACAGGTCACGCCACCTCTCTT
rabbit --

human CCTGTCACCGACACTTCCTCAGCATCCACAGGTCACGCCACCTCTCTTCCTGTCACCGAC
rabbit --

human ACTTCCTCAGCATCCACAGGTCACGCCACCCCTCTTCCTGTCACCGACACTTCCTCAGCA
rabbit --

human TCCACAGGTCAGGCCACCCCTCTTCCTGTCACCAGCCCTTCCTCAGCATCCACAGGTCAC
rabbit --

human GCCATCCCTCTTCTTGTCACCGACACTTCCTCAGCATCCACAGGACAGGCCACCCCTCTT
rabbit --

human CCTGTCACCAGCCTTTCCTCAGCATCCACAGGTGACACCACCCCTCTTCCTGTCACCGAC
rabbit --

```
human      GCTTCCTCAGTGTCCACAGGTCACGCCACCTCTCTTCCTGTCACCAGCCTTTCCTCAGTA
rabbit     ------------------------------------------------------------

human      TCCACAGGTGACACCACTCCTCTTCCTGTCACTAGCCCTTCCTCAGCATCCACAGGTCAC
rabbit     ------------------------------------------------------------

human      GCCACCCCTCTTCATGTCACCGACGCTTCCTCAGCATCCACAGGTCACGCCACCCCTCTT
rabbit     ------------------------------------------------------------

human      CCTGTCACCAGCCTTTCCTCAGCATCCACAGGTGACACCACGCCTCTTCCTGTCACTAGC
rabbit     ------------------------------------------------------------

human      CCTTCCTCAGCATCCACAGGTCACGCCACCCCTCTTCATGTCACCGACGCTTCCTCAGTA
rabbit     ------------------------------------------------------------

human      TCCACAGGTGACACCACCCCTCTTCCTGTCACCAGCTCTTCCTCAGCATCCTCAGGTCAC
rabbit     ------------------------------------------------------------

human      ACCACCCCTCTTCCTGTCACCGACGCTTCCTCAGCATCCACAGGTGACACCACCCCTCTT
rabbit     ------------------------------------------------------------

human      CCTGTCACCGACACTTCCTCAGCATCCACAGGTCACGCCACCCATCTTCCTGTCACCGGC
rabbit     ------------------------------------------------------------

human      CTTTCCTCAGCTTCCACAGGTGACACCACCCGTCTTCCTGTCACCAACGTTTCCTCGGCA
rabbit     ------------------------------------------------------------
```

```
human     TCCACAGGTCATGCCACCCCTCTTCCTGTCACCAGCACTTCCTCAGCATCCACAGGTGAC
rabbit    ------------------------------------------------------------

human     ACCACCCCTCTTCCTGGCACCGACACTTCCTCAGTATCCACAGGTCACACCACCCCTCTT
rabbit    ------------------------------------------------------------

human     CTTGTCACCGACGCTTCGTCAGTATCCACAGGTGACACCACCCGTCTTCCTGTCACCAGC
rabbit    ------------------------------------------------------------

human     CCTTCCTCAGCATCTACAGGTCACACCACCCCTCTACCTGTCACCGACACTCCCTCAGCA
rabbit    ------------------------------------------------------------

human     TCCACAGGTGACACCACCCCTCTTCCTGTCACCAATGCTTCCTCATTATCCACACGTCAC
rabbit    ------------------------------------------------------------

human     GCCACCTCTCTTCATGTCACCAGCCCTTCCTCAGCATCCACAGGTCACGCCACCTCTCTT
rabbit    ------------------------------------------------------------

human     CCTGTCACCGACACTTCCGCAGCATCCACAGGTCACGCCACCCCTCTTCCTGTCACCAGC
rabbit    ------------------------------------------------------------

human     ACTTCCTCAGCATCCACAGGTGACACCACCCCTCTTCCTGTCACCGACACTTACTCAGCA
rabbit    ------------------------------------------------------------

human     TCCACAGGTCAGGCCACCCCTCTTCCTGTCACCAGCCTTTCCTCAGTATCCACAGGTGAC
rabbit    ------------------------------------------------------------
```

```
human     ACCACGCCTCTTCCTGTCACTAGCCCTTCCTCAGCATCCACAGGTCACGCCACTCCTCTT
rabbit    ------------------------------------------------------------

human     CTTGTCACCGACGCTTCCTCAGCATCCACAGGTCAGGCCACCCCTCTTCCTGTCACCAGC
rabbit    ------------------------------------------------------------

human     CTTTCCTCAGTATCCACAGGTGACACCACGCCTCTTCCTGTCACTAGCCCTTCCTCAGCA
rabbit    ------------------------------------------------------------

human     TCCACCGGTCATGCCACCTCTCTTCCTGTCACCGACACTTCCTCAGCATCCACAGGTGAC
rabbit    ------------------------------------------------------------

human     ACCACCTCTCTTCCTGTCACCGACACTTCCTCAGCATACACAGGTGACACCACCTCTCTT
rabbit    ------------------------------------------------------------

human     CCTGTCACCGACACTTCCTCATCATCCACAGGTGACACCACCCCTCTTCTTGTCACCGAG
rabbit    ------------------------------------------------------------

human     ACTTCCTCAGTATCCACAGGTGACACCACCCCTCTTCCTGTCACCGACACTTCCTCAGCA
rabbit    ------------------------------------------------------------

human     TCCACAGGTCACGCCACCCCTCTTCCTGTCACCAACACTTCCTCAGTATCCACAGGTCAC
rabbit    ------------------------------------------------------------

human     GCCACCCCTCTTCATGTCACCAGCCCTTCCTCAGCATCCACAGGTCACACCACCCCTCTT
rabbit    ------------------------------------------------------------
```

```
human     CCTGTCACCGACGCTTCGTCAGTGTCCACAGGTCACGCCACCTCTCTTCCTGTCACCGAC
rabbit    ------------------------------------------------------------

human     GCTTCCTCAGTGTTCACAGGTCATGCCACCTCTCTTCCTGTCACCATCCCTTCCTCAGCA
rabbit    ------------------------------------------------------------

human     TCCTCAGGTCACACCACCCCTCTTCCTGTCACCGACGCTTCCTCAGTGTCCACAGGTCAC
rabbit    ------------------------------------------------------------

human     GCCACCTCTCTTCCTGTCACCGACGCTTCCTCAGTGTCCACAGGTCATGCCACCCCTCTT
rabbit    ------------------------------------------------------------

human     CCTGTCACCGACGCTTCCTCAGTGTCCACAGGTCACGCTACCCCTCTTCCTCTCACCAGC
rabbit    ------------------------------------------------------------

human     CTTTCCTCAGTATCCACAGGTGACACCACGCCTCTTCCTGTCACCGACACTTCCTCAGCA
rabbit    ------------------------------------------------------------

human     TCCACAGGTCAGGCCACCCCTCTTCCTGTCACCAGCCTTTCCTCAGTATCCACAGGTGAC
rabbit    ------------------------------------------------------------

human     ACCACCCCTCTTCCTGTCACCGACACTTCCTCAGCATCCACAGGTCACGCCACCTCTCTT
rabbit    ------------------------------------------------------------

human     CCTGTCACCGACACTTCCTCAGCATCCACAGGTCACGCCACCCCTCTTCCTGACACCGAC
rabbit    ------------------------------------------------------------
```

```
human      ACTTCCTCAGCATCCACAGGTCACGCCACCCTTCTTCCTGTCACCGACACTTCCTCAGCA
rabbit     ------------------------------------------------------------

human      TCCATAGGTCACGCCACCTCTCTTCCTGTCACCGACACTTCCTCAATATCCACAGGTCAC
rabbit     ------------------------------------------------------------

human      GCCACCCCTCTTCATGTCACCAGCCCTTCCTCAGCATCCACCGGTCACGCCACCCCGCTT
rabbit     ------------------------------------------------------------

human      CCTGTCACCGACACTTCCTCAGCATCCACAGGTCACGCCAACCCTCTTCATGTCACCAGC
rabbit     ------------------------------------------------------------

human      CCTTCCTCAGCATCCACCGGTCACGCCACCCCGCTTCCTGTCACCGACACTTCCTCAGCA
rabbit     ------------------------------------------------------------

human      TCCACAGGTCACGCCACCCCTCTTCCTGTCACCAGCCTTTCCTCAGTATCCACAGGTGAC
rabbit     ------------------------------------------------------------

human      ACCACGCCTCTTCCTGTCACTAGCCCTTCCTCAGCATCCACAGGTCACACCACCCCTCTT
rabbit     ------------------------------------------------------------

human      CCTGTCACCGACACTTCCTCAGCATCCACAGGTCAGGCCACCGCTCTTCCTGTCACCAGC
rabbit     ------------------------------------------------------------

human      ACTTCCTCAGCATCCACAGGTGACACCACCCCTCTTCCTGTCACCGACACTTCCTCAGCA
rabbit     ------------------------------------------------------------
```

```
human       TCCACAGGTCAGGCCACCCCTCTTCCTGTCACCAGCCTTTCCTCAGTATCCACAGGTGAC
rabbit      ------------------------------------------------------------

human       ACCACGCCTCTTCCTGTCACTAGCCCTTCCTCAGCATCCACAGGTCACGCCACTCCTCTT
rabbit      ------------------------------------------------------------

human       CTTGTCACCGACGCTTCCTCAGCATCCACAGGTCAGGCCACCCCTCTTCCTGTCACCAGC
rabbit      ------------------------------------------------------------

human       CTTTCCTCAGTATCCACAGGTGACACCACGCCTCTTCCTGTCACTAGCCCTTCCTCAGCA
rabbit      ------------------------------------------------------------

human       TCCACCGGTCATGCCACCTCTCTTCCTGTCACCGACACTTCCTCAGCATCCACAGGTGAC
rabbit      ------------------------------------------------------------

human       ACCACCTCTCTTCCTGTCACCGACACTTCCTCAGCATACACAGGTGACACCACCTCTCTT
rabbit      ------------------------------------------------------------

human       CCTGTCACCGACACTTCCTCATCATCCACAGGTGACACCACCCCTCTTCTTGTCACCGAG
rabbit      ------------------------------------------------------------

human       ACTTCCTCAGTATCCACAGGTCACGCCACTCCTCTTCTTGTCACCGACGCTTCCTCAGCA
rabbit      ------------------------------------------------------------

human       TCCACAGGTCACGCCACCCCTCTTCATGTCACCAGCCCTTCCTCAGCATCCACAGGTGAC
rabbit      ------------------------------------------------------------
```

```
human     ACCACCCCTGTGCCTGTCACCGACACTTCCTCAGTATCCACAGGTCACGCCACCCCTCTT
rabbit    ------------------------------------------------------------

human     CCTGTCACCGGCCTTTCCTCAGCTTCCACAGGTGACACCACCCGTCTTCCTGTCACCGAC
rabbit    ------------------------------------------------------------

human     ATTTCCTCGGCATCCACAGGTCAGGCCACCCCTCTTCCTGTCACCAACACTTCCTCAGTA
rabbit    ------------------------------------------------------------

human     TCCACAGGTGACACCATGCCTCTTCCTGTCACTAGCCCTTCCTCAGCATCCACAGGTCAC
rabbit    ------------------------------------------------------------

human     GCCACCCCTCTTCCTGTCACCAGCACTTCCTCAGCATCCACCGGTCACGCCACCCCTGTT
rabbit    ------------------------------------------------------------

human     CCTGTCACCAGCACTTCCTCAGCATCTACAGGTCACACCACCCCTCTTCCTGTCACCGAC
rabbit    ------------------------------------------------------------

human     ACTTCCTCAGCATCCACAGGTGACACCACCCCTCTTCCTGTCACCAGCCCTTCCTCAGCA
rabbit    ------------------------------------------------------------

human     TCTACAGGTCACACCACCCCTCTTCATGTCACCATCCCTTCCTCAGCATCCACAGGTGAC
rabbit    ------------------------------------------------------------

human     ACCAGCACTCTTCCTGTCACCGGCGCTTCCTCAGCATCCACCGGTCACGCCACCCCTCTT
rabbit    ------------------------------------------------------------
```

```
human    CCTGTCACCGACACTTCCTCAGTATCCACCGGTCACGCCACGCCTCTTCCTGTCACCAGC
rabbit   ------------------------------------------------------------

human    CTTTCCTCAGTATCCACAGGTGACACCACCCCTCTTCCTGTCACCGACGCTTCCTCGGCA
rabbit   ------------------------------------------------------------

human    TCCACAGGTCAGGCCACCCCTCTTCCTGTCACCAGCCTTTCCTCAGTATCCACAGGTGAC
rabbit   ------------------------------------------------------------

human    ACCACCCCTCTTCTTGTCACCGACGCTTCCTCAGTATCCACAGGTCACGCCACCCCTCTT
rabbit   ------------------------------------------------------------

human    CCTGTCACCGACACTTCCTCAGCATCCACAGGTGACACCACCCGTCTTCCTGTCACGGAC
rabbit   ------------------------------------------------------------

human    ACTTCCTCAGCATCCACAGGTCAGGCCACCCCTCTTCCTGTCACCAGCCTTTCCTCAGTA
rabbit   ------------------------------------------------------------

human    TCCACAGGTGACACCACCCCTCTTCTTGTCACCGACGCTTCCTCAGTATCCACAGGTCAC
rabbit   ------------------------------------------------------------

human    GCCACCCCTCTTCCTGTCACCGACACTTCCTCAGCATCCACAGGTGACACCACCCGTCTT
rabbit   ------------------------------------------------------------

human    CCTGTCACGGACACTTCCTCAGCATCCACAGGTCAGGCCACCCCTCTTCCTGTCACCATC
rabbit   ------------------------------------------------------------
```

```
human           CCTTCCTCATCATCCTCAGGTCACACCACCCCTCTTCCTGTCACCAGCACTTCCTCAGTA
rabbit          ------------------------------------------------------------

human           TCTACAGGTCACGTCACCCCTCTTCATGTCACCAGCCCTTCCTCAGCATCCACAGGTCAC
rabbit          ------------------------------------------------------------

human           GTCACCCCTCTTCCTGTCACCAGCACTTCCTCAGCATCCACAGGTCACGCCACCCCTCTT
rabbit          ------------------------------------------------------------

human           CTTGTCACCGACGCTTCCTCAGTGTCCACAGGTCACGCCACGCCTCTTCCTGTCACCGAC
rabbit          ------------------------------------------------------------

human           GCTTCCTCAGCATCCACAGGTGACACCACCCCTCTTCCTGTCACCGACACTTCCTCAGCA
rabbit          ------------------------------------------------------------

human           TCCACAGGTCAGGCCACCCCTCTTCCTGTCACCAGCCTTTCCTCAGTATCCACAGGTGAC
rabbit          ------------------------------------------------------------

human           ACCACCCCTCTTCCTGTCACCGACGCTTCCTCAGCATCCACAGGTCACGCCACCCCTCTT
rabbit          ------------------------------------------------------------

human           CCTGTCACCATCCCTTCCTCAGTATCCACAGGTGACACCATGCCTCTTCCTGTCACTAGC
rabbit          ------------------------------------------------------------

human           CCTTCCTCAGCATCCACAGGTCACGCCACCCCTCTTCCTGTTACCGGCCTTTCCTCAGCT
rabbit          ------------------------------------------------------------
```

```
human           TCCACAGGTGACACCACCCCTCTTCCTGTCACCGACACTTCCTCAGCATCCACACGTCAC
rabbit          ------------------------------------------------------------

human           GCCACCCCTCTTCCTGTCACCGACACTTCCTCAGCTTCCACAGATGACACCACCCGTCTT
rabbit          ------------------------------------------------------------

human           CCTGTCACCGACGTTTCCTCGGCATCCACAGGACATGCCACCCCTCTTCCTGTCACCAGC
rabbit          ------------------------------------------------------------

human           ACTTCCTCAGCATCCACAGGTGACACCACCCCTCTTCCTGTCACCGACACTTCCTCAGTA
rabbit          ------------------------------------------------------------

human           TCCACAGGTCACGCCACCTCTCTTCCTGTCACCAGCCGTTCCTCAGCATCCACAGGTCAC
rabbit          ------------------------------------------------------------

human           GCCACCCCCCTTCCTGTCACCGACACTTCCTCAGTATCCACAGGTCACGCCACCCCTCTT
rabbit          ------------------------------------------------------------

human           CCTGTCACCAGCACTTCCTCAGTATCTACAGGTCACGCCACCCCTCTTCCTGTCACCAGC
rabbit          ------------------------------------------------------------

human           CCTTCCTCAGCATCCACAGGTCACGCCACCCCTGTTCCTGTCACCAGCACTTCCTCAGCA
rabbit          ------------------------------------------------------------

human           TCCACAGGTGACACCACCCCTCTTCCTGTCACCAATGCTTCCTCATTATCCACAGGTCAC
rabbit          ------------------------------------------------------------
```

```
human       GCCACCCCTCTTCATGTCACCAGCCCTTCCTCAGCATCCAGAGGTGACACCAGCACTCTT
rabbit      ------------------------------------------------------------

human       CCTGTCACCGATGCTTCCTCAGCATCCACCGGTCACGCCACCCCTCTTCCTCTCACCAGC
rabbit      ------------------------------------------------------------

human       CTTTCCTCAGTATCCACAGGTGACACCACGCCTCTTCCTGTCACCGACACTTCCTCTGCA
rabbit      ------------------------------------------------------------

human       TCCACAGGTCAGGCCACCCCTCTTCCTGTCACCAGCCTTTCCTCAGTATCCACAGGTGAC
rabbit      ------------------------------------------------------------

human       ACCACGCCTCTTCCTGTCACCATCCCTTCCTCAGCATCCTCAGGTCACACCACCTCTCTT
rabbit      ------------------------------------------------------------

human       CCTGTCACCGACGCTTCCTCAGTGTCCACAGGTCACGGCACCCCTCTTCCTGTCACCAGC
rabbit      ------------------------------------------------------------

human       ACTTCCTCAGCATCCACAGGTGACACCACCCCTCTTCCTGTCACCGACACTTCCTCAGCA
rabbit      ------------------------------------------------------------

human       TCCACAGGTCACGCCACCCCTCTTCCTGTCACCGACACTTCCTCAGCATCCACAGGTCAC
rabbit      ------------------------------------------------------------

human       GCCACCCCTCTTCCTGTCACCAGCCTTTCCTCAGTATCCACAGGTCACGCCACCCCTCTT
rabbit      ------------------------------------------------------------
```

```
human           GCTGTCAGCAGTGCTACCTCAGCTTCCACAGTATCCTCGGACTCCCCTCTGAAGATGGAA
rabbit          -------------------------------CATCTTTGGGCTCCACAACGAAGACCGAA
                                                *** * **.****.*:. *****  ***

human           ACACCAGGAATGACAACACCGTCACTGAAGACAGACGGTGGGAGACGCACAGCCACATCA
rabbit          ACATCAGGAATGATCCCAACCTCACTGTGGATAAATGGTGGGAGAAGCACAGCACTGTCA
                *** ********* ..**.* ******:.** *.* *********.*******..  .***

human           CCACCCCCCACAACCTCCCAGACCATCATTTCCACCATTCCCAGCACTGCCATGCACACC
rabbit          CCGCCCTCAACAACCTCCCTGACCCCTACGAACTCCACGCCCACCACATCCACAGGCTTC
                **.*** *.**********:****.  *  :.*:***  **** ***: *** . .*: *

human           CGCTCCACAGCTGCCCCCATCCCCATCCTGCCTG---------AGAGAGGAGTTTCCCTC
rabbit          CGTTCAACAACTCCCTCTGTCCCCGTCCAGCAGGGACAAGCGGGCCCAGGCGTCTCCCTC
                ** **.***.** ** * .*****.***:**. *         . .  ***.** ******

human           TTCCCCTATGGGGCAGGCGCCGGGGACCTGGAGTTCGTCAGGAGGACCGTGGACTTCACC
rabbit          TTTCCCTACGGGTCCAGCGTTGGAGACACGCCGTTCGTCAGGAGGACAGTGGACTTCACC
                ** ***** *** *..***  **.***.  * .**************.************

human           TCCCCACTCTTCAAGCCGGCGACTGGCTTCCCCCTTGGCTCCTCTCTCCGTGATTCCCTC
rabbit          TCTCAGCTCTTCAAGCCCCGCATTGGCTTCCCCCTGGGCTCCTTTCTGCGAGATTCCCTC
                ** *..***********    * ************ ******* *** **:*********

human           TACTTCACAGACAATGGCCAGATCATCTTCCCAGAGTCAGACTACCAGATTTTCTCCTAC
rabbit          TACTTCACAGACAACGGCCAGATCATCTTCCCGGAGTCAGACAGCCAGATCTTCTCCTAC
                ************** *****************.*********:.****** *********

human           CCCAACCCACTCCCAACAGGCTTCACAGGCCGGGACCCTGTGGCCCTGGTGGCTCCGTTC
rabbit          CCCAATCCGCCTCGGCGAGGCTTCACAGCTTGGGACCCCGTGGCCGTGGTGGCTCCCTTC
                ***** **.*  * .. ***********   ******* ****** ********** ***

human           TGGGACGATGCTGACTTCTCCACTGGTCGGGGGACCACATTTTATCAGGAATACGAGACG
rabbit          TGGGACGATGCTGACTTCTCCATCCGTCGGGGAACCATATTTTACCAGGAATATGAGACA
                **********************   *******.**** ****** ******** *****.
```

```
human       TTCTATGGTGAACACAGCCTGCTAGTCCAGCAGGCCGAGTCTTGGATTAGAAAGATGACA
rabbit      TTCTATAATGAACACAACCCGCTAGTCTGGCAGGTGGAGTCTTGGATTCGCCCAGTGGAC
            ******..********.** ******* .*****  ************.*.....**...

human       AACAACGGGGGCTACAAGGCCAGGTGGGCCCTAAAGGTCACGTGGGTCAATGCCCACGCC
rabbit      CTTTGGGGTGAGTGGACCCATAGCAGCTCCCCTGGAGTGACCTGG----GAATCCCAGCA
            .: :. ** *. *. *.  . ** :*  *** :...** ** ***    .:. **..**.

human       TATCCTGCCCAGTGGACCCTCGGGAG-------CAACACCTACCAAGCCATCCTCTCCAC
rabbit      TTTGAAGCCAGGTGGACCAGGAACAGGTGATACCTGCACCTACCAGGCCATCCTCTCCAC
            *:* .:***..*******.  .. **       *:.*********.**************

human       GGACGGGAGCAGGTCCTATGCCCTGTTTCTCTACCAGAGCGGTGGGATGCAGTGGGACGT
rabbit      CGACGGGAGCACGTCCTACGCACTGTTTCTTTACCAGAGCAATGGGATGCAGTGGGATGT
             ********** ****** **.******** *********..*************** **

human       GGCCCAGCGCTCAGGCAACCCGGTGCTCATGGGCTTCTCTAGTGGAGATGGCTATTTCGA
rabbit      AGCCCAGCGCCCAGGCAACTCGGTCCTCATGGGCTTCTCCAGTGCAGACGGGTATTTTGC
            .********* ******** **** ************** **** *** ** ***** *.

human       AAACAGCCCACTGATGTCCCAGCCAGTGTGGGAGAGGTATCGCCCTGATAGATTCCTGAA
rabbit      CAACAGCCCGCTGATTTCCCGGCCAGTGTGGGAGAGATATCGTCCGGACCGATGGCTGAA
            .********.***** ****.***************.***** ** ** .***  *****

human       TTCCAACTCAGGCCTCCAAGGGCTGCAGTTCTACAGGCTACACCGGGAAGAAAGGCCCAA
rabbit      TTCCAAACTAGGCCTCCGGGGGCTGCAGATCTACAGGCTCCACAGGGAGGCGATACCCAA
            ******.  ********..*********:**********.***.****.*..* .*****

human       CTACCGTCTCGAGTGCCTGCAGTGGCTGAAGAGCCAGCCTCGGTGGCCCAGCTGGGGCTG
rabbit      CTACTATCTCAAGTGCCAGCA---------------------------------------
            **** .****.******:***

human       GAACCAGGTCTCCTGCCCTTGTTCCTGGCAGCAGGGACGACGGGACTTACGATTCCAACC
rabbit      ---------------CCCTTGCACCTGGCAGCAGGGACGATGGGACTTACGATTCCGGCC
                           ****** :***************** ***************..**
```

```
human           CGTCAGCATAGGTCGCTGGGGCCTCG---GCAGTAGGCAGCTGTGCAGCTTCACCTCTTG
rabbit          TGTGGGCTCAGGCTGGTGGGGCCATGGCGGCGGCCGGAAGCTGTGCCGCTTCTCCTCCTG
                 ** .**: ***  * *******: *   **.* .**.********.*****:**** **

human           GCGAGGAGGCGTGTGCTGCAGCTACGGGCCCTGGGGAGAGTTTCGTGAAGGCTGGCACGT
rabbit          GCAAGGAGGCGTGTGCTGCAGCTACGGGCCCTGGGGAGAGCTTCGCGAAGGCTGGGGAGT
                **.************************************* **** ********* ..**

human           GCAGCGTCCTTGGCAGTTGGCCCAGGAACTGGAGCCACAGAGCTGGTGCTGCCGCTGGAA
rabbit          GGACAGTCCTTGGCACTTTGAGCAGGAACTGGAGGCGCAGAACTGGTGCTGCCGCTGGAA
                * * .********** ** *. ************ *.****.******************

human           TGACAAGCCCTACCTCTGTGCCCTGTACCAGCAGAGGCGGCCCCACGTGGGCTGTGCTAC
rabbit          CGACAAGCCCTCCTTCTGCTCGCTGTACAAGCGGAGGCGGCCGCCCGTCAGCTGTGCCGG
                 **********.* ****  * ******.***.********* *.*** .******* . 

human           ATACAGGCCCCCACAGCCCGCCTGGATGTTCGGGGACCCCCACATCACCACCTTGGATGG
rabbit          ATACAGGCCCTCCAGGCCGGGTTGGATGTTTGGGGACCCCCACATCACCACCTTGGATGG
                ********** *...*** *  ******** *****************************

human           TGTCAGTTACACCTTCAATGGGCTGGGGGACTTCCTGCTGGTCGGGGCCCAAGACGGGAA
rabbit          CGCCAATTTCACCTTCAATGGTCTGGGGGACTTCCTGCTGGTCCGGGCCTGGGACGGGAA
                 * **.**:************ ********************* ***** ..********

human           CTCCTCCTTCCTGCTTCAGGGCCGCACCGCCCAGACTGGCTCAGCCCAGGCCACCAACTT
rabbit          CTCCTCCTTCCTGCTGCAAGGCCGCACTGCCCAGACCGGCTCCGCCCAGGCCACCAACTT
                *************** **.******** ******** *****.*****************

human           CATCGCCTTTGCGGCTCAGTACCGCTCCAGCAGCCTGGGCCCCGTCACGGTCCAATGGCT
rabbit          CATTGCCTTTGCAGCTCAGTATGAGTCCAGCAGCCTGGCCCCCATCACGGTTCAATGGTT
                *** ********.********  . ************* ****.******* ****** *

human           CCTTGAGCCTCACGACGCAATCCGTGTCCTGCTGGATAACCAGACTGTGACATTTCAGCC
rabbit          CCTGGAGCCCAATGACACAATCCGTGTGCTGCACAATAACCAGACTGTGACATTTGAAAC
                *** ***** .* ***.********** ****: .******************** *..*
```

```
human      TGACCATGAAGACGGCGGAGGCCAGGAGACGTTCAACGCCACCGGAGTCCTCCTGAGCCG
rabbit     CGACCTTGAAGATGCAGAAGGCCAGGAGATTTTCAACACCACCGGGGTGCTGCTGACCCG
            ****:****** * .*.***********  ******.*******.** ** **** ***

human      CAACGGCTCTGAGGTCTCGGCCAGCTTCGACGGCTGGGCCACCGTCTCGGTGATCGCGCT
rabbit     CAACGGCTCTCAGGTCTCAGCCAGCTTTGATGGCACGGTGGCCATCTCGGTGATGGCTGT
           ********** *******.******** ** ***: **  .**.********** **  *

human      CTCCAACATCCTCCACGCCTCCGCCAGCCTCCCGCCCGAGTACCAGAACCGCACGGAGGG
rabbit     CTCCCACCTCCTCCACGCCTCCTCCAGCCTCCCAGAGGAGTACCAGGGCCGCACGGAGGG
           ****.**.************** **********. . *********..************

human      GCTCCTGGGGGTCTGGAATAACAATCCAGAGGACGACTTCAGGATGCCCAATGGCTCCAC
rabbit     CCTCCTGGGGCTCTGGAACAACAATCCCGATGATGACTTCCGGATGCCCAATGGCACTTC
            ********* ******* ********.** ** ******.**************:* :*

human      CATTCCCCCAGGGAGCCCTGAGGAGATGCTTTTCCACTTTGGAATGACCTGGCAGATCAA
rabbit     CGTGGCCCGCGGGAGCTCCGAGGAGCAGCTTTTCCACTATGGGATGACCTGGAAGCTCAA
           *.*  *** .****** * ******.:***********:***.*********.**.****

human      CGGGACAGGCCTCCTTGGCAAGAGGAATGACCAGCTGCCTTCCAACTTCACCCCTGTTTT
rabbit     CGGGACAGGCCTCCTTGGCCAGAGGGACGACCAGCTGCCTCCCACCTTCACCCCTGTCTT
           *******************.*****.* ************ ***.************ **

human      CTACTCACAACTGCAAAAAAACAGCTCCTGGGCTGAACATTTGATCTCCAACTGTGACGG
rabbit     CTTCTCCCAACTGCTGGACAACAGCTCTGGGGACCCGGCTCTGGTCTCTGGGTGCAGTGG
           **:***.*******:..*.********  ***.  .. .* **.**** .. ** .. **

human      AGATAGCTCATGCATCTATGACACCCTGGCCCTGCGCAACGCAAGCATCGGACTTCACAC
rabbit     AGACAGACAATGCATCTATGACGCCCTGGCCATGCGAGACGCCGGTGCAGGCATCCACAC
           *** **. .*************.********.****..****..* . .**..* *****

human      GAGGGAAGTCAGTAAAAACTACGAGCAGGCGAACGCCACCCTCAATCAGTACCCGCCCTC
rabbit     CAGGACACTGTTTAGAACGTACCGGCAGATGAACGCCACCCAGAATCAGTTCCCGCCCTC
            ***..* * : **.**. *** .****. ***********: *******:*********
```

```
human          CATCAATGGTGGTCGTGTGATTGAAGCCTACAAGGGGCAGACCACGCTGATTCAGTACAC
rabbit         CATCAAGGGCGAGCATGTGATTTGCGCCTATAAGGGGAAGAGCGTGTGGATTCCGTACAC
               ****** ** *. *.******* ..***** ******.*** *. *  *****.******

human          CAGCAATGCTGAGGATGCCAACTTCACGCTCAGAGACAGCTGCACCGACTTGGAGCTCTT
rabbit         CAGCGACTCGGAGAATGTCGTGTTCACGCTCAGAAACAACTGCACTGACATCAAGCTCTT
               ****.*  * ***.*** *.: ************.***.****** ***:* .*******

human          TGAGAATGGGACGTTGCTGTGGACACCCAAGTCGCTGGAGCCATTCACTCTGGAGATTCT
rabbit         CGAGAATGGCACATTGCTGTGGACCCCGAAGTCACTGGAACCGTTCACTCTGGAGATCCT
                ******** **.***********.** *****.*****.**.************** **

human          AGCAAGAAGTGCCAAGATTGGCTTGGCATCTGCACTCCAGCCCAGGACTGTGGTCTGCCA
rabbit         AGTGAGAAGCACCAAGACGGGCCTCTCGTCTGCCTTCCAGCCCCAGACCGTGCTCTGCGC
               ** .***** .******  *** *  *.*****. ********..*** *** ***** .

human          TTGCAATGCAGAGAGCCAGTGTTTGTACAATCAGACCAGCAGGGTGGGCAACTCCTCCCT
rabbit         ATGCAGTGCAGAGAGCCAGTGTCTGTACAACCAGACCAGCTGGGTGGGCAATTCCTCCCT
               :****.**************** ******* *********:********** ********

human          GGAGGTGGCTGGCTGCAAGTGTGACGGGGGCACCTTCGGCCGCTACTGCGAGGGCTCCGA
rabbit         CCAGGTGGCTGACTGCAAGTGTGATGGGGACACCTTTGGCCGCTTCTGTGAGCGCTCCAA
                 *********.************ ****.****** *******:*** *** *****.*

human          GGATGCCTGTGAGGAGCCGTGCTTCCCGAGTGTCCACTGCGTTCCTGGGAAGGGCTGCGA
rabbit         GGACCCCTGTGAGGAGGAGTGCTTCCCTGGCGTGAGCTGCGTTCCTGAGCGAGGCTGCGA
               ***  *********** .********* .* ** ..***********.*...********

human          GGCCTGCCCTCCAAACCTGACTGGGGATGGGCGGCACTGTGCGGCTCTGGGGAGCTCTTT
rabbit         GGCCTGCCCCATAGACATGACCGGAGACGGGCGGCACTGTGCAGCCCTGGAGAACTCCGA
               ********* . *.**.**** **.** **************.** ****.**.***  :

human          CCTGTGTCAGAACCAGTCCTGCCCTGTGAATTACTGCTACAATCAAGGCCACTGCTACAT
rabbit         CCTCTGTCAGAACCACTCTTGCCCTGTGAATTACTGCCACAACCAGGGCCACTGCTACAT
               *** *********** ** ****************** **** **.**************
```

```
human          CTCCCAGACTCTGGGCTGTCAGCCCATGTGCACCTGCCCCCCAGCCTTCACTGACAGCCG
rabbit         TGCCCCGACGCTGAGCTGCCAGCCCTCCTGCACCTGCCCCCCAGCCTTCACTGACGCCCG
                 ***.*** ***.**** ******:  ***************************. ***

human          CTGCTTCCTGGCTGGGAACAACTTCAGTCCAACTGTCAACCTAGAACTTCCCTTAAGAGT
rabbit         CTGCTTCCTGGCTGGGAATAGCTTCACTCCAACCACCCATCAAGAGCTTCCCTTAAGAAC
               ****************** *.***** ****** . *.* *:***.************.

human          CATCCAGCTCTTGCTCAGTGAAGAGGAAAATGCCTCCATGGCAGAAGTCAACGCCTCGGT
rabbit         CATCCAGCTCTTGCTCATCGAAGATGAAAATGCCTCCGTAGCAGAGGTCAACGCCTCGGT
               *****************  ***** ************.*.*****.**************

human          GGCATACAGACTGGGGACCCTGGACATGCGGGCCTTTCTCCGCAACAGCCAAGTGGAACG
rabbit         GGCCTACAGGCTGGGGACCCTGGACGTGCGTGCCTTTCTCTGGAACAGCCTAGTGGAACC
               ***.*****.***************.**** ********* * *******:********

human          AATCGATTCTGCAGCACCGGCCTCGGGAAGCCCCATCCAACACTGGATGGTCATCTCGGA
rabbit         AATCGCCTCTCCGGCCCCCGGCTCGGGAAGCCTCATTCAGCGCTGGAAGGTCACCTCGCA
               *****. *** *.**.** * *********** *** **.*.*****:***** **** *

human          GTTCCAGTACCGCCCTCGGGGCCCGGTCATTGACTTCCTGAACAACCAGCTGCTGGCCGC
rabbit         TTTCCAGTACCGGCCTCAGGGCCCTGTCATCGACTTCCTCAACAACCGGCTTCAGGAGGC
                *********** ****.****** ***** ******** *******.*** *:**. **

human          GGTGGTGGAGGCGTTCTTATACCACGTTCCACGGAGGAG------------TGAGGAGCC
rabbit         AGTGGTGCAGGCCTTCTTCCTGCAGGCTCAGAGGAGGAGGTGGAAGAGGAGCGAGGGGCC
               .****** **** *****. : ** * **...*******             ****.***

human          CAGGAACGACGTGGTCTTCCAGCCCATCTCCGGGGAAGACGTGCGCGATGTGACAGCCCT
rabbit         CAGGAACAACGTGCTCTTCTACCCCATCTCAAGGGTGGACGTGCATGATCTCGCAGCGCT
               *******.***** ***** * ********..***:.*******. *** * .**** **

human          GAACGTGAGCACGCTGAAGGCTTACTTCAGATGCGATGGCTACAAGGGCTACGACCTGGT
rabbit         GAATGTGAGCACGCTGGAGACATACCTCAAATGCAATGGCTACGAGGGCTACCACCTGAT
               *** ************.**.*:*** ***.****.********.******** *****.*
```

```
human       CTACAGCCCCCAGAGCGGCTTCACCTGCGTGTCCCCGTGCAGTAGGGGCTACTGTGACCA
rabbit      CTACGGCCCCCAGAGCGGCTTCACCTGCACGTCCCCGTGCACCGAGGGCTACTGTGACCA
            ****.***********************. ***********  ..***************

human       TGGAGGCCAGTGCCAGCACCTGCCCAGTGGGCCCCGCTGCAGCTGTGTGTCCTTCTCCAT
rabbit      CG-AGGCCAGTGCCAGCACTGCCTGA----------------------------------
             * ****************   *  *

human       CTACACGGCCTGGGGCGAGCACTGTGAGCACCTGAGCATGAAACTCGACGCGTTCTTCGG
rabbit      ------------------------------------------------------------

human       CATCTTCTTTGGGGCCCTGGGCGGCCTCTTGCTGCTGGGGGTCGGGACGTTCGTGGTCCT
rabbit      ------------------------------------------------------------

human       GCGCTTCTGGGGTTGCTCCGGGGCCAGGTTCTCCTATTTCCTGAACTCAGCTGAGGCCTT
rabbit      ------------------------------------------------------------

human       GCCTTGAAGGGGCAGCTGTGGCCTAGGCTACCTCAAGACTCACCTCATCCTTACCGCACA
rabbit      ------------------------------------------------------------

human       TTTAAGGCGCCATTGCTTTTGGGAGACTGGAAAAGGGAAGGTGACTGAAGGCTGTCAGGA
rabbit      ------------------------------------------------------------

human       TTCTTCAAGGAGAATGAATACTGGGAATCAAGACAAGACTATACCTTATCCATAGGCGCA
rabbit      ------------------------------------------------------------

human       GGTGCACAGGGGGAGGCCATAAAGATCAAACATGCATGGATGGGTCCTCACGCAGACACA
rabbit      ------------------------------------------------------------
```

```
human           CCCACAGAAGGACACTAGCCTGTGCACGCGCGCGTGCACACACACACACACACACACGAG
rabbit          ------------------------------------------------------------


human           TTCATAATGTGGTGATGGCCCTAAGTTAAGCAAAATGCTTCTGCACACAAAACTCTCTGG
rabbit          ------------------------------------------------------------


human           TTTACTTCAAATTAACTCTATTTAAATAAAGTCTCTCTGACTTTTTGTGTCTCCAAAAAA
rabbit          ------------------------------------------------------------


human           AAAAAAAAAAA
rabbit          -----------
```

Underline fields are primer sequences we used.

Supplemental Information 3.

CLUSTAL 2.1 multiple sequence alignment

Muc5ac

```
human           GGGCACTCTTCCCCGCCGTCCACACAATGAGTGTTGGCCGGAGGAAGCTGGCCCTGCTCT
rabbit          ------------------------------------------------------------


human           GGGCCCTGGCTCTCGCTCTGGCCTGCACCCGGCACACAGGCCATGCCCAGGATGGCTCCT
rabbit          ------------------------------------------------------------


human           CCGAATCCAGCTACAAGCACCACCCTGCCCTCTCTCCTATCGCCCGGGGGCCCAGCGGGG
rabbit          ------------------------------------------------------------


human           TCCCGCTCCGTGGGGCGACTGTCTTCCCATCTCTGAGGACCATCCCTGTGGTACGAGCCT
rabbit          ----------------------CCTCCAATGGCCGCGG----TTGCTTCGGCCACAGCCC
                                      * *** **  * * **    *  **  **    ****

human           CCAACCCGGCGCACAACGGGCGGGTGTGCAGCACCTGGGGCAGCTTCCACTACAAGACCT
rabbit          CGGACCCCGTGCACAACGGCCGGGTGTGCAGCACCTGGGGCGACTTCCACTACAAGACCT
                *  **** * ********* *********************  *****************

human           TCGACGGCGACGTCTTCCGCTTCCCCGGCCTCTGCAACTACGTGTTCTCCGAGCACTGCG
rabbit          TCGACGGCGCCGTCTTCCGCTTCCCGGGCCTCTGCAACTACGTGTTCTCTGCGCACTGCG
                ********* *************** *********************** * ********

human           GTGCCGCCTACGAGGATTTTAACATCCAGCTACGCCGCAGCCAGGAGTCAGCGGCCCCCA
rabbit          GCGGCGCCTACGAGGACTTCAACGTGCAGCTGCGGCGCAGCCGGCAGGACGGCGCTGTGG
                * * ************ ** *** * ***** ** ******* * **   *  **

human           CGCTGAGCAGGGTCCTCATGAAGGTGGATGGCGTGGTCATCCAGCTGACCAAGGGCTCCG
rabbit          CCCCGAGCAGGGTCATCATGAAGCTGGAGGGCCTGGTGGTCGAGCTCACCAGTCACTCCG
                * * ********** ******** **** *** ****  ** **** ****    *****
```

```
human           TCCTGGTCAACGGCCACCCGGTCCTGCTGCCCTTCAGCCAGTCTGGGGTCCTCATTCAGC
rabbit          TCCTGGTCAGCGGCCGCCCGGTGCAGCTGCCCTTCAGCCAGGCGGGGGTTCTCATAGAGC
                ********* ***** ****** * **************** * ***** *****  ***

human           AGAGCAGCAGCTACACCAAGGTGGAGGCCAGGCTGGGCCTTGTCCTCATGTGGAACCACG
rabbit          GCAGCAGCAGCTCCCTGAAGGTGGTGGCCAAGCTGGGCCTGGTCTTCATGTGGAACCAGG
                  ********** *   ******* ***** ********* *** ************* *

human           ATGACAGCCTGCTGCTGGAGCTGGACACCAAATACGCCAACAAGACCTGTGGGCTCTGTG
rabbit          ACGACAGCCTCCTGCTGGAGCTGGACTCCAAGTTCGCCAACCAGACCTGCGGGCTCTGTG
                * ******** *************** **** * ******* ******* **********

human           GGGACTTCAACGGGATGCCCGTGGTCAGCGAGCTCCTCTCCCACAACACCAAGCTGACAC
rabbit          GAGACTTCAACGGCATCCCCGCGTCCAGCGAGTTCCTGTCCCACAACACCAGGCTGACCC
                * *********** ** **** *  ******* **** ************* ****** *

human           CCATGGAATTCGGGAACCTGCAGAAGATGGACGACCCCACGGAGCAGTGTCAGGACCCTG
rabbit          CCGTGGAGTTCGGGAACCTGCAGAAGCTGGATGGCCCCATGGAGCAGTGCCAGGACCCGG
                ** **** ****************** **** * ***** ********* ******** *

human           TCCCTGAACCCCCGAGGAACTGCTCCACTGGCTTTGGCATCTGTGAGGAGCTCCTGCACG
rabbit          CCCCAGAAAACAAGAGGAACTGCTCCAC-AGCCCCGGT---------GAGCCCCTGCGTG
                 *** ***  *  *************** **   **          **** *****  *

human           GCCAG--CTGTTCTCTGGCTGCGTGGCCCTGGTGGACGTCGGCAGCTACCTGGAGGCTTG
rabbit          GAGGGGGCGGGGCGGGGGTTGCGGGGACCCTACG-----CGG--GGTGCCCCGAG-CTTG
                *   *  * *  *   ** **** ** **    *     ***  * * **  *** ****

human           CAGGCAAGACCTCTGCTTCTGTGAAGACACCGACCTGCTCAGCTGCGTCTGCCACACCCT
rabbit          GACTCCAGAGC---GCTCCCGGGGC----CCGACCAGNCGACCT----CTGCCGT-----
                 *  * *** *   *** * * *      ****** *   * **    *****

human           TGCCGAGTACTCCCGGCAGTGCACCCATGCAGGGGGGT--TGCCCCAGGACTGGCGGGGC
rabbit          ----GAGTGCCCCCCGCCCGGCCCCAACCCGGCAGGCTGGCGCGCCCACGCCGGCGCAGC
                    **** * *** **   ** ** *  * *  ** *   ** **    * ****  **
```

```
human           CCTGACTTCTGCCCCCAGAAGTGCCCCAACAACATGCAGTACCACGAGTGCCGCTCCCCC
rabbit          CCTGAC--------------GTGTCCCCACGACATGGAATACCACGAGTGCCGCTCACCC
                ******              *** *** ** ***** * ***************** ***

human           TGTGCAGACACCTGCTCCAACCAGGAGCACTCCCGGGCCTGTGAGGACCACTGTGTGGCC
rabbit          TGCGCCGACACCTGCTCCAACCCCGAGCGCTCCCAGCTCTGCGAGGACCACTGCGTGGCC
                ** ** ****************  **** ***** *  *** *********** ******

human           GGCTGCTTCTGCCCTGAGGGGACGGTGCTTGACGACATCGGCCAGACCGGCTGTGTCCCT
rabbit          GGCTGCTTCTGCCCCGAGGGGACGGTGCTGGACGACATCGGCCAGGCCGGCTGTGTCCCC
                ************** ************** *************** ************* 

human           GTGTCAAAGTGTGCCTGCGTCTACAACGGGGCTGCCTATGCCCCAGGGGCCACCTACTCC
rabbit          GTGACCCAGTGCGCCTGCACCTACAACGGGGCCACCTACGCGTCAGGAGCCAAATACTCC
                *** *  **** ******  ************  **** **  **** ****  ******

human           ACAGACTGCACCAACTGCACCTGCTCCGGAGGCCGGTGGAGCTGCCAGGAGGTTCCATGC
rabbit          ACAGACTGCACCGAGTGCACCTGCTCCGGAGGCCGCTGGAGCTGCCAGGACATCCCATGT
                ************ * ******************** **************  * ***** 

human           CCGGGTACCTGCTCTGTGCTTGGAGGTGCCCACTTCTCAACGTTTGACGGGAAGCAATAC
rabbit          CCAGGCACCTGCTCGGTGCTGGGGGGCGCCCACTTCTCCACGTTCGACGAGAGGCAGTAC
                ** ** ******** ***** ** ** *********** ***** **** ** *** ***

human           ACGGTGCACGGCGACTGCAGCTATGTGCTGACCAAGCCCTGTGACAGCAGTGCCTTCACT
rabbit          ACGGTGCACGGGGACTGCAGCTACGTGCTGGCCAAGTCCTGTAACAGCAGCACCTTCACC
                *********** *********** ****** ***** ***** *******  ******* 

human           GTACTGGCTGAGCTGCGCAGGTGCGGGCTGACGGACAGCGAGACCTGCCTGAAGAGCGTG
rabbit          GTGCTGGCGGAGCTGCGCAGGTGCGGGCTGACGGACAGCGAGACCTGCCTGAAGAGCGTG
                ** ***** ***************************************************

human           ACACTGAGCCTGGATGGGGTGCAGACGGTGGTGGTGATCAAGGCCAGTGGGGAAGTGTTC
rabbit          ACGCTGAACCTGGACGGGGGGCAGACGGTCGTGGTGGTCAAGGCCAGCGGGGAGGTGTTT
                ** **** ****** **** ********* ****** ********** ***** ***** 
```

```
human     CTGAACCAGATCTACACCCAGCTGCCCATCTCTGCAGCCAACGTCACCATCTTCAGACCC
rabbit    GTGAACCATATCTACACCCAGCTGCCTGTCTCCGCAGGTGAG------------------
           ******* *****************  **** ****   *

human     TCAACCTTCTTCATCATCGCCCAGACCAGCCTGGGCCTGCAGCTGAACCTGCAGCTGGTG
rabbit    ------------------------------CTGGGCCTGCAGTTGGACATCCAGCTGGTG
                                        ************ ** ** * *********

human     CCCACCATGCAGCTGTTCATGCAGCTGGCGCCCAAGCTCCGTGGGCAGACCTGCGGTCTC
rabbit    CCCACCATGCAGGTGGCTGTGAGGCTGGAGCCCGAGTTCAGGGGCCGGACCTGCGGTCTC
          ************ **    **  ***** **** ** ** * ** * *************

human     TGTGGGAACTTCAACAGCATCCAGGCCGATGACTTCCGGACCCTCAGTGGGGTGGTGGAG
rabbit    TGCGGGAATTTCAACCGCGTGCAGGCAGACGACTTCCGGGCCATCAGCGGTGTGGTGGAG
          ** ***** ****** ** * ***** ** ********* ** **** ** *********

human     GCCACCGCTGCGGCCTTCTTCAACACCTTCAAGACCCAGGCCGCCTGCCCCAACATCAGG
rabbit    GGCACGGCCGCCGCCTTCTTCAACACCTTCAAGACCCATGCCTCCTGCCCCAACGTCAAG
          * *** ** ** ************************** *** *********** *** *

human     AACAGCTTCGAGGACCCCTGCTCTCTGAGCGTGGAGAATGAGAAGTATGCTCAGCACTGG
rabbit    AACAACTTCGAGGACCCCTGCTCCCTCAGCGTGGAGAACGAGAAGTTTGCCCAGCACTGG
          **** ****************** ** *********** ******* *** *********

human     TGCTCGCAGCTGACCGATGCCGACGGCCCCTTCGGCCGGTGCCATGCTGCCGTGAAGCCG
rabbit    TGCTCGCGGCTGACAGACGCCCAAGGCCCCTTTGCCCAGTGCCACTCCGCCGTGAGCCCT
          ******* ****** ** *** * ******** * ** ******  * *******  **

human     GGCACCTACTACTCGAACTGCATGTTTGACACCTGCAACTGTGAGCGGAGCGAGGACTGC
rabbit    GGCACCTACCACTCGAACTGCCTGTTTGACACCTGCAACTGTGAGAAGACTGAGGACTGC
          ********* *********** ***********************  **  *********

human     CTGTGCGCCGCGCTGTCCTCCTACGTGCACGCCTGTGCCGCCAAGGGCGTGCAGCTCGGC
rabbit    CTGTGTGCGGCGCTGGCCTCCTACGTGCGCGCCTGCGCCGCCAAGGGCGTGCTGCTCCGT
          ***** ** ****** ************ ****** **************** **** *
```

```
human      GGCTGGAGGGACGGCGTCTGCACGAAGCCTATGACCACTTGCCCCAAGTCAATGACGTAC
rabbit     GGCTGGCGGGACGGCGTCTGCACCAAGCACACGAGCAGCTGCCCCAAGTCCATGACCTAC
           ****** **************** ****  * ** **  *********** ***** ***

human      CACTACCATGTCAGCACCTGCCAGCCCACCTGCCGCTCCCTGAGCGAGGGGGACATCACC
rabbit     CACTACCACATCAGCACCTGCCAGCCCACCTGCCGCGCCCGGAGCGAGCCCGACCTCACC
           ********  ************************** *** *******   *** *****

human      TGCAGTGTTGGCTTCATCCCCGTGGATGGCTGCATCTGTCCCAAGGGCACCTTCCTGGAC
rabbit     TGCGGCGTCAGCTTCGTGCCCGTGGACGGCTGCGCCTGCCCCGAGGGCACCTTCCTGGAC
           *** * **  ***** * ******** ******  *** *** *****************

human      GACACGGGCAAGTGTGTGCAGGCCAGCAACTGTCCCTGCTACCACAGAGGCTCCATGATC
rabbit     GACGTGGGCAAGTGTGTGGAGGCCACCAGCTGTCCCTGCTACCACAGCGGCTCCGTGGTC
           ***  ************* ****** ** ****************** ****** ** **

human      CCCAATGGGGAGTCGGTGCACGACAGCGGGGCTATCTGCACCTGCACACATGGGAAGCTG
rabbit     CCCAACGGCGAGTCTGTGCATGACAGTGGGGCCGTCTGCACCTGCACACAGGGGACGCTG
           ***** ** ***** ***** ***** *****  **************** **** ****

human      AGCTGCATCGGAGGCCAAGCCCCCGCCCCAGTGTGTGCTGCGCCCATGGTGTTCTTTGAC
rabbit     ACCTGCATTGGAGGCCCAGCTCCGGCCCCAGTGTGTGACGCACCCATGGTCTTCTTCGAC
           * ****** ******* *** ** *************  ** ******** ***** ***

human      TGCCGAAATGCCACGCCCGGGGACACAGGGGCTGGCTGTCAGAAGAGCTGCCACACACTG
rabbit     TGCCGCAACGCCACGGCCGGGGCCACGGGGGCTGGCTGTCAGAAGAGCTGTCACACCCTG
           ***** ** ****** ****** *** ********************** ***** ***

human      GACATGACCTGTTACAGCCCCCAGTGTGTGCCTGGCTGTGTGTGCCCCGATGGGCTGGTG
rabbit     GACATGGAGTGTTACAGCTCCCAGTGCGTGCCCGGCTGCGTGTGCCCCGACGGGCTGGTG
           ******   ********* ******* ***** ***** *********** *********

human      GCGGATGGCGAGGGCGGCTGCATCACTGCGGAGGACTGCCCCTGCGTGCACAATGAGGCC
rabbit     GCCGACGGCCTCGGCGGCTGCATCGCTGAGGAAGAATGTCCCTGTGTGCACAACGAGGCC
           ** ** ***   ************ *** *** ** ** ***** ******** ******
```

```
human     AGCTACCGGGCCGGCCAGACCATCCGGGTGGGCTGCAACACCTGCACCTGTGACAGCAGG
rabbit    AGCTACGGCCCCGGAGAGACCATCTGGACGGGGTGCAACACCTGCACCTGTGAGAACAGG
          ****** *  ****  ******** **  *** ******************** * ****

human     ATGTGGCGGTGCACAGATGACCCCTGCCTGGCCACCTGCGCCGTGTACGGGGACGGCCAC
rabbit    AAGTGGCGCTGCTCGGACGAGCCCTGCCTGGCCACCTGCGCCGTATACGGG-ACGGTCAC
          * ****** *** * ** ** *********************** ****** **** ***

human     TACCTCACCTT-CGACGGACAGAGCTACAGCTTCAACGGAGACTGCGAGTACACGCTGGT
rabbit    TACTTCACCTTTCGACGG---GGGCTACAGCTTCAGTGGTGACTGCGAGTACACGCTGTT
          *** ******* ******   * ************  ** ****************** *

human     GCAGAACCACTGTGGCGGGAAAGACAGCACCCAGGACTCCTTTCGTGTTGTCACCGAGAA
rabbit    GCAGGACCAGTGCGGTGGGAACGTCAGCGCCCAGGACGCCTTCCGCGTCGTCACGGAGAA
          **** **** ** ** ***** * **** ******** **** ** ** ***** *****

human     CGTCCCCTGCGGCACCACAGGGACCACCTGCTCCAAGGCCATCAAGATTTTCCTGGGGGG
rabbit    CGTCCCCTGCGGCACCACAGGGACCACCTGCTCCAAGGCCATCAAGATCTTCCTGGGGAG
          ************************************************ ********* *

human     CTTCGAGCTGAAGCTAAGCCATGGGAAGGTGGAGGTGATCGGGACGGACGAGAGCCAGGA
rabbit    CTACGAGCTGAAGCTGAGTGACGGGAAGGTGGAGGTGGTGGAGAAAGGCAAGGGCCAGGA
          ** ************ **  * *************** * * **  * * ** *******

human     GGTGCCATACACCATCCGGCAGATGGGCATCTACCTGGTGGTGGACACCGACATTGGCCT
rabbit    GCCGCCCTACTCCATCCGCCAGATGGGCATCTTCCTGGTGCTGGACACCGACGCTGGCCT
          *  *** *** ******* ************* ******* ***********  ******

human     GGTGCTGCTGTGGGACAAGAAGACCAGCATCTTCATCAACCTCAGCCCCGAGTTCAAGGG
rabbit    GGTGCTGCTGTGGGACAGGAGGACCAGCCTCTTCCTCAGGCTCAGCCCCGAGTTCAAGGG
          ***************** ** ******* ***** ***  ********************

human     CAGGGTCTGCGGCCTGTGTGGGAACTTCGACGACATCGCCGTTAATGACTTTGCCACGCG
rabbit    CAGGGTCTGTGGCCTGTGCGGGAACTTCGACGACAACGCCATGAACGACTTCACCACGCG
          ********* ******** **************** **** * ** *****  *******
```

```
human         GAGCCGGTCTGTGGTGGGGGACGTGCTGGAGTTTGGGAACAGCTGGAAGCTCTCCCCCTC
rabbit        GAGCCAGTCGGTGGTGGGCGACGCGCTGGAGTTCGGGAACAGCTGGAAGTTCTCGCCATC
              ***** *** ******** **** ********* *************** **** ** **

human         CTGCCCAGATGCCCTGGCGCCCAAGGACCCCTGCACGGCCAACCCCTTCCGCAAGTCCTG
rabbit        CTGCCCGGACGCGCCGGCACCCAGGGACTCCTGCGCCGCCAACCCCTACCGCAAGTCCTG
              ****** ** ** * *** **** **** ***** * ********** ************

human         GGCCCAGAAGCAGTGCAGCATCCTCCACGGCCCCACCTTCGCCGCCTGCCACGCACACGT
rabbit        GGCCCAGAAGCAGTGCAGCATCGTCCACAGCGCCACCTTCGCTGCCTGCCACGCCCACGT
              ********************** ***** ** ********** *********** *****

human         GGAGCCGGCCAGGTACTACGAGGCCTGCGTGAACGACGCGTGCGCCTGCGACTCCGGGGG
rabbit        GGAGCCTGCCGAGTACTATGAGGCCTGCGTGCAGGACGCCTGCGCCTGCGACTCGGGCGG
              ****** ***  ****** ************ * ***** ************** ** **

human         TGACTGCGAGTGCTTCTGCACGGCTGTGGCCGCCTACGCCCAGGCCTGCCATGAAGTAGG
rabbit        CGACTGCGAGTGCTTCTGCACGGCCGTGGCCGCCTACGCCCAGGCCTGCCACGAGGTGGG
               *********************** ************************** ** ** **

human         CCTGTGTGTGTCCTGGCGGACCCCGAGCATCTGCCCTCTGTTCTGCGACTACTACAACCC
rabbit        CGTGTGCGTGTCCTGGAGGAGCCCCGACGTGTGCCCGCTGTTCTGTGACTTCTACAAAAA
              * **** ********* *** ***   * * ***** ******** **** ******

human         CGAAGGCCAGTGCGAGTGGCACTACCAGCCCTGCGGGGTGCCCTGCCTGCGCACCTGCCG
rabbit        CGAGGGCCAGTGCGAGTGGCACTACCAGCCCTGCGGGGCGCCCTGCATGCGGACGTGCCG
              *** ********************************** ******* **** ** *****

human         GAACCCCCGTGGAGACTGCCTGCGGGACGTCTGGGGCCTGGAAGGCTGCTACCCCAAGTG
rabbit        GAACCCCAGTGGAGAGTGC-----------------------------------------
              ******* ******* ***

human         CCCACCAGAGGCTCCCATCTTTGATGAGGACAAGATGCAGTGTGTGGCCACCTGCCCAAC
rabbit        ------------------------------------------------------------
```

```
human      CCCGCCTCTGCCACCACGGTGCCACGTCCATGGGAAGTCCTACCGGCCAGGTGCAGTGGT
rabbit     ------------------------------------------------------------

human      GCCCTCGGACAAGAACTGCCAGTCCTGCCTTTGTACGGAGCGCGGCGTGGAGTGCACCTA
rabbit     ------------------------------------------------------------

human      CAAAGCTGAGGCCTGTGTCTGCACCTACAATGGACAGCGCTTCCACCCAGGGGACGTCAT
rabbit     ------------------------------------------------------------

human      CTACCACACGACGGATGGCACGGGTGGCTGCATCTCCGCCCGCTGCGGGGCCAACGGCAC
rabbit     ------------------------------------------------------------

human      CATTGAGAGGAGGGTCTACCCCTGCAGCCCCACCACCCCTGTCCCCCCAACCACCTTCTC
rabbit     ------------------------------------------------------------

human      CTTCTCCACACCCCCGCTTGTCGTGAGCTCCACGCACACCCCCAGCAATGGCCCAAGCAG
rabbit     ------------------------------------------------------------

human      CGCGCACACAGGCCCTCCGAGCAGCGCCTGGCCCACCACAGCAGGCACTTCTCCCAGGAC
rabbit     ------------------------------------------------------------

human      GAGGCTGCCCACAGCCTCTGCCTCACTGCCGCCGGTCTGTGGGGAAAAGTGCCTGTGGTC
rabbit     ------------------------------------------------------------

human      GCCATGGATGGATGTCAGCCGCCCTGGACGGGGCACGGACAGCGGTGACTTCGACACACT
rabbit     ------------------------------------------------------------
```

```
human     GGAGAACCTCCGCGCCCATGGGTACCGGGTGTGCGAATCACCCAGGTCGGTGGAGTGCCG
rabbit    ------------------------------------------------------------

human     AGCTGAGGACGCCCCCGGAGTGCCGCTCCGAGCCCTGGGGCAGCGTGTGCAGTGCAGCCC
rabbit    ------------------------------------------------------------

human     GGATGTGGGGCTGACCTGTCGTAACAGGGAGCAGGCATCGGGGCTCTGCTACAACTACCA
rabbit    ------------------------------------------------------------

human     GATCAGGGTCCAGTGCTGCACGCCCCTAGCCTGCTCCACCTCTAGCAGTCCAGCCCAGAC
rabbit    ------------------------------------------------------------

human     CACTCCTCCAACTACCTCCAAGACCACTGAAACCCGGGCCTCAGGCTCCTCAGCTCCCAG
rabbit    ------------------------------------------------------------

human     CAGCACACCTGGCACCGTGTCTCTCTCTACAGCCAGGACGACACCTGCCCCAGGTACCGC
rabbit    ------------------------------------------------------------

human     TACCTCTGTCAAAAAAACTTTCTCAACTCCCAGCCCTCCGCCAGTGCCGGCAACATCAAC
rabbit    ------------------------------------------------------------

human     ATCATCCATGTCGACCACGACCCCGGGGACCTCTGTGGTCTCCAGCAAGCCCACCCTCAC
rabbit    ------------------------------------------------------------

human     TGAGCCCAGCACATCCTCCTGCCTGCAGGAGCTTTGCACCTGGACCGAGTGGATCGATGG
rabbit    ------------------------------------------------------------
```

```
human           CAGCTACCCTGCTCCTGGAATAAATGGTGGAGATTTTGACACATTTCAAAATTTGAGAGA
rabbit          ------------------------------------------------------------

human           CGAAGGATACACATTCTGTGAAAGTCCTCGAAGCGTGCAGTGCCGGGCAGAGAGCTTCCC
rabbit          ------------------------------------------------------------

human           CAACACGCCGCTGGCAGACCTGGGGCAGGACGTCATCTGCAGCCACACAGAGGGGCTGAT
rabbit          ------------------------------------------------------------

human           TTGCCTGAACAAGAACCAGCTCCCACCCATCTGCTACAACTATGAGATCCGCATCCAGTG
rabbit          ------------------------------------------------------------

human           TTGCGAGACGGTGAACGTGTGCAGAGACATCACCAGACCGCCAAAGACCGTCGCAACGAC
rabbit          ------------------------------------------------------------

human           ACGGCCGACTCCACATCCAACCGGAGCTCAGACCCAGACCACCTTCACCACACACATGCC
rabbit          ------------------------------------------------------------

human           CTCGGCCTCCACAGAGCAACCCACGGCAACCTCCAGGGGTGGGCCCACAGCAACCAGCGT
rabbit          ------------------------------------------------------------

human           CACACAGGGCACCCACACCACACCAGTCACCAGAAACTGTCATCCCCGGTGCACCTGGAC
rabbit          ------------------------------------------------------------

human           AAAGTGGTTCGACGTGGACTTCCCGTCCCCCGGACCCCATGGTGGAGACAAGGAAACCTA
rabbit          ------------------------------------------------------------
```

```
human       CAACAACATCATCAGGAGTGGGGAAAAAATCTGCCGCCGACCTGAGGAGATCACCAGGCT
rabbit      ------------------------------------------------------------

human       CCAGTGCCGAGCCAAGAGCCACCCAGAGGTGAGCATCGAACACCTGGGCCAGGTGGTGCA
rabbit      ------------------------------------------------------------

human       GTGCAGCCGGGAAGAGGGCCTGGTGTGCCGGAACCAGGACCAGCAGGGACCCTTCAAGAT
rabbit      ------------------------------------------------------------

human       GTGCCTCAACTACGAGGTGCGTGTGCTCTGCTGCGAGACCCCCAGAGGCTGCCCCGTGAC
rabbit      ------------------------------------------------------------

human       CTCCACACCTGTGACAGCTCCTAGCACCCCTAGTGGGAGAGCCATCAGCCCAACTCAGAG
rabbit      ------------------------------------------------------------

human       CACCTCCTCTTGGCAGAAATCCAGGACAACCACTTTGGTGACAACCAGCACAACCTCCAC
rabbit      ------------------------------------------------------------

human       TCCACAGACCAGTACAACCTATGCCCATACAACCAGCACAACCTCTGCTCCTACAGCCAG
rabbit      ------------------------------------------------------------

human       AACAACCTCTGCTCCTACAACCAGAACAACCTCTGCCTCTCCAGCCAGCACAACCTCTGG
rabbit      ------------------------------------------------------------

human       TCCTGGAAATACTCCCAGCCCTGTTCCTACCACCAGCACAATCTCTGCTCCTACAACTAG
rabbit      ------------------------------------------------------------
```

```
human      CATAACCTCTGCCCCTACAACCAGCACAACCTCTGCCCCTACAAGCAGCACAACCTCTGC
rabbit     ------------------------------------------------------------

human      TCCTACAACCAGCACAACCTCTGCCCCTACAAGCAGCACAACCTCCAGTCCACAGACCAG
rabbit     ------------------------------------------------------------

human      CACAACCTCGGCTCCTACAACCAGCACAACTTCTGGTCCTGGAACTACCCCAAGCCCTGT
rabbit     ------------------------------------------------------------

human      TCCCACGACCAGC
rabbit     -------------
```

Underline fields are primer sequences we used.

Supplemental Information 4.

CLUSTAL 2.1 multiple sequence alignment

Muc16

```
human           AAGCGTTGCACAATTCCCCCAACCTCCATACATACGGCAGCTCTTCTAGACACAGGTTTT
rabbit          ------------------------------------------------------------


human           CCCAGGTCAAATGCGGGGACCCCAGCCATATCTCCCACCCTGAGAAATTTTGGAGTTTCA
rabbit          ------------------------------------------------------------


human           GGGAGCTCAGAAGCTCTGCAGAGGCCACCCTCTCTGAGGGGATTCTTCTTAGACCTCCAT
rabbit          ------------------------------------------------------------


human           CCAGAGGCAAATGTTGACCTGTCCATGCTGAAACCCTCAGGCCTTCCTGGGTCATCTTCT
rabbit          ------------------------------------------------------------


human           CCCACCCGCTCCTTGATGACAGGGAGCAGGAGCACTAAAGCCACACCAGAAATGGATTCA
rabbit          ------------------------------------------------------------


human           GGACTGACAGGAGCCACCTTGTCACCTAAGACATCTACAGGTGCAATCGTGGTGACAGAA
rabbit          ------------------------------------------------------------


human           CATACTCTGCCCTTTACTTCCCCAGATAAGACCTTGGCCAGTCCTACATCTTCGGTTGTG
rabbit          ------------------------------------------------------------


human           GGAAGAACCACCCAGTCTTTGGGGGTGATGTCCTCTGCTCTCCCTGAGTCAACCTCTAGA
rabbit          ------------------------------------------------------------
```

```
human           GGAATGACACACTCCGAGCAAAGAACCAGCCCATCGCTGAGTCCCCAGGTCAATGGAACT
rabbit          ------------------------------------------------------------

human           CCCTCTAGGAACTACCCTGCTACAAGCATGGTTTCAGGATTGAGTTCCCCAAGGACCAGG
rabbit          ------------------------------------------------------------

human           ACCAGTTCCACAGAAGGAAATTTTACCAAAGAAGCATCTACATACACACTCACTGTAGAG
rabbit          ------------------------------------------------------------

human           ACCACAAGTGGCCCAGTCACTGAGAAGTACACAGTCCCCACTGAGACCTCAACAACTGAA
rabbit          ------------------------------------------------------------

human           GGTGACAGCACAGAGACCCCCTGGGACACAAGATATATTCCTGTAAAAATCACATCTCCA
rabbit          ------------------------------------------------------------

human           ATGAAAACATTTGCAGATTCAACTGCATCCAAGGAAAATGCCCCAGTGTCTATGACTCCA
rabbit          ------------------------------------------------------------

human           GCTGAGACCACAGTTACTGACTCACATACTCCAGGAAGGACAAACCCATCATTTGGGACA
rabbit          ------------------------------------------------------------

human           CTTTATTCTTCCTTCCTTGACCTATCACCTAAAGGGACCCCAAATTCCAGAGGTGAAACA
rabbit          ------------------------------------------------------------

human           AGCCTGGAACTGATTCTATCAACCACTGGATATCCCTTCTCCTCTCCTGAACCTGGCTCT
rabbit          ------------------------------------------------------------
```

```
human     GCAGGACACAGCAGAATAAGTACCAGTGCGCCTTTGTCATCATCTGCTTCAGTTCTCGAT
rabbit    ------------------------------------------------------------

human     AATAAAATATCAGAGACCAGCATATTCTCAGGCCAGAGTCTCACCTCCCCTCTGTCTCCT
rabbit    ------------------------------------------------------------

human     GGGGTGCCCGAGGCCAGAGCCAGCACAATGCCCAACTCAGCTATCCCTTTTTCCATGACA
rabbit    ------------------------------------------------------------

human     CTAAGCAATGCAGAAACAAGTGCCGAAAGGGTCAGAAGCACAATTTCCTCTCTGGGGACT
rabbit    ------------------------------------------------------------

human     CCATCAATATCCACAAAGCAGACAGCAGAGACTATCCTTACCTTCCATGCCTTCGCTGAG
rabbit    ------------------------------------------------------------

human     ACCATGGATATACCCAGCACCCACATAGCCAAGACTTTGGCTTCAGAATGGTTGGGAAGT
rabbit    ------------------------------------------------------------

human     CCAGGTACCCTTGGTGGCACCAGCACTTCAGCGCTGACAACCACATCTCCATCTACCACT
rabbit    ------------------------------------------------------------

human     TTAGTCTCAGAGGAGACCAACACCCATCACTCCACGAGTGGAAAGGAAACAGAAGGAACT
rabbit    ------------------------------------------------------------

human     TTGAATACATCTATGACTCCACTTGAGACCTCTGCTCCTGGAGAAGAGTCCGAAATGACT
rabbit    ------------------------------------------------------------
```

```
human     GCCACCTTGGTCCCCACTCTAGGTTTTACAACTCTTGACAGCAAGATCAGAAGTCCATCT
rabbit    ------------------------------------------------------------

human     CAGGTCTCTTCATCCCACCCAACAAGAGAGCTCAGAACCACAGGCAGCACCTCTGGGAGG
rabbit    ------------------------------------------------------------

human     CAGAGTTCCAGCACAGCTGCCCACGGGAGCTCTGACATCCTGAGGGCAACCACTTCCAGC
rabbit    ------------------------------------------------------------

human     ACCTCAAAAGCATCATCATGGACCAGTGAAAGCACAGCTCAGCAATTTAGTGAACCCCAG
rabbit    ------------------------------------------------------------

human     CACACACAGTGGGTGGAGACAAGTCCTAGCATGAAAACAGAGAGACCCCCAGCATCAACC
rabbit    ------------------------------------------------------------

human     AGTGTGGCAGCCCCTATCACCACTTCTGTTCCCTCAGTGGTCTCTGGCTTCACCACCCTG
rabbit    ------------------------------------------------------------

human     AAGACCAGCTCCACAAAAGGGATTTGGCTTGAAGAAACATCTGCAGACACACTCATCGGA
rabbit    ------------------------------------------------------------

human     GAATCCACAGCTGGCCCAACCACCCATCAGTTTGCTGTTCCCACTGGGATTTCAATGACA
rabbit    ------------------------------------------------------------

human     GGAGGCAGCAGCACCAGGGGAAGCCAGGGCACAACCCACCTACTCACCAGAGCCACAGCA
rabbit    ------------------------------------------------------------
```

```
human     TCATCTGAGACATCCGCAGATTTGACTCTGGCCACGAACGGTGTCCCAGTCTCCGTGTCT
rabbit    ------------------------------------------------------------

human     CCAGCAGTGAGCAAGACGGCTGCTGGCTCAAGTCCTCCAGGAGGGACAAAGCCATCATAT
rabbit    ------------------------------------------------------------

human     ACAATGGTTTCTTCTGTCATCCCTGAGACATCATCTCTACAGTCCTCAGCTTTCAGGGAA
rabbit    ------------------------------------------------------------

human     GGAACCAGCCTGGGACTGACTCCATTAAACACTAGACATCCCTTCTCTTCCCCTGAACCA
rabbit    ------------------------------------------------------------

human     GACTCTGCAGGACACACCAAGATAAGCACCAGCATTCCTCTGTTGTCATCTGCTTCAGTT
rabbit    ------------------------------------------------------------

human     CTTGAGGATAAAGTGTCAGCGACCAGCACATTCTCACACCACAAAGCCACCTCATCTATT
rabbit    ------------------------------------------------------------

human     ACCACAGGGACTCCTGAAATCTCAACAAAGACAAAGCCCAGCTCAGCCGTTCTTTCCTCC
rabbit    ------------------------------------------------------------

human     ATGACCCTAAGCAATGCAGCAACAAGTCCTGAAAGAGTCAGAAATGCAACTTCCCCTCTG
rabbit    ------------------------------------------------------------

human     ACTCATCCATCTCCATCAGGGGAAGAGACAGCAGGGAGTGTCCTCACTCTCAGCACCTCT
rabbit    ------------------------------------------------------------
```

```
human        GCTGAGACTACAGACTCACCTAACATCCACCCAACTGGGACACTGACTTCAGAATCGTCA
rabbit       ------------------------------------------------------------

human        GAGAGTCCTAGCACTCTCAGCCTCCCAAGTGTCTCTGGAGTCAAAACCACATTTTCTTCA
rabbit       ------------------------------------------------------------

human        TCTACTCCTTCCACTCATCTATTTACTAGTGGAGAAGAAACAGAGGAAACTTCGAATCCA
rabbit       ------------------------------------------------------------

human        TCTGTGTCTCAACCTGAGACTTCTGTTTCCAGAGTAAGGACCACCTTGGCCAGCACCTCT
rabbit       ------------------------------------------------------------

human        GTCCCTACCCCAGTATTCCCCACCATGGACACCTGGCCTACACGTTCAGCTCAGTTCTCT
rabbit       ------------------------------------------------------------

human        TCATCCCACCTAGTGAGTGAGCTCAGAGCTACGAGCAGTACCTCAGTTACAAACTCAACT
rabbit       ------------------------------------------------------------

human        GGTTCAGCTCTTCCTAAAATATCTCACCTCACTGGGACGGCAACAATGTCACAGACCAAT
rabbit       ------------------------------------------------------------

human        AGAGACACGTTTAATGACTCTGCTGCACCCCAAAGCACAACTTGGCCAGAGACTAGTCCC
rabbit       ------------------------------------------------------------

human        AGATTCAAGACAGGGTTACCTTCAGCAACAACCACTGTTTCAACCTCTGCCACTTCTCTC
rabbit       ------------------------------------------------------------
```

```
human         TCTGCTACTGTAATGGTCTCTAAATTCACTTCTCCAGCAACTAGTTCCATGGAAGCAACT
rabbit        ------------------------------------------------------------

human         TCTATCAGGGAACCATCAACAACCATCCTCACAACAGAGACCACGAATGGCCCAGGCTCT
rabbit        ------------------------------------------------------------

human         ATGGCTGTGGCTTCTACCAACATCCCAATTGGAAAGGGCTACATTACTGAAGGAAGATTG
rabbit        ------------------------------------------------------------

human         GACACAAGCCATCTGCCCATTGGAACCACAGCTTCCTCTGAGACATCTATGGATTTTACC
rabbit        ------------------------------------------------------------

human         ATGGCCAAAGAAAGTGTCTCAATGTCAGTATCTCCATCTCAGTCCATGGATGCTGCTGGC
rabbit        ------------------------------------------------------------

human         TCAAGCACTCCAGGAAGGACAAGCCAATTCGTTGACACATTTTCTGATGATGTCTATCAT
rabbit        ------------------------------------------------------------

human         TTAACATCCAGAGAAATTACAATACCTAGAGATGGAACAAGCTCAGCTCTGACTCCACAA
rabbit        ------------------------------------------------------------

human         ATGACTGCAACTCACCCTCCATCTCCTGATCCTGGCTCTGCTAGAAGCACCTGGCTTGGC
rabbit        ------------------------------------------------------------

human         ATCTTGTCCTCATCTCCTTCTTCTCCTACTCCCAAAGTCACAATGAGCTCCACATTTTCA
rabbit        ------------------------------------------------------------
```

```
human           ACTCAGAGAGTCACCACAAGCATGATAATGGACACAGTTGAAACTAGTCGGTGGAACATG
rabbit          ------------------------------------------------------------

human           CCCAACTTACCTTCCACGACTTCCTTGACACCAAGTAATATTCCAACAAGTGGTGCCATA
rabbit          ------------------------------------------------------------

human           GGAAAAAGCACCCTGGTTCCCTTGGACACTCCATCTCCAGCCACATCATTGGAGGCATCA
rabbit          ------------------------------------------------------------

human           GAAGGGGGACTTCCAACCCTCAGCACCTACCCTGAATCAACAAACACACCCAGCATCCAC
rabbit          ------------------------------------------------------------

human           CTCGGAGCACACGCTAGTTCAGAAAGTCCAAGCACCATCAAACTTACCATGGCTTCAGTA
rabbit          ------------------------------------------------------------

human           GTAAAACCTGGCTCTTACACACCTCTCACCTTCCCCTCAATAGAGACCCACATTCATGTA
rabbit          ------------------------------------------------------------

human           TCAACAGCCAGAATGGCTTACTCTTCTGGGTCTTCACCTGAGATGACAGCTCCTGGAGAG
rabbit          ------------------------------------------------------------

human           ACTAACACTGGTAGTACCTGGGACCCCACCACCTACATCACCACTACGGATCCTAAGGAT
rabbit          ------------------------------------------------------------

human           ACAAGTTCAGCTCAGGTCTCTACACCCCACTCAGTGAGGACACTCAGAACCACAGAAAAC
rabbit          ------------------------------------------------------------
```

```
human       CATCCAAAGACAGAGTCCGCCACCCCAGCTGCTTACTCTGGAAGTCCTAAAATCTCAAGT
rabbit      ------------------------------------------------------------

human       TCACCCAATCTCACCAGTCCGGCCACAAAAGCATGGACCATCACAGACACAACTGAACAC
rabbit      ------------------------------------------------------------

human       TCCACTCAATTACATTACACAAAATTGGCAGAAAAATCATCTGGATTTGAGACACAGTCA
rabbit      ------------------------------------------------------------

human       GCTCCAGGACCTGTCTCTGTAGTAATCCCTACCTCCCCTACCATTGGAAGCAGCACATTG
rabbit      ------------------------------------------------------------

human       GAACTAACTTCTGATGTCCCAGGGGAACCCCTGGTCCTTGCTCCCAGTGAGCAGACCACA
rabbit      ------------------------------------------------------------

human       ATCACTCTCCCCATGGCAACATGGCTGAGTACCAGTTTGACAGAGGAAATGGCTTCAACA
rabbit      ------------------------------------------------------------

human       GACCTTGATATTTCAAGTCCAAGTTCACCCATGAGTACATTTGCTATTTTTCCACCTATG
rabbit      ------------------------------------------------------------

human       TCCACACCTTCTCATGAACTTTCAAAGTCAGAGGCAGATACCAGTGCCATTAGAAATACA
rabbit      ------------------------------------------------------------

human       GATTCAACAACGTTGGATCAGCACCTAGGAATCAGGAGTTTGGGCAGAACTGGGGACTTA
rabbit      ------------------------------------------------------------
```

```
human     ACAACTGTTCCTATCACCCCACTGACAACCACGTGGACCAGTGTGATTGAACACTCAACA
rabbit    ------------------------------------------------------------

human     CAAGCACAGGACACCCTTTCTGCAACGATGAGTCCTACTCACGTGACACAGTCACTCAAA
rabbit    ------------------------------------------------------------

human     GATCAAACATCTATACCAGCCTCAGCATCCCCTTCCCATCTTACTGAAGTCTACCCTGAG
rabbit    ------------------------------------------------------------

human     CTCGGGACACAAGGGAGAAGCTCCTCTGAGGCAACCACTTTTTGGAAACCATCTACAGAC
rabbit    ------------------------------------------------------------

human     ACACTGTCCAGAGAGATTGAGACTGGCCCAACAAACATTCAATCCACTCCACCCATGGAC
rabbit    ------------------------------------------------------------

human     AACACAACAACAGGGAGCAGTAGTAGTGGAGTCACCCTGGGCATAGCCCACCTTCCCATA
rabbit    ------------------------------------------------------------

human     GGAACATCCTCCCCAGCTGAGACATCCACAAACATGGCACTGGAAAGAAGAAGTTCTACA
rabbit    ------------------------------------------------------------

human     GCCACTGTCTCTATGGCTGGGACAATGGGACTCCTTGTTACTAGTGCTCCAGGAAGAAGC
rabbit    ------------------------------------------------------------

human     ATCAGCCAGTCATTAGGAAGAGTTTCCTCTGTCCTTTCTGAGTCAACTACTGAAGGAGTC
rabbit    ------------------------------------------------------------
```

```
human           ACAGATTCTAGTAAGGGAAGCAGCCCAAGGCTGAACACACAGGGAAATACAGCTCTCTCC
rabbit          ------------------------------------------------------------

human           TCCTCTCTTGAACCCAGCTATGCTGAAGGAAGCCAGATGAGCACAAGCATCCCTCTAACC
rabbit          ------------------------------------------------------------

human           TCATCTCCTACAACTCCTGATGTGGAATTCATAGGGGGCAGCACATTTTGGACCAAGGAG
rabbit          ------------------------------------------------------------

human           GTCACCACAGTTATGACCTCAGACATCTCCAAGTCTTCAGCAAGGACAGAGTCCAGCTCA
rabbit          ------------------------------------------------------------

human           GCTACCCTTATGTCCACAGCTTTGGGAAGCACTGAAAATACAGGAAAAGAAAAACTCAGA
rabbit          ------------------------------------------------------------

human           ACTGCCTCTATGGATCTTCCATCTCCAACTCCATCAATGGAGGTGACACCATGGATTTCT
rabbit          ------------------------------------------------------------

human           CTCACTCTCAGTAATGCCCCCAATACCACAGATTCACTTGACCTCAGCCATGGGGTGCAC
rabbit          ------------------------------------------------------------

human           ACCAGCTCTGCAGGGACTTTGGCCACTGACAGGTCATTGAATACTGGTGTCACTAGAGCC
rabbit          ------------------------------------------------------------

human           TCCAGATTGGAAAACGGCTCTGATACCTCTTCTAAGTCCCTGTCTATGGGAAACAGCACT
rabbit          ------------------------------------------------------------
```

```
human        CACACTTCCATGACTTACACAGAGAAGAGTGAAGTGTCTTCTTCAATCCATCCCCGACCT
rabbit       ------------------------------------------------------------

human        GAGACCTCAGCTCCTGGAGCAGAGACCACTTTGACTTCCACTCCTGGAAACAGGGCCATA
rabbit       ------------------------------------------------------------

human        AGCTTAACATTGCCTTTTTCATCCATTCCAGTGGAAGAAGTCATTTCTACAGGCATAACC
rabbit       ------------------------------------------------------------

human        TCAGGACCAGACATCAACTCAGCACCCATGACACATTCTCCCATCACCCCACCAACAATT
rabbit       ------------------------------------------------------------

human        GTATGGACCAGTACAGGCACAATTGAACAGTCCACTCAACCACTACATGCAGTTTCTTCA
rabbit       ------------------------------------------------------------

human        GAAAAAGTTTCTGTGCAGACACAGTCAACTCCATATGTCAACTCTGTGGCAGTGTCTGCT
rabbit       ------------------------------------------------------------

human        TCCCCTACCCATGAGAATTCAGTCTCTTCTGGAAGCAGCACATCCTCTCCATATTCCTCA
rabbit       ------------------------------------------------------------

human        GCCTCACTTGAATCCTTGGATTCCACAATCAGTAGGAGGAATGCAATCACTTCCTGGCTA
rabbit       ------------------------------------------------------------

human        TGGGACCTCACTACATCTCTCCCCACTACAACTTGGCCAAGTACTAGTTTATCTGAGGCA
rabbit       ------------------------------------------------------------
```

```
human     CTGTCCTCAGGCCATTCTGGGGTTTCAAACCCAAGTTCAACTACGACTGAATTTCCACTC
rabbit    ------------------------------------------------------------

human     TTTTCAGCTGCATCCACATCTGCTGCTAAGCAAAGAAATCCAGAAACAGAGACCCATGGT
rabbit    ------------------------------------------------------------

human     CCCCAGAATACAGCCGCGAGTACTTTGAACACTGATGCATCCTCGGTCACAGGTCTTTCT
rabbit    ------------------------------------------------------------

human     GAGACTCCTGTGGGGGCAAGTATCAGCTCTGAAGTCCCTCTTCCAATGGCCATAACTTCT
rabbit    ------------------------------------------------------------

human     AGATCAGATGTTTCTGGCCTTACATCTGAGAGTACTGCTAACCCGAGTTTAGGCACAGCC
rabbit    ------------------------------------------------------------

human     TCTTCAGCAGGGACCAAATTAACTAGGACAATATCCCTGCCCACTTCAGAGTCTTTGGTT
rabbit    ------------------------------------------------------------

human     TCCTTTAGAATGAACAAGGATCCATGGACAGTGTCAATCCCTTTGGGGTCCCATCCAACT
rabbit    ------------------------------------------------------------

human     ACTAATACAGAAACAAGCATCCCAGTAAACAGCGCAGGTCCACCTGGCTTGTCCACAGTA
rabbit    ------------------------------------------------------------

human     GCATCAGATGTAATTGACACACCTTCAGATGGGGCTGAGAGTATTCCCACTGTCTCCTTT
rabbit    ------------------------------------------------------------
```

```
human      TCCCCCTCCCCTGATACTGAAGTGACAACTATCTCACATTTCCCAGAAAAGACAACTCAT
rabbit     ------------------------------------------------------------

human      TCATTTAGAACCATTTCATCTCTCACTCATGAGTTGACTTCAAGAGTGACACCTATTCCT
rabbit     ------------------------------------------------------------

human      GGGGATTGGATGAGTTCAGCTATGTCTACAAAGCCCACAGGAGCCAGTCCCTCCATTACA
rabbit     ------------------------------------------------------------

human      CTGGGAGAGAGAAGGACAATCACCTCTGCTGCTCCAACCACTTCCCCCATAGTTCTCACT
rabbit     ------------------------------------------------------------

human      GCTAGTTTCACAGAGACCAGCACAGTTTCACTGGATAATGAAACTACAGTAAAAACCTCA
rabbit     ------------------------------------------------------------

human      GATATCCTTGACGCACGGAAAACAAATGAGCTCCCCTCAGATAGCAGTTCTTCTTCTGAT
rabbit     ------------------------------------------------------------

human      CTGATCAACACCTCCATAGCTTCTTCAACTATGGATGTCACTAAAACAGCCTCCATCAGT
rabbit     ------------------------------------------------------------

human      CCCACTAGCATCTCAGGAATGACAGCAAGTTCCTCCCCATCTCTCTTCTCTTCAGATAGA
rabbit     ------------------------------------------------------------

human      CCCCAGGTTCCCACATCTACAACAGAGACAAATACAGCCACCTCTCCATCTGTTTCCAGT
rabbit     ------------------------------------------------------------
```

```
human       AACACCTATTCTCTTGATGGGGGCTCCAATGTGGGTGGCACTCCATCCACTTTACCACCC
rabbit      ------------------------------------------------------------

human       TTTACAATCACCCACCCTGTCGAGACAAGCTCGGCCCTATTAGCCTGGTCTAGACCAGTA
rabbit      ------------------------------------------------------------

human       AGAACTTTCAGCACCATGGTCAGCACTGACACTGCCTCCGGAGAAAATCCTACCTCTAGC
rabbit      ------------------------------------------------------------

human       AATTCTGTGGTGACTTCTGTTCCAGCACCAGGTACATGGACCAGTGTAGGCAGTACTACT
rabbit      ------------------------------------------------------------

human       GACTTACCTGCCATGGGCTTTCTCAAGACAAGTCCTGCAGGAGAGGCACACTCACTTCTA
rabbit      ------------------------------------------------------------

human       GCATCAACTATTGAACCAGCCACTGCCTTCACTCCCCATCTCTCAGCAGCAGTGGTCACT
rabbit      ------------------------------------------------------------

human       GGATCCAGTGCTACATCAGAAGCCAGTCTTCTCACTACGAGTGAAAGCAAAGCCATTCAT
rabbit      ------------------------------------------------------------

human       TCTTCACCACAGACCCCAACTACACCCACCTCTGGAGCAAACTGGGAAACTTCAGCTACT
rabbit      ------------------------------------------------------------

human       CCTGAGAGCCTTTTGGTAGTCACTGAGACTTCAGACACAACACTTACCTCAAAGATTTTG
rabbit      ------------------------------------------------------------
```

```
human      GTCACAGATACCATCTTGTTTTCAACTGTGTCCACGCCACCTTCTAAATTTCCAAGTACG
rabbit     ------------------------------------------------------------

human      GGGACTCTGTCTGGAGCTTCCTTCCCTACTTTACTCCCGGACACTCCAGCCATCCCTCTC
rabbit     ------------------------------------------------------------

human      ACTGCCACTGAGCCAACAAGTTCATTAGCTACATCCTTTGATTCCACCCCACTGGTGACT
rabbit     ------------------------------------------------------------

human      ATAGCTTCGGATAGTCTTGGCACAGTCCCAGAGACTACCCTGACCATGTCAGAGACCTCA
rabbit     ------------------------------------------------------------

human      AATGGTGATGCACTGGTTCTTAAGACAGTAAGTAACCCAGATAGGAGCATCCCTGGAATC
rabbit     ------------------------------------------------------------

human      ACTATCCAAGGAGTAACAGAAAGTCCACTCCATCCTTCTTCCACTTCCCCCTCTAAGATT
rabbit     ------------------------------------------------------------

human      GTTGCTCCACGGAATACAACCTATGAAGGTTCGATCACAGTGGCACTTTCTACTTTGCCT
rabbit     ------------------------------------------------------------

human      GCGGGAACTACTGGTTCCCTTGTATTCAGTCAGAGTTCTGAAAACTCAGAGACAACGGCT
rabbit     ------------------------------------------------------------

human      TTGGTAGACTCATCAGCTGGGCTTGAGAGGGCATCTGTGATGCCACTAACCACAGGAAGC
rabbit     ------------------------------------------------------------
```

```
human           CAGGGTATGGCTAGCTCTGGAGGAATCAGAAGTGGGTCCACTCACTCAACTGGAACCAAA
rabbit          ------------------------------------------------------------

human           ACATTTTCTTCTCTCCCTCTGACCATGAACCCAGGTGAGGTTACAGCCATGTCTGAAATC
rabbit          ------------------------------------------------------------

human           ACCACGAACAGACTGACAGCTACTCAATCAACAGCACCCAAAGGGATACCTGTGAAGCCC
rabbit          ------------------------------------------------------------

human           ACCAGTGCTGAGTCAGGCCTCCTAACACCTGTCTCTGCCTCCTCAAGCCCATCAAAGGCC
rabbit          ------------------------------------------------------------

human           TTTGCCTCACTGACTACAGCTCCCCCAACTTGGGGGATCCCACAGTCTACCTTGACATTT
rabbit          ------------------------------------------------------------

human           GAGTTTTCTGAGGTCCCAAGTTTGGATACTAAGTCCGCTTCTTTACCAACTCCTGGACAG
rabbit          ------------------------------------------------------------

human           TCCCTGAACACCATTCCAGACTCAGATGCAAGCACAGCATCTTCCTCACTGTCCAAGTCT
rabbit          ------------------------------------------------------------

human           CCAGAAAAAAACCCAAGGGCAAGGATGATGACTTCCACAAAGGCCATAAGTGCAAGCTCA
rabbit          ------------------------------------------------------------

human           TTTCAATCAACAGGTTTTACTGAAACCCCTGAGGGATCTGCCTCCCCTTCTATGGCAGGG
rabbit          ------------------------------------------------------------
```

```
human     CATGAACCCAGAGTCCCCACTTCAGGAACAGGGGACCCTAGATATGCCTCAGAGAGCATG
rabbit    ------------------------------------------------------------

human     TCTTATCCAGACCCAAGCAAGGCATCATCAGCTATGACATCGACCTCTCTTGCATCAAAA
rabbit    ------------------------------------------------------------

human     CTCACAACTCTCTTCAGCACAGGTCAAGCAGCAAGGTCTGGTTCTAGTTCCTCTCCCATA
rabbit    ------------------------------------------------------------

human     AGCCTATCCACTGAGAAAGAAACAAGCTTCCTTTCCCCCACTGCATCCACCTCCAGAAAG
rabbit    ------------------------------------------------------------

human     ACTTCACTATTTCTTGGGCCTTCCATGGCAAGGCAGCCCAACATATTGGTGCATCTTCAG
rabbit    ------------------------------------------------------------

human     ACTTCAGCTCTGACACTTTCTCCAACATCCACTCTAAATATGTCCCAGGAGGAGCCTCCT
rabbit    ------------------------------------------------------------

human     GAGTTAACCTCAAGCCAGACCATTGCAGAAGAAGAGGGAACAACAGCTGAAACACAGACG
rabbit    ------------------------------------------------------------

human     TTAACCTTCACACCATCTGAGACCCCAACATCCTTGTTACCTGTCTCTTCTCCCACAGAA
rabbit    ------------------------------------------------------------

human     CCCACAGCCAGAAGAAAGAGTTCTCCAGAAACATGGGCAAGCTCTATTTCAGTTCCTGCC
rabbit    ------------------------------------------------------------
```

```
human           AAGACCTCCTTGGTTGAAACAACTGATGGAACGCTAGTGACCACCATAAAGATGTCAAGC
rabbit          ------------------------------------------------------------

human           CAGGCAGCACAAGGAAATTCCACGTGGCCTGCCCCAGCAGAGGAGACGGGGAGCAGTCCA
rabbit          ------------------------------------------------------------

human           GCAGGCACATCCCCAGGAAGCCCAGAAATGTCTACCACTCTCAAAATCATGAGCTCCAAG
rabbit          ------------------------------------------------------------

human           GAACCCAGCATCAGCCCAGAGATCAGGTCCACTGTGAGAAATTCTCCTTGGAAGACTCCA
rabbit          ------------------------------------------------------------

human           GAAACAACTGTTCCCATGGAGACCACAGTGGAACCAGTCACCCTTCAGTCCACAGCCCTA
rabbit          ------------------------------------------------------------

human           GGAAGTGGCAGCACCAGCATCTCTCACCTGCCCACAGGAACCACATCACCAACCAAGTCA
rabbit          ------------------------------------------------------------

human           CCAACAGAAAATATGTTGGCTACAGAAAGGGTCTCCCTCTCCCCATCCCCACCTGAGGCT
rabbit          ------------------------------------------------------------

human           TGGACCAACCTTTATTCTGGAACTCCAGGAGGGACCAGGCAGTCACTGGCCACAATGTCC
rabbit          ------------------------------------------------------------

human           TCTGTCTCCCTAGAGTCACCAACTGCTAGAAGCATCACAGGGACTGGTCAGCAAAGCAGT
rabbit          ------------------------------------------------------------
```

```
human          CCAGAACTGGTTTCAAAGACAACTGGAATGGAATTCTCTATGTGGCATGGCTCTACTGGA
rabbit         ------------------------------------------------------------

human          GGGACCACAGGGGACACACATGTCTCTCTGAGCACATCTTCCAATATCCTTGAAGACCCT
rabbit         ------------------------------------------------------------

human          GTAACCAGCCCAAACTCTGTGAGCTCATTGACAGATAAATCCAAACATAAAACCGAGACA
rabbit         ------------------------------------------------------------

human          TGGGTAAGCACCACAGCCATTCCCTCCACTGTCCTGAATAATAAGATAATGGCAGCTGAA
rabbit         ------------------------------------------------------------

human          CAACAGACAAGTCGATCTGTGGATGAGGCTTATTCATCAACTAGTTCTTGGTCAGATCAG
rabbit         ------------------------------------------------------------

human          ACATCTGGGAGTGACATCACCCTTGGTGCATCTCCTGATGTCACAAACACATTATACATC
rabbit         ------------------------------------------------------------

human          ACCTCCACAGCACAAACCACCTCACTAGTGTCTCTGCCCTCTGGAGACCAAGGCATTACA
rabbit         ------------------------------------------------------------

human          AGCCTCACCAATCCCTCAGGAGGAAAAACAAGCTCTGCGTCATCTGTCACATCTCCTTCA
rabbit         ------------------------------------------------------------

human          ATAGGGCTTGAGACTCTGAGGGCCAATGTAAGTGCAGTGAAAAGTGACATTGCCCCTACT
rabbit         ------------------------------------------------------------
```

```
human           GCTGGGCATCTATCTCAGACTTCATCTCCTGCGGAAGTGAGCATCCTGGACGTAACCACA
rabbit          ------------------------------------------------------------

human           GCTCCTACTCCAGGTATCTCCACCACCATCACCACCATGGGAACCAACTCAATCTCAACT
rabbit          ------------------------------------------------------------

human           ACCACACCCAACCCAGAAGTGGGTATGAGTACCATGGACAGCACCCCGGCCACAGAGAGG
rabbit          ------------------------------------------------------------

human           CGCACAACTTCTACAGAACACCCTTCCACCTGGTCTTCCACAGCTGCATCAGATTCCTGG
rabbit          ------------------------------------------------------------

human           ACTGTCACAGACATGACTTCAAACTTGAAAGTTGCAAGATCTCCTGGAACAATTTCCACA
rabbit          ------------------------------------------------------------

human           ATGCATACAACTTCATTCTTAGCCTCAAGCACTGAATTAGACTCCATGTCTACTCCCCAT
rabbit          ------------------------------------------------------------

human           GGCCGTATAACTGTCATTGGAACCAGCCTGGTCACTCCATCCTCTGATGCTTCAGCTGTA
rabbit          ------------------------------------------------------------

human           AAGACAGAGACCAGTACAAGTGAAAGAACATTGAGTCCTTCAGACACAACTGCATCTACT
rabbit          ------------------------------------------------------------

human           CCCATCTCAACTTTTTCTCGTGTCCAGAGGATGAGCATCTCAGTTCCTGACATTTTAAGT
rabbit          ------------------------------------------------------------
```

```
human     ACAAGTTGGACTCCCAGTAGTACAGAAGCAGAAGATGTGCCTGTTTCAATGGTTTCTACA
rabbit    ------------------------------------------------------------

human     GATCATGCTAGTACAAAGACTGACCCAAATACGCCCCTGTCCACTTTTCTGTTTGATTCT
rabbit    ------------------------------------------------------------

human     CTGTCCACTCTTGACTGGGACACTGGGAGATCTCTGTCATCAGCCACAGCCACTACCTCA
rabbit    ------------------------------------------------------------

human     GCTCCTCAGGGGGCCACAACTCCCCAGGAACTCACTTTGGAAACCATGATCAGCCCAGCT
rabbit    ------------------------------------------------------------

human     ACCTCACAGTTGCCCTTCTCTATAGGGCACATTACAAGTGCAGTCACACCAGCTGCAATG
rabbit    ------------------------------------------------------------

human     GCAAGGAGCTCTGGAGTTACTTTTTCAAGACCAGATCCCACAAGCAAAAAGGCAGAGCAG
rabbit    ------------------------------------------------------------

human     ACTTCCACTCAGCTTCCCACCACCACTTCTGCACATCCAGGGCAGGTGCCCAGATCAGCA
rabbit    ------------------------------------------------------------

human     GCAACAACTCTGGATGTGATCCCACACACAGCAAAAACTCCAGATGCAACTTTTCAGAGA
rabbit    ------------------------------------------------------------

human     CAAGGGCAGACAGCTCTTACAACAGAGGCAAGAGCTACATCTGACTCCTGGAATGAGAAA
rabbit    ------------------------------------------------------------
```

```
human           GAAAAATCAACCCCAAGTGCACCTTGGATCACTGAGATGATGAATTCTGTCTCAGAAGAT
rabbit          ------------------------------------------------------------

human           ACCATCAAGGAGGTTACCAGCTCCTCCAGTGTATTAAGGACCCTGAATACGCTGGACATA
rabbit          ------------------------------------------------------------

human           AACTTGGAATCTGGGACGACTTCATCCCCAAGTTGGAAAAGCAGCCCATATGAGAGAATT
rabbit          ------------------------------------------------------------

human           GCCCCTTCTGAGTCCACCACAGACAAAGAGGCAATTCACCCTTCTACAAACACAGTAGAG
rabbit          ------------------------------------------------------------

human           ACCACAGGCTGGGTCACAAGTTCCGAACATGCTTCTCATTCCACTATCCCAGCCCACTCA
rabbit          ------------------------------------------------------------

human           GCGTCATCCAAACTCACATCTCCAGTGGTTACAACCTCCACCAGGGAACAAGCAATAGTT
rabbit          ------------------------------------------------------------

human           TCTATGTCAACAACCACATGGCCAGAGTCTACAAGGGCTAGAACAGAGCCTAATTCCTTC
rabbit          ------------------------------------------------------------

human           TTGACTATTGAACTGAGGGACGTCAGCCCTTACATGGACACCAGCTCAACCACACAAACA
rabbit          ------------------------------------------------------------

human           AGTATTATCTCTTCCCCAGGTTCCACTGCGATCACCAAGGGGCCTAGAACAGAAATTACC
rabbit          ------------------------------------------------------------
```

```
human           TCCTCTAAGAGAATATCCAGCTCATTCCTTGCCCAGTCTATGAGGTCGTCAGACAGCCCC
rabbit          ------------------------------------------------------------

human           TCAGAAGCCATCACCAGGCTGTCTAACTTTCCTGCCATGACAGAATCTGGAGGAATGATC
rabbit          ------------------------------------------------------------

human           CTTGCTATGCAAACAAGTCCACCTGGCGCTACATCACTAAGTGCACCTACTTTGGATACA
rabbit          ------------------------------------------------------------

human           TCAGCCACAGCCTCCTGGACAGGGACTCCACTGGCTACGACTCAGAGATTTACATACTCA
rabbit          ------------------------------------------------------------

human           GAGAAGACCACTCTCTTTAGCAAAGGTCCTGAGGATACATCACAGCCAAGCCCTCCCTCT
rabbit          ------------------------------------------------------------

human           GTGGAAGAAACCAGCTCTTCCTCTTCCCTGGTACCTATCCATGCTACAACCTCGCCTTCC
rabbit          ------------------------------------------------------------

human           AATATTTTGTTGACATCACAAGGGCACAGTCCCTCCTCTACTCCACCTGTGACCTCAGTT
rabbit          ------------------------------------------------------------

human           TTCTTGTCTGAGACCTCTGGCCTGGGGAAGACCACAGACATGTCGAGGATAAGCTTGGAA
rabbit          ------------------------------------------------------------

human           CCTGGCACAAGTTTACCTCCCAATTTGAGCAGTACAGCAGGTGAGGCGTTATCCACTTAT
rabbit          ------------------------------------------------------------
```

```
human        GAAGCCTCCAGAGATACAAAGGCAATTCATCATTCTGCAGACACAGCAGTGACGAATATG
rabbit       ------------------------------------------------------------

human        GAGGCAACCAGTTCTGAATATTCTCCTATCCCAGGCCATACAAAGCCATCCAAAGCCACA
rabbit       ------------------------------------------------------------

human        TCTCCATTGGTTACCTCCCACATCATGGGGGACATCACTTCTTCCACATCAGTATTTGGC
rabbit       ------------------------------------------------------------

human        TCCTCCGAGACCACAGAGATTGAGACAGTGTCCTCTGTGAACCAGGGACTTCAGGAGAGA
rabbit       ------------------------------------------------------------

human        AGCACATCCCAGGTGGCCAGCTCTGCTACAGAGACAAGCACTGTCATTACCCATGTGTCT
rabbit       ------------------------------------------------------------

human        AGTGGTGATGCTACTACTCATGTCACCAAGACACAAGCCACTTTCTCTAGCGGAACATCC
rabbit       ------------------------------------------------------------

human        ATCTCAAGCCCTCATCAGTTTATAACTTCTACCAACACATTTACAGATGTGAGCACCAAC
rabbit       ------------------------------------------------------------

human        CCCTCCACCTCTCTGATAATGACAGAATCTTCAGGAGTGACCATCACCACCCAAACAGGT
rabbit       ------------------------------------------------------------

human        CCTACTGGAGCTGCAACACAGGGTCCATATCTCTTGGACACATCAACCATGCCTTACTTG
rabbit       ------------------------------------------------------------
```

```
human           ACAGAGACTCCATTAGCTGTGACTCCAGATTTTATGCAATCAGAGAAGACCACTCTCATA
rabbit          ------------------------------------------------------------

human           AGCAAAGGTCCCAAGGATGTGTCCTGGACAAGCCCTCCCTCTGTGGCAGAAACCAGCTAT
rabbit          ------------------------------------------------------------

human           CCCTCTTCCCTGACACCTTTCTTGGTCACAACCATACCTCCTGCCACTTCCACGTTACAA
rabbit          ------------------------------------------------------------

human           GGGCAACATACATCCTCTCCTGTTTCTGCGACTTCAGTTCTTACCTCTGGACTGGTGAAG
rabbit          ------------------------------------------------------------

human           ACCACAGATATGTTGAACACAAGCATGGAACCTGTGACCAATTCACCTCAAAATTTGAAC
rabbit          ------------------------------------------------------------

human           AATCCATCAAATGAGATACTGGCCACTTTGGCAGCCACCACAGATATAGAGACTATTCAT
rabbit          ------------------------------------------------------------

human           CCTTCCATAAACAAAGCAGTGACCAATATGGGGACTGCCAGTTCAGCACATGTACTGCAT
rabbit          ------------------------------------------------------------

human           TCCACTCTCCCAGTCAGCTCAGAACCATCTACAGCCACATCTCCAATGGTTCCTGCCTCC
rabbit          ------------------------------------------------------------

human           AGCATGGGGGACGCTCTTGCTTCTATATCAATACCTGGTTCTGAGACCACAGACATTGAG
rabbit          ------------------------------------------------------------
```

```
human     GGAGAGCCAACATCCTCCCTGACTGCTGGACGAAAAGAGAACAGCACCCTCCAGGAGATG
rabbit    ------------------------------------------------------------

human     AACTCAACTACAGAGTCAAACATCATCCTCTCCAATGTGTCTGTGGGGGCTATTACTGAA
rabbit    ------------------------------------------------------------

human     GCCACAAAAATGGAAGTCCCCTCTTTTGATGCAACATTCATACCAACTCCTGCTCAGTCA
rabbit    ------------------------------------------------------------

human     ACAAAGTTCCCAGATATTTTCTCAGTAGCCAGCAGTAGACTTTCAAACTCTCCTCCCATG
rabbit    ------------------------------------------------------------

human     ACAATATCTACCCACATGACCACCACCCAGACAGGGTCTTCTGGAGCTACATCAAAGATT
rabbit    ------------------------------------------------------------

human     CCACTTGCCTTAGACACATCAACCTTGGAAACCTCAGCAGGGACTCCATCAGTGGTGACT
rabbit    ------------------------------------------------------------

human     GAGGGGTTTGCCCACTCAAAAATAACCACTGCAATGAACAATGATGTCAAGGACGTGTCA
rabbit    ------------------------------------------------------------

human     CAGACAAACCCTCCCTTTCAGGATGAAGCCAGCTCTCCCTCTTCTCAAGCACCTGTCCTT
rabbit    ------------------------------------------------------------

human     GTCACAACCTTACCTTCTTCTGTTGCTTTCACACCGCAATGGCACAGTACCTCCTCTCCT
rabbit    ------------------------------------------------------------
```

```
human     GTTTCTATGTCCTCAGTTCTTACTTCTTCACTGGTAAAGACCGCAGGCAAGGTGGATACA
rabbit    ------------------------------------------------------------

human     AGCTTAGAAACAGTGACCAGTTCACCTCAAAGTATGAGCAACACTTTGGATGACATATCG
rabbit    ------------------------------------------------------------

human     GTCACTTCAGCAGCCACCACAGATATAGAGACAACGCATCCTTCCATAAACACAGTAGTT
rabbit    ------------------------------------------------------------

human     ACCAATGTGGGGACCACCGGTTCAGCATTTGAATCACATTCTACTGTCTCAGCTTACCCA
rabbit    ------------------------------------------------------------

human     GAGCCATCTAAAGTCACATCTCCAAATGTTACCACCTCCACCATGGAAGACACCACAATT
rabbit    ------------------------------------------------------------

human     TCCAGATCAATACCTAAATCCTCTAAGACTACAAGAACTGAGACTGAGACAACTTCCTCC
rabbit    ------------------------------------------------------------

human     CTGACTCCTAAACTGAGGGAGACCAGCATCTCCCAGGAGATCACCTCGTCCACAGAGACA
rabbit    ------------------------------------------------------------

human     AGCACTGTTCCTTACAAAGAGCTCACTGGTGCCACTACCGAGGTATCCAGGACAGATGTC
rabbit    ------------------------------------------------------------

human     ACTTCCTCTAGCAGTACATCCTTCCCTGGCCCTGATCAGTCCACAGTGTCACTAGACATC
rabbit    ------------------------------------------------------------
```

```
human       TCCACAGAAACCAACACCAGGCTGTCTACCTCCCCAATAATGACAGAATCTGCAGAAATA
rabbit      ------------------------------------------------------------

human       ACCATCACCACCCAAACAGGTCCTCATGGGGCTACATCACAGGATACTTTTACCATGGAC
rabbit      ------------------------------------------------------------

human       CCATCAAATACAACCCCCCAGGCAGGGATCCACTCAGCTATGACTCATGGATTTTCACAA
rabbit      ------------------------------------------------------------

human       TTGGATGTGACCACTCTTATGAGCAGAATTCCACAGGATGTATCATGGACAAGTCCTCCC
rabbit      ------------------------------------------------------------

human       TCTGTGGATAAAACCAGCTCCCCCTCTTCCTTTCTGTCCTCACCTGCAATGACCACACCT
rabbit      ------------------------------------------------------------

human       TCCCTGATTTCTTCTACCTTACCAGAGGATAAGCTCTCCTCTCCTATGACTTCACTTCTC
rabbit      ------------------------------------------------------------

human       ACCTCTGGCCTAGTGAAGATTACAGACATATTACGTACACGCTTGGAACCTGTGACCAGC
rabbit      ------------------------------------------------------------

human       TCACTTCCAAATTTCAGCAGCACCTCAGATAAGATACTGGCCACTTCTAAAGACAGTAAA
rabbit      ------------------------------------------------------------

human       GACACAAAGGAAATTTTTCCTTCTATAAACACAGAAGAGACCAATGTGAAAGCCAACAAC
rabbit      ------------------------------------------------------------
```

```
human           TCTGGACATGAATCCCATTCCCCTGCACTGGCTGACTCAGAGACACCCAAAGCCACAACT
rabbit          ------------------------------------------------------------

human           CAAATGGTTATCACCACCACTGTGGGAGATCCAGCTCCTTCCACATCAATGCCAGTGCAT
rabbit          ------------------------------------------------------------

human           GGTTCCTCTGAGACTACAAACATTAAGAGAGAGCCAACATATTTCTTGACTCCTAGACTG
rabbit          ------------------------------------------------------------

human           AGAGAGACCAGTACCTCTCAGGAGTCCAGCTTTCCCACGGACACAAGTTTTCTACTTTCC
rabbit          ------------------------------------------------------------

human           AAAGTCCCCACTGGTACTATTACTGAGGTCTCCAGTACAGGGGTCAACTCTTCTAGCAAA
rabbit          ------------------------------------------------------------

human           ATTTCCACCCCAGACCATGATAAGTCCACAGTGCCACCTGACACCTTCACAGGAGAGATC
rabbit          ------------------------------------------------------------

human           CCCAGGGTCTTCACCTCCTCTATTAAGACAAAATCTGCAGAAATGACGATCACCACCCAA
rabbit          ------------------------------------------------------------

human           GCAAGTCCTCCTGAGTCTGCATCGCACAGTACCCTTCCCTTGGACACATCAACCACACTT
rabbit          ------------------------------------------------------------

human           TCCCAGGGAGGGACTCATTCAACTGTGACTCAGGGATTCCCATACTCAGAGGTGACCACT
rabbit          ------------------------------------------------------------
```

```
human          CTCATGGGCATGGGTCCTGGGAATGTGTCATGGATGACAACTCCCCCTGTGGAAGAAACC
rabbit         ------------------------------------------------------------

human          AGCTCTGTGTCTTCCCTGATGTCTTCACCTGCCATGACATCCCCTTCTCCTGTTTCCTCC
rabbit         ------------------------------------------------------------

human          ACATCACCACAGAGCATCCCCTCCTCTCCTCTTCCTGTGACTGCACTTCCTACTTCTGTT
rabbit         ------------------------------------------------------------

human          CTGGTGACAACCACAGATGTGTTGGGCACAACAAGCCCAGAGTCTGTAACCAGTTCACCT
rabbit         ------------------------------------------------------------

human          CCAAATTTGAGCAGCATCACTCATGAGAGACCGGCCACTTACAAAGACACTGCACACACA
rabbit         ------------------------------------------------------------

human          GAAGCCGCCATGCATCATTCCACAAACACCGCAGTGACCAATGTAGGGACTTCCGGGTCT
rabbit         ------------------------------------------------------------

human          GGACATAAATCACAATCCTCTGTCCTAGCTGACTCAGAGACATCGAAAGCCACACCTCTG
rabbit         ------------------------------------------------------------

human          ATGAGTACCACCTCCACCCTGGGGGACACAAGTGTTTCCACATCAACTCCTAATATCTCT
rabbit         ------------------------------------------------------------

human          CAGACTAACCAAATTCAAACAGAGCCAACAGCATCCCTGAGCCCTAGACTGAGGGAGAGC
rabbit         ------------------------------------------------------------
```

```
human               AGCACGTCTGAGAAGACCAGCTCAACAACAGAGACAAATACTGCCTTTTCTTATGTGCCC
rabbit              ------------------------------------------------------------

human               ACAGGTGCTATTACTCAGGCCTCCAGAACAGAAATCTCCTCTAGCAGAACATCCATCTCA
rabbit              ------------------------------------------------------------

human               GACCTTGATCGGCCCACAATAGCACCCGACATCTCCACAGGAATGATCACCAGGCTCTTC
rabbit              ------------------------------------------------------------

human               ACCTCCCCCATCATGACAAAATCTGCAGAAATGACCGTCACCACTCAAACAACTACTCCT
rabbit              ------------------------------------------------------------

human               GGGGCTACATCACAGGGTATCCTTCCCTGGGACACATCAACCACACTTTTCCAGGGAGGG
rabbit              ------------------------------------------------------------

human               ACTCATTCAACCGTGTCTCAGGGATTCCCACACTCAGAGATAACCACTCTTCGGAGCAGA
rabbit              ------------------------------------------------------------

human               ACCCCTGGAGATGTGTCATGGATGACAACTCCCCCTGTGGAAGAAACCAGCTCTGGGTTT
rabbit              ------------------------------------------------------------

human               TCCCTGATGTCACCTTCCATGACATCCCCTTCTCCTGTTTCCTCCACATCACCAGAGAGC
rabbit              ------------------------------------------------------------

human               ATCCCCTCCTCTCCTCTCCCTGTGACTGCACTTCTTACTTCTGTTCTGGTGACAACCACA
rabbit              ------------------------------------------------------------
```

```
human      AATGTATTGGGCACAACAAGCCCAGAGCCCGTAACGAGTTCACCTCCAAATTTAAGCAGC
rabbit     ------------------------------------------------------------

human      CCCACACAGGAGAGACTGACCACTTACAAAGACACTGCGCACACAGAAGCCATGCATGCT
rabbit     ------------------------------------------------------------

human      TCCATGCATACAAACACTGCAGTGGCCAACGTGGGGACCTCCATTTCTGGACATGAATCA
rabbit     ------------------------------------------------------------

human      CAATCTTCTGTCCCAGCTGATTCACACACATCCAAAGCCACATCTCCAATGGGTATCACC
rabbit     ------------------------------------------------------------

human      TTCGCCATGGGGGATACAAGTGTTTCTACATCAACTCCTGCCTTCTTTGAGACTAGAATT
rabbit     ------------------------------------------------------------

human      CAGACTGAATCAACATCCTCTTTGATTCCTGGATTAAGGGACACCAGGACGTCTGAGGAG
rabbit     ------------------------------------------------------------

human      ATCAACACTGTGACAGAGACCAGCACTGTCCTTTCAGAAGTGCCCACTACTACTACTACT
rabbit     ------------------------------------------------------------

human      GAGGTCTCCAGGACAGAAGTTATCACTTCCAGCAGAACAACCATCTCAGGGCCTGATCAT
rabbit     ------------------------------------------------------------

human      TCCAAAATGTCACCCTACATCTCCACAGAAACCATCACCAGGCTCTCCACTTTTCCTTTT
rabbit     ------------------------------------------------------------
```

```
human     GTAACAGGATCCACAGAAATGGCCATCACCAACCAAACAGGTCCTATAGGGACTATCTCA
rabbit    ------------------------------------------------------------

human     CAGGCTACCCTTACCCTGGACACATCAAGCACAGCTTCCTGGGAAGGGACTCACTCACCT
rabbit    ------------------------------------------------------------

human     GTGACTCAGAGATTTCCACACTCAGAGGAGACCACTACTATGAGCAGAAGTACTAAGGGC
rabbit    ------------------------------------------------------------

human     GTGTCATGGCAAAGCCCTCCCTCTGTGGAAGAAACCAGTTCTCCTTCTTCCCCAGTGCCT
rabbit    ------------------------------------------------------------

human     TTACCTGCAATAACCTCACATTCATCTCTTTATTCCGCAGTATCAGGAAGTAGCCCCACT
rabbit    ------------------------------------------------------------

human     TCTGCTCTCCCTGTGACTTCCCTTCTCACCTCTGGCAGGAGGAAGACCATAGACATGTTG
rabbit    ------------------------------------------------------------

human     GACACACACTCAGAACTTGTGACCAGCTCCTTACCAAGTGCAAGTAGCTTCTCAGGTGAG
rabbit    ------------------------------------------------------------

human     ATACTCACTTCTGAAGCCTCCACAAATACAGAGACAATTCACTTTTCAGAGAACACAGCA
rabbit    ------------------------------------------------------------

human     GAAACCAATATGGGGACCACCAATTCTATGCATAAACTACATTCCTCTGTCTCAATCCAC
rabbit    ------------------------------------------------------------
```

```
human     TCCCAGCCATCCGGACACACACCTCCAAAGGTTACTGGATCTATGATGGAGGACGCTATT
rabbit    ------------------------------------------------------------

human     GTTTCCACATCAACACCTGGTTCTCCTGAGACTAAAAATGTTGACAGAGACTCAACATCC
rabbit    ------------------------------------------------------------

human     CCTCTGACTCCTGAACTGAAAGAGGACAGCACCGCCCTGGTGATGAACTCAACTACAGAG
rabbit    ------------------------------------------------------------

human     TCAAACACTGTTTTCTCCAGTGTGTCCCTGGATGCTGCTACTGAGGTCTCCAGGGCAGAA
rabbit    ------------------------------------------------------------

human     GTCACCTACTATGATCCTACATTCATGCCAGCTTCTGCTCAGTCAACAAAGTCCCCAGAC
rabbit    ------------------------------------------------------------

human     ATTTCACCTGAAGCCAGCAGCAGTCATTCTAACTCTCCTCCCTTGACAATATCTACACAC
rabbit    ------------------------------------------------------------

human     AAGACCATCGCCACACAAACAGGTCCTTCTGGGGTGACATCTCTTGGCCAACTGACCCTG
rabbit    ------------------------------------------------------------

human     GACACATCAACCATAGCCACCTCAGCAGGAACTCCATCAGCCAGAACTCAGGATTTTGTA
rabbit    ------------------------------------------------------------

human     GATTCAGAAACAACCAGTGTCATGAACAATGATCTCAATGATGTGTTGAAGACAAGCCCT
rabbit    ------------------------------------------------------------
```

```
human     TTCTCTGCAGAAGAAGCCAACTCTCTCTCTTCTCAGGCACCTCTCCTTGTGACAACCTCA
rabbit    ------------------------------------------------------------

human     CCTTCTCCTGTAACTTCCACATTGCAAGAGCACAGTACCTCCTCTCTTGTTTCTGTGACC
rabbit    ------------------------------------------------------------

human     TCAGTACCCACCCCTACACTGGCGAAGATCACAGACATGGACACAAACTTAGAACCTGTG
rabbit    ------------------------------------------------------------

human     ACTCGTTCACCTCAAAATTTAAGGAACACCTTGGCCACTTCAGAAGCCACCACAGATACA
rabbit    ------------------------------------------------------------

human     CACACAATGCATCCTTCTATAAACACAGCAGTGGCCAATGTGGGGACCACCAGTTCACCA
rabbit    ------------------------------------------------------------

human     AATGAATTCTATTTTACTGTCTCACCTGACTCAGACCCATATAAAGCCACATCCGCAGTA
rabbit    ------------------------------------------------------------

human     GTTATCACTTCCACCTCGGGGGACTCAATAGTTTCCACATCAATGCCTAGATCCTCTGCG
rabbit    ------------------------------------------------------------

human     ATGAAAAAGATTGAGTCTGAGACAACTTTCTCCCTGATATTTAGACTGAGGGAGACTAGC
rabbit    ------------------------------------------------------------

human     ACCTCCCAGAAAATTGGCTCATCCTCAGACACAAGCACGGTCTTTGACAAAGCATTCACT
rabbit    ------------------------------------------------------------
```

```
human           GCTGCTACTACTGAGGTCTCCAGAACAGAACTCACCTCCTCTAGCAGAACATCCATCCAA
rabbit          ------------------------------------------------------------

human           GGCACTGAAAAGCCCACAATGTCACCGGACACCTCCACAAGATCTGTCACCATGCTTTCT
rabbit          ------------------------------------------------------------

human           ACTTTTGCTGGCCTGACAAAATCCGAAGAAAGGACCATTGCCACCCAAACAGGTCCTCAT
rabbit          ------------------------------------------------------------

human           AGGGCGACATCACAGGGTACCCTTACCTGGGACACATCAATCACAACCTCACAGGCAGGG
rabbit          ------------------------------------------------------------

human           ACCCACTCAGCTATGACTCATGGATTTTCACAATTAGATTTGTCCACTCTTACGAGTAGA
rabbit          ------------------------------------------------------------

human           GTTCCTGAGTACATATCAGGGACAAGCCCACCCTCTGTGGAAAAAACCAGCTCTTCCTCT
rabbit          ------------------------------------------------------------

human           TCCCTTCTGTCTTTACCAGCAATAACCTCACCGTCCCCTGTACCTACTACATTACCAGAA
rabbit          ------------------------------------------------------------

human           AGTAGGCCGTCTTCTCCTGTTCATCTGACTTCACTCCCCACCTCTGGCCTAGTGAAGACC
rabbit          ------------------------------------------------------------

human           ACAGATATGCTGGCATCTGTGGCCAGTTTACCTCCAAACTTGGGCAGCACCTCACATAAG
rabbit          ------------------------------------------------------------
```

```
human     ATACCGACTACTTCAGAAGACATTAAAGATACAGAGAAAATGTATCCTTCCACAAACATA
rabbit    ------------------------------------------------------------

human     GCAGTAACCAATGTGGGGACCACCACTTCTGAAAAGGAATCTTATTCGTCTGTCCCAGCC
rabbit    ------------------------------------------------------------

human     TACTCAGAACCACCCAAAGTCACCTCTCCAATGGTTACCTCTTTCAACATAAGGGACACC
rabbit    ------------------------------------------------------------

human     ATTGTTTCCACATCCATGCCTGGCTCCTCTGAGATTACAAGGATTGAGATGGAGTCAACA
rabbit    ------------------------------------------------------------

human     TTCTCCCTGGCTCATGGGCTGAAGGGAACCAGCACCTCCCAGGACCCCATCGTATCCACA
rabbit    ------------------------------------------------------------

human     GAGAAAAGTGCTGTCCTTCACAAGTTGACCACTGGTGCTACTGAGACCTCTAGGACAGAA
rabbit    ------------------------------------------------------------

human     GTTGCCTCTTCTAGAAGAACATCCATTCCAGGCCCTGATCATTCCACAGAGTCACCAGAC
rabbit    ------------------------------------------------------------

human     ATCTCCACTGAAGTGATCCCCAGCCTGCCTATCTCCCTTGGCATTACAGAATCTTCAAAT
rabbit    ------------------------------------------------------------

human     ATGACCATCATCACTCGAACAGGTCCTCCTCTTGGCTCTACATCACAGGGCACATTTACC
rabbit    ------------------------------------------------------------
```

```
human     TTGGACACACCAACTACATCCTCCAGGGCAGGAACACACTCGATGGCGACTCAGGAATTT
rabbit    ------------------------------------------------------------

human     CCACACTCAGAAATGACCACTGTCATGAACAAGGACCCTGAGATTCTATCATGGACAATC
rabbit    ------------------------------------------------------------

human     CCTCCTTCTATAGAGAAAACCAGCTTCTCCTCTTCCCTGATGCCTTCACCAGCCATGACT
rabbit    ------------------------------------------------------------

human     TCACCTCCTGTTTCCTCAACATTACCAAAGACCATTCACACCACTCCTTCTCCTATGACC
rabbit    ------------------------------------------------------------

human     TCACTGCTCACCCCTAGCCTAGTGATGACCACAGACACATTGGGCACAAGCCCAGAACCT
rabbit    ------------------------------------------------------------

human     ACAACCAGTTCACCTCCAAATTTGAGCAGTACCTCACATGAGATACTGACAACAGATGAA
rabbit    ------------------------------------------------------------

human     GACACCACAGCTATAGAAGCCATGCATCCTTCCACAAGCACAGCAGCGACTAATGTGGAA
rabbit    ------------------------------------------------------------

human     ACCACCAGTTCTGGACATGGGTCACAATCCTCTGTCCTAGCTGACTCAGAAAAAACCAAG
rabbit    ------------------------------------------------------------

human     GCCACAGCTCCAATGGATACCACCTCCACCATGGGGCATACAACTGTTTCCACATCAATG
rabbit    ------------------------------------------------------------
```

```
human     TCTGTTTCCTCTGAGACTACAAAAATTAAGAGAGAGTCAACATATTCCTTGACTCCTGGA
rabbit    ------------------------------------------------------------

human     CTGAGAGAGACCAGCATTTCCCAAAATGCCAGCTTTTCCACTGACACAAGTATTGTTCTT
rabbit    ------------------------------------------------------------

human     TCAGAAGTCCCCACTGGTACTACTGCTGAGGTCTCCAGGACAGAAGTCACCTCCTCTGGT
rabbit    ------------------------------------------------------------

human     AGAACATCCATCCCTGGCCCTTCTCAGTCCACAGTTTTGCCAGAAATATCCACAAGAACA
rabbit    ------------------------------------------------------------

human     ATGACAAGGCTCTTTGCCTCGCCCACCATGACAGAATCAGCAGAAATGACCATCCCCACT
rabbit    ------------------------------------------------------------

human     CAAACAGGTCCTTCTGGGTCTACCTCACAGGATACCCTTACCTTGGACACATCCACCACA
rabbit    ------------------------------------------------------------

human     AAGTCCCAGGCAAAGACTCATTCAACTTTGACTCAGAGATTTCCACACTCAGAGATGACC
rabbit    ------------------------------------------------------------

human     ACTCTCATGAGCAGAGGTCCTGGAGATATGTCATGGCAAAGCTCTCCCTCTCTGGAAAAT
rabbit    ------------------------------------------------------------

human     CCCAGCTCTCTCCCTTCCCTGCTGTCTTTACCTGCCACAACCTCACCTCCTCCCATTTCC
rabbit    ------------------------------------------------------------
```

```
human     TCCACATTACCAGTGACTATCTCCTCCTCTCCTCTTCCTGTGACTTCACTTCTCACCTCT
rabbit    ------------------------------------------------------------

human     AGCCCGGTAACGACCACAGACATGTTACACACAAGCCCAGAACTTGTAACCAGTTCACCT
rabbit    ------------------------------------------------------------

human     CCAAAGCTGAGCCACACTTCAGATGAGAGACTGACCACTGGCAAGGACACCACAAATACA
rabbit    ------------------------------------------------------------

human     GAAGCTGTGCATCCTTCCACAAACACAGCAGCGTCCAATGTGGAGATTCCCAGCTCTGGA
rabbit    ------------------------------------------------------------

human     CATGAATCCCCTTCCTCTGCCTTAGCTGACTCAGAGACATCCAAAGCCACATCACCAATG
rabbit    ------------------------------------------------------------

human     TTTATTACCTCCACCCAGGAGGATACAACTGTTGCCATATCAACCCCTCACTTCTTGGAG
rabbit    ------------------------------------------------------------

human     ACTAGCAGAATTCAGAAAGAGTCAATTTCCTCCCTGAGCCCTAAATTGAGGGAGACAGGC
rabbit    ------------------------------------------------------------

human     AGTTCTGTGGAGACAAGCTCAGCCATAGAGACAAGTGCTGTCCTTTCTGAAGTGTCCATT
rabbit    ------------------------------------------------------------

human     GGTGCTACTACTGAGATCTCCAGGACAGAAGTCACCTCCTCTAGCAGAACATCCATCTCT
rabbit    ------------------------------------------------------------
```

```
human    GGTTCTGCTGAGTCCACAATGTTGCCAGAAATATCCACCACAAGAAAAATCATTAAGTTC
rabbit   ------------------------------------------------------------

human    CCTACTTCCCCCATCCTGGCAGAATCATCAGAAATGACCATCAAGACCCAAACAAGTCCT
rabbit   ------------------------------------------------------------

human    CCTGGGTCTACATCAGAGAGTACCTTTACATTAGACACATCAACCACTCCCTCCTTGGTA
rabbit   ------------------------------------------------------------

human    ATAACCCATTCGACTATGACTCAGAGATTGCCACACTCAGAGATAACCACTCTTGTGAGT
rabbit   ------------------------------------------------------------

human    AGAGGTGCTGGGGATGTGCCACGGCCCAGCTCTCTCCCTGTGGAAGAAACAAGCCCTCCA
rabbit   ------------------------------------------------------------

human    TCTTCCCAGCTGTCTTTATCTGCCATGATCTCACCTTCTCCTGTTTCTTCCACATTACCA
rabbit   ------------------------------------------------------------

human    GCAAGTAGCCACTCCTCTTCTGCTTCTGTGACTTCACTTCTCACACCAGGCCAAGTGAAG
rabbit   ------------------------------------------------------------

human    ACTACTGAGGTGTTGGACGCAAGTGCAGAACCTGAAACCAGTTCACCTCCAAGTTTGAGC
rabbit   ------------------------------------------------------------

human    AGCACCTCAGTTGAAATACTGGCCACCTCTGAAGTCACCACAGATACGGAGAAAATTCAT
rabbit   ------------------------------------------------------------
```

```
human          CCTTTCTCAAACACGGCAGTAACCAAAGTTGGAACTTCCAGTTCTGGACATGAATCCCCT
rabbit         ------------------------------------------------------------

human          TCCTCTGTCCTACCTGACTCAGAGACAACCAAAGCCACATCGGCAATGGGTACCATCTCC
rabbit         ------------------------------------------------------------

human          ATTATGGGGGATACAAGTGTTTCTACATTAACTCCTGCCTTATCTAACACTAGGAAAATT
rabbit         ------------------------------------------------------------

human          CAGTCAGAGCCAGCTTCCTCACTGACCACCAGATTGAGGGAGACCAGCACCTCTGAAGAG
rabbit         ------------------------------------------------------------

human          ACCAGCTTAGCCACAGAAGCAAACACTGTTCTTTCTAAAGTGTCCACTGGTGCTACTACT
rabbit         ------------------------------------------------------------

human          GAGGTCTCCAGGACAGAAGCCATCTCCTTTAGCAGAACATCCATGTCAGGCCCTGAGCAG
rabbit         ------------------------------------------------------------

human          TCCACAATGTCACAAGACATCTCCATAGGAACCATCCCCAGGATTTCTGCCTCCTCTGTC
rabbit         ------------------------------------------------------------

human          CTGACAGAATCTGCAAAAATGACCATCACAACCCAAACAGGTCCTTCGGAGTCTACACTA
rabbit         ------------------------------------------------------------

human          GAAAGTACCCTTAATTTGAACACAGCAACCACACCCTCTTGGGTGGAAACCCACTCTATA
rabbit         ------------------------------------------------------------
```

```
human     GTAATTCAGGGATTTCCACACCCAGAGATGACCACTTCCATGGGCAGAGGTCCTGGAGGT
rabbit    ------------------------------------------------------------

human     GTGTCATGGCCTAGCCCTCCCTTTGTGAAAGAAACCAGCCCTCCATCCTCCCCGCTGTCT
rabbit    ------------------------------------------------------------

human     TTACCTGCCGTGACCTCACCTCATCCTGTTTCCACCACATTCCTAGCACATATCCCCCCC
rabbit    ------------------------------------------------------------

human     TCTCCCCTTCCTGTGACTTCACTTCTCACCTCTGGCCCGGCGACAACCACAGATATCTTG
rabbit    ------------------------------------------------------------

human     GGTACAAGCACAGAACCTGGAACCAGTTCATCTTCAAGTTTGAGCACCACCTCCCATGAG
rabbit    ------------------------------------------------------------

human     AGACTGACCACTTACAAAGACACTGCACATACAGAAGCCGTGCATCCTTCCACAAACACA
rabbit    ------------------------------------------------------------

human     GGAGGGACCAATGTGGCAACCACCAGCTCTGGATATAAATCACAGTCCTCTGTCCTAGCT
rabbit    ------------------------------------------------------------

human     GACTCATCTCCAATGTGTACCACCTCCACCATGGGGGATACAAGTGTTCTCACATCAACT
rabbit    ------------------------------------------------------------

human     CCTGCCTTCCTTGAGACTAGGAGGATTCAGACAGAGCTAGCTTCCTCCCTGACCCCTGGA
rabbit    ------------------------------------------------------------
```

```
human           TTGAGGGAGTCCAGCGGCTCTGAAGGGACCAGCTCAGGCACCAAGATGAGCACTGTCCTC
rabbit          ------------------------------------------------------------

human           TCTAAAGTGCCCACTGGTGCTACTACTGAGATCTCCAAGGAAGACGTCACCTCCATCCCA
rabbit          ------------------------------------------------------------

human           GGTCCCGCTCAATCCACAATATCACCAGACATCTCCACAAGAACCGTCAGCTGGTTCTCT
rabbit          ------------------------------------------------------------

human           ACATCCCCTGTCATGACAGAATCAGCAGAAATAACCATGAACACCCATACAAGTCCTTTA
rabbit          ------------------------------------------------------------

human           GGGGCCACAACACAAGGCACCAGTACTTTGGACACGTCAAGCACAACCTCTTTGACAATG
rabbit          ------------------------------------------------------------

human           ACACACTCAACTATATCTCAAGGATTTTCACACTCACAGATGAGCACTCTTATGAGGAGG
rabbit          ------------------------------------------------------------

human           GGTCCTGAGGATGTATCATGGATGAGCCCTCCCCTTCTGGAAAAAACTAGACCTTCCTTT
rabbit          ------------------------------------------------------------

human           TCTCTGATGTCTTCACCAGCCACAACTTCACCTTCTCCTGTTTCCTCCACATTACCAGAG
rabbit          ------------------------------------------------------------

human           AGCATCTCTTCCTCTCCTCTTCCTGTGACTTCACTCCTCACGTCTGGCTTGGCAAAAACT
rabbit          ------------------------------------------------------------
```

```
human     ACAGATATGTTGCACAAAAGCTCAGAACCTGTAACCAACTCACCTGCAAATTTGAGCAGC
rabbit    ------------------------------------------------------------

human     ACCTCAGTTGAAATACTGGCCACCTCTGAAGTCACCACAGATACAGAGAAAACTCATCCT
rabbit    ------------------------------------------------------------

human     TCTTCAAACAGAACAGTGACCGATGTGGGGACCTCCAGTTCTGGACATGAATCCACTTCC
rabbit    ------------------------------------------------------------

human     TTTGTCCTAGCTGACTCACAGACATCCAAAGTCACATCTCCAATGGTTATTACCTCCACC
rabbit    ------------------------------------------------------------

human     ATGGAGGATACGAGTGTCTCCACATCAACTCCTGGCTTTTTTGAGACTAGCAGAATTCAG
rabbit    ------------------------------------------------------------

human     ACAGAACCAACATCCTCCCTGACCCTTGGACTGAGAAAGACCAGCAGCTCTGAGGGGACC
rabbit    ------------------------------------------------------------

human     AGCTTAGCCACAGAGATGAGCACTGTCCTTTCTGGAGTGCCCACTGGTGCCACTGCTGAA
rabbit    ------------------------------------------------------------

human     GTCTCCAGGACAGAAGTCACCTCCTCTAGCAGAACATCCATCTCAGGCTTTGCTCAGCTC
rabbit    ------------------------------------------------------------

human     ACAGTGTCACCAGAGACTTCCACAGAAACCATCACCAGACTCCCTACCTCCAGCATAATG
rabbit    ------------------------------------------------------------
```

```
human     ACAGAATCAGCAGAAATGATGATCAAGACACAAACAGATCCTCCTGGGTCTACACCAGAG
rabbit    ------------------------------------------------------------

human     AGTACTCATACTGTGGACATATCAACAACACCCAACTGGGTAGAAACCCACTCGACTGTG
rabbit    ------------------------------------------------------------

human     ACTCAGAGATTTTCACACTCAGAGATGACCACTCTTGTGAGCAGAAGCCCTGGTGATATG
rabbit    ------------------------------------------------------------

human     TTATGGCCTAGTCAATCCTCTGTGGAAGAAACCAGCTCTGCCTCTTCCCTGCTGTCTCTG
rabbit    ------------------------------------------------------------

human     CCTGCCACGACCTCACCTTCTCCTGTTTCCTCTACATTAGTAGAGGATTTCCCTTCCGCT
rabbit    ------------------------------------------------------------

human     TCTCTTCCTGTGACTTCTCTTCTCAACCCTGGCCTGGTGATAACCACAGACAGGATGGGC
rabbit    ------------------------------------------------------------

human     ATAAGCAGAGAACCTGGAACCAGTTCCACTTCAAATTTGAGCAGCACCTCCCATGAGAGA
rabbit    ------------------------------------------------------------

human     CTGACCACTTTGGAAGACACTGTAGATACAGAAGACATGCAGCCTTCCACACACACAGCA
rabbit    ------------------------------------------------------------

human     GTGACCAACGTGAGGACCTCCATTTCTGGACATGAATCACAATCTTCTGTCCTATCTGAC
rabbit    ------------------------------------------------------------
```

human TCAGAGACACCCAAAGCCACATCTCCAATGGGTACCACCTACACCATGGGGGAAACGAGT
rabbit --

human GTTTCCATATCCACTTCTGACTTCTTTGAGACCAGCAGAATTCAGATAGAACCAACATCC
rabbit --

human TCCCTGACTTCTGGATTGAGGGAGACCAGCAGCTCTGAGAGGATCAGCTCAGCCACAGAG
rabbit --

human GGAAGCACTGTCCTTTCTGAAGTGCCCAGTGGTGCTACCACTGAGGTCTCCAGGACAGAA
rabbit --

human GTGATATCCTCTAGGGGAACATCCATGTCAGGGCCTGATCAGTTCACCATATCACCAGAC
rabbit --

human ATCTCTACTGAAGCGATCACCAGGCTTTCTACTTCCCCCATTATGACAGAATCAGCAGAA
rabbit --

human AGTGCCATCACTATTGAGACAGGTTCTCCTGGGGCTACATCAGAGGGTACCCTCACCTTG
rabbit --

human GACACCTCAACAACAACCTTTTGGTCAGGGACCCACTCAACTGCATCTCCAGGATTTTCA
rabbit --

human CACTCAGAGATGACCACTCTTATGAGTAGAACTCCTGGAGATGTGCCATGGCCGAGCCTT
rabbit --

```
human     CCCTCTGTGGAAGAAGCCAGCTCTGTCTCTTCCTCACTGTCTTCACCTGCCATGACCTCA
rabbit    ------------------------------------------------------------

human     ACTTCTTTTTTCTCCACATTACCAGAGAGCATCTCCTCCTCTCCTCATCCTGTGACTGCA
rabbit    ------------------------------------------------------------

human     CTTCTCACCCTTGGCCCAGTGAAGACCACAGACATGTTGCGCACAAGCTCAGAACCTGAA
rabbit    ------------------------------------------------------------

human     ACCAGTTCACCTCCAAATTTGAGCAGCACCTCAGCTGAAATATTAGCCACGTCTGAAGTC
rabbit    ------------------------------------------------------------

human     ACCAAAGATAGAGAGAAAATTCATCCCTCCTCAAACACACCTGTAGTCAATGTAGGGACT
rabbit    ------------------------------------------------------------

human     GTGATTTATAAACATCTATCCCCTTCCTCTGTTTTGGCTGACTTAGTGACAACAAAACCC
rabbit    ------------------------------------------------------------

human     ACATCTCCAATGGCTACCACCTCCACTCTGGGGAATACAAGTGTTTCCACATCAACTCCT
rabbit    ------------------------------------------------------------

human     GCCTTCCCAGAAACTATGATGACACAGCCAACTTCCTCCCTGACTTCTGGATTAAGGGAG
rabbit    ------------------------------------------------------------

human     ATCAGTACCTCTCAAGAGACCAGCTCAGCAACAGAGAGAAGTGCTTCTCTTTCTGGAATG
rabbit    ------------------------------------------------------------
```

```
human     CCCACTGGTGCTACTACTAAGGTCTCCAGAACAGAAGCCCTCTCCTTAGGCAGAACATCC
rabbit    ------------------------------------------------------------

human     ACCCCAGGTCCTGCTCAATCCACAATATCACCAGAAATCTCCACGGAAACCATCACTAGA
rabbit    ------------------------------------------------------------

human     ATTTCTACTCCCCTCACCACGACAGGATCAGCAGAAATGACCATCACCCCCAAAACAGGT
rabbit    ------------------------------------------------------------

human     CATTCTGGGGCATCCTCACAAGGTACCTTTACCTTGGACACATCAAGCAGAGCCTCCTGG
rabbit    ------------------------------------------------------------

human     CCAGGAACTCACTCAGCTGCAACTCACAGATCTCCACACTCAGGGATGACCACTCCTATG
rabbit    ------------------------------------------------------------

human     AGCAGAGGTCCTGAGGATGTGTCATGGCCAAGCCGCCCATCAGTGGAAAAAACTAGCCCT
rabbit    ------------------------------------------------------------

human     CCATCTTCCCTGGTGTCTTTATCTGCAGTAACCTCACCTTCGCCACTTTATTCCACACCA
rabbit    ------------------------------------------------------------

human     TCTGAGAGTAGCCACTCATCTCCTCTCCGGGTGACTTCTCTTTTCACCCCTGTCATGATG
rabbit    ------------------------------------------------------------

human     AAGACCACAGACATGTTGGACACAAGCTTGGAACCTGTGACCACTTCACCTCCCAGTATG
rabbit    ------------------------------------------------------------
```

```
human           AATATCACCTCAGATGAGAGTCTGGCCACTTCTAAAGCCACCATGGAGACAGAGGCAATT
rabbit          ------------------------------------------------------------

human           CAGCTTTCAGAAAACACAGCTGTGACTCAGATGGGCACCATCAGCGCTAGACAAGAATTC
rabbit          ------------------------------------------------------------

human           TATTCCTCTTATCCAGGCCTCCCAGAGCCATCCAAAGTGACATCTCCAGTGGTCACCTCT
rabbit          ------------------------------------------------------------

human           TCCACCATAAAAGACATTGTTTCTACAACCATACCTGCTTCCTCTGAGATAACAAGAATT
rabbit          ------------------------------------------------------------

human           GAGATGGAGTCAACATCCACCCTGACCCCCACACCAAGGGAGACCAGCACCTCCCAGGAG
rabbit          ------------------------------------------------------------

human           ATCCACTCAGCCACAAAGCCAAGCACTGTTCCTTACAAGGCACTCACTAGTGCCACGATT
rabbit          ------------------------------------------------------------

human           GAGGACTCCATGACACAAGTCATGTCCTCTAGCAGAGGACCTAGCCCTGATCAGTCCACA
rabbit          ------------------------------------------------------------

human           ATGTCACAAGACATATCCACTGAAGTGATCACCAGGCTCTCTACCTCCCCCATCAAGACA
rabbit          ------------------------------------------------------------

human           GAATCTACAGAAATGACCATTACCACCCAAACAGGTTCTCCTGGGGCTACATCAAGGGGT
rabbit          ------------------------------------------------------------
```

```
human     ACCCTTACCTTGGACACTTCAACAACTTTTATGTCAGGGACCCACTCAACTGCATCTCAA
rabbit    ------------------------------------------------------------

human     GGATTTTCACACTCACAGATGACCGCTCTTATGAGTAGAACTCCTGGAGATGTGCCATGG
rabbit    ------------------------------------------------------------

human     CTAAGCCATCCCTCTGTGGAAGAAGCCAGCTCTGCCTCTTTCTCACTGTCTTCACCTGTC
rabbit    ------------------------------------------------------------

human     ATGACCTCATCTTCTCCCGTTTCTTCCACATTACCAGACAGCATCCACTCTTCTTCGCTT
rabbit    ------------------------------------------------------------

human     CCTGTGACATCACTTCTCACCTCAGGGCTGGTGAAGACCACAGAGCTGTTGGGCACAAGC
rabbit    ------------------------------------------------------------

human     TCAGAACCTGAAACCAGTTCACCCCCAAATTTGAGCAGCACCTCAGCTGAAATACTGGCC
rabbit    ------------------------------------------------------------

human     ATCACTGAAGTCACTACAGATACAGAGAAACTGGAGATGACCAATGTGGTAACCTCAGGT
rabbit    ------------------------------------------------------------

human     TATACACATGAATCTCCTTCCTCTGTCCTAGCTGACTCAGTGACAACAAAGGCCACATCT
rabbit    ------------------------------------------------------------

human     TCAATGGGTATCACCTACCCCACAGGAGATACAAATGTTCTCACATCAACCCCTGCCTTC
rabbit    ------------------------------------------------------------
```

```
human           TCTGACACCAGTAGGATTCAAACAAAGTCAAAGCTCTCACTGACTCCTGGGTTGATGGAG
rabbit          ------------------------------------------------------------

human           ACCAGCATCTCTGAAGAGACCAGCTCTGCCACAGAAAAAAGCACTGTCCTTTCTAGTGTG
rabbit          ------------------------------------------------------------

human           CCCACTGGTGCTACTACTGAGGTCTCCAGGACAGAAGCCATCTCTTCTAGCAGAACATCC
rabbit          ------------------------------------------------------------

human           ATCCCAGGCCCTGCTCAATCCACAATGTCATCAGACACCTCCATGGAAACCATCACTAGA
rabbit          ------------------------------------------------------------

human           ATTTCTACCCCCCTCACAAGGAAAGAATCAACAGACATGGCCATCACCCCCAAAACAGGT
rabbit          ------------------------------------------------------------

human           CCTTCTGGGGCTACCTCGCAGGGTACCTTTACCTTGGACTCATCAAGCACAGCCTCCTGG
rabbit          ------------------------------------------------------------

human           CCAGGAACTCACTCAGCTACAACTCAGAGATTTCCACAGTCAGTGGTGACAACTCCTATG
rabbit          ------------------------------------------------------------

human           AGCAGAGGTCCTGAGGATGTGTCATGGCCAAGCCCGCTGTCTGTGGAAAAAAACAGCCCT
rabbit          ------------------------------------------------------------

human           CCATCTTCCCTGGTATCTTCATCTTCAGTAACCTCACCTTCGCCACTTTATTCCACACCA
rabbit          ------------------------------------------------------------
```

```
human     TCTGGGAGTAGCCACTCCTCTCCTGTCCCTGTCACTTCTCTTTTCACCTCTATCATGATG
rabbit    ------------------------------------------------------------

human     AAGGCCACAGACATGTTGGATGCAAGTTTGGAACCTGAGACCACTTCAGCTCCCAATATG
rabbit    ------------------------------------------------------------

human     AATATCACCTCAGATGAGAGTCTGGCCGCTTCTAAAGCCACCACGGAGACAGAGGCAATT
rabbit    ------------------------------------------------------------

human     CACGTTTTTGAAAATACAGCAGCGTCCCATGTGGAAACCACCAGTGCTACAGAGGAACTC
rabbit    ------------------------------------------------------------

human     TATTCCTCTTCCCCAGGCTTCTCAGAGCCAACAAAAGTGATATCTCCAGTGGTCACCTCT
rabbit    ------------------------------------------------------------

human     TCCTCTATAAGAGACAACATGGTTTCCACAACAATGCCTGGCTCCTCTGGCATTACAAGG
rabbit    ------------------------------------------------------------

human     ATTGAGATAGAGTCAATGTCATCTCTGACCCCTGGACTGAGGGAGACCAGAACCTCCCAG
rabbit    ------------------------------------------------------------

human     GACATCACCTCATCCACAGAGACAAGCACTGTCCTTTACAAGATGCCCTCTGGTGCCACT
rabbit    ------------------------------------------------------------

human     CCTGAGGTCTCCAGGACAGAAGTTATGCCCTCTAGCAGAACATCCATTCCTGGCCCTGCT
rabbit    ------------------------------------------------------------
```

```
human          CAGTCCACAATGTCACTAGACATCTCCGATGAAGTTGTCACCAGGCTGTCTACCTCTCCC
rabbit         ------------------------------------------------------------

human          ATCATGACAGAATCTGCAGAAATAACCATCACCACCCAAACAGGTTATTCTCTGGCTACA
rabbit         ------------------------------------------------------------

human          TCCCAGGTTACCCTTCCCTTGGGCACCTCAATGACCTTTTTGTCAGGGACCCACTCAACT
rabbit         ------------------------------------------------------------

human          ATGTCTCAAGGACTTTCACACTCAGAGATGACCAATCTTATGAGCAGGGGTCCTGAAAGT
rabbit         ------------------------------------------------------------

human          CTGTCATGGACGAGCCCTCGCTTTGTGGAAACAACTAGATCTTCCTCTTCTCTGACATCA
rabbit         ------------------------------------------------------------

human          TTACCTCTCACGACCTCACTTTCTCCTGTGTCCTCCACATTACTAGACAGTAGCCCCTCC
rabbit         ------------------------------------------------------------

human          TCTCCTCTTCCTGTGACTTCACTTATCCTCCCAGGCCTGGTGAAGACTACAGAAGTGTTG
rabbit         ------------------------------------------------------------

human          GATACAAGCTCAGAGCCTAAAACCAGTTCATCTCCAAATTTGAGCAGCACCTCAGTTGAA
rabbit         ------------------------------------------------------------

human          ATACCGGCCACCTCTGAAATCATGACAGATACAGAGAAAATTCATCCTTCCTCAAACACA
rabbit         ------------------------------------------------------------
```

```
human     GCGGTGGCCAAAGTGAGGACCTCCAGTTCTGTTCATGAATCTCATTCCTCTGTCCTAGCT
rabbit    ------------------------------------------------------------

human     GACTCAGAAACAACCATAACCATACCTTCAATGGGTATCACCTCCGCTGTGGACGATACC
rabbit    ------------------------------------------------------------

human     ACTGTTTTCACATCAAATCCTGCCTTCTCTGAGACTAGGAGGATTCCGACAGAGCCAACA
rabbit    ------------------------------------------------------------

human     TTCTCATTGACTCCTGGATTCAGGGAGACTAGCACCTCTGAAGAGACCACCTCAATCACA
rabbit    ------------------------------------------------------------

human     GAAACAAGTGCAGTCCTTTATGGAGTGCCCACTAGTGCTACTACTGAAGTCTCCATGACA
rabbit    ------------------------------------------------------------

human     GAAATCATGTCCTCTAATAGAATACACATCCCTGACTCTGATCAGTCCACGATGTCTCCA
rabbit    ------------------------------------------------------------

human     GACATCATCACTGAAGTGATCACCAGGCTCTCTTCCTCATCCATGATGTCAGAATCAACA
rabbit    ------------------------------------------------------------

human     CAAATGACCATCACCACCCAAAAAAGTTCTCCTGGGGCTACAGCACAGAGTACTCTTACC
rabbit    ------------------------------------------------------------

human     TTGGCCACAACAACAGCCCCCTTGGCAAGGACCCACTCAACTGTTCCTCCTAGATTTTTA
rabbit    ------------------------------------------------------------
```

```
human      CACTCAGAGATGACAACTCTTATGAGTAGGAGTCCTGAAAATCCATCATGGAAGAGCTCT
rabbit     ------------------------------------------------------------

human      CTCTTTGTGGAAAAAACTAGCTCTTCATCTTCTCTGTTGTCCTTACCTGTCACGACCTCA
rabbit     ------------------------------------------------------------

human      CCTTCTGTTTCTTCCACATTACCGCAGAGTATCCCTTCCTCCTCTTTTTCTGTGACTTCA
rabbit     ------------------------------------------------------------

human      CTCCTCACCCCAGGCATGGTGAAGACTACAGACACAAGCACAGAACCTGGAACCAGTTTA
rabbit     ------------------------------------------------------------

human      TCTCCAAATCTGAGTGGCACCTCAGTTGAAATACTGGCTGCCTCTGAAGTCACCACAGAT
rabbit     ------------------------------------------------------------

human      ACAGAGAAAATTCATCCTTCTTCAAGCATGGCAGTGACCAATGTGGGAACCACCAGTTCT
rabbit     ------------------------------------------------------------

human      GGACATGAACTATATTCCTCTGTTTCAATCCACTCGGAGCCATCCAAGGCTACATACCCA
rabbit     ------------------------------------------------------------

human      GTGGGTACTCCCTCTTCCATGGCTGAAACCTCTATTTCCACATCAATGCCTGCTAATTTT
rabbit     ------------------------------------------------------------

human      GAGACCACAGGATTTGAGGCTGAGCCATTTTCTCATTTGACTTCTGGATTTAGGAAGACA
rabbit     ------------------------------------------------------------
```

```
human      AACATGTCCCTGGACACCAGCTCAGTCACACCAACAAATACACCTTCTTCTCCTGGGTCC
rabbit     ------------------------------------------------------------

human      ACTCACCTTTTACAGAGTTCCAAGACTGATTTCACCTCTTCTGCAAAAACATCATCCCCA
rabbit     ------------------------------------------------------------

human      GACTGGCCTCCAGCCTCACAGTATACTGAAATTCCAGTGGACATAATCACCCCCTTTAAT
rabbit     ------------------------------------------------------------

human      GCTTCTCCATCTATTACGGAGTCCACTGGGATAACCTCCTTCCCAGAATCCAGGTTTACT
rabbit     ------------------------------------------------------------

human      ATGTCTGTAACAGAAAGTACTCATCATCTGAGTACAGATTTGCTGCCTTCAGCTGAGACT
rabbit     ------------------------------------------------------------

human      ATTTCCACTGGCACAGTGATGCCTTCTCTATCAGAGGCCATGACTTCATTTGCCACCACT
rabbit     ------------------------------------------------------------

human      GGAGTTCCACGAGCCATCTCAGGTTCAGGTAGTCCATTCTCTAGGACAGAGTCAGGCCCT
rabbit     ------------------------------------------------------------

human      GGGGATGCTACTCTGTCCACCATTGCAGAGAGCCTGCCTTCATCCACTCCTGTGCCATTC
rabbit     ------------------------------------------------------------

human      TCCTCTTCAACCTTCACTACCACTGATTCTTCAACCATCCCAGCCCTCCATGAGATAACT
rabbit     ------------------------------------------------------------
```

```
human     TCCTCTTCAGCTACCCCATATAGAGTGGACACCAGTCTTGGGACAGAGAGCAGCACTACT
rabbit    ------------------------------------------------------------

human     GAAGGACGCTTGGTTATGGTCAGTACTTTGGACACTTCAAGCCAACCAGGCAGGACATCT
rabbit    ------------------------------------------------------------

human     TCATCACCCATTTTGGATACCAGAATGACAGAGAGCGTTGAGCTGGGAACAGTGACAAGT
rabbit    ------------------------------------------------------------

human     GCTTATCAAGTTCCTTCACTCTCAACACGGTTGACAAGAACTGATGGCATTATGGAACAC
rabbit    ------------------------------------------------------------

human     ATCACAAAAATACCCAATGAAGCAGCACACAGAGGTACCATAAGACCAGTCAAAGGCCCT
rabbit    ------------------------------------------------------------

human     CAGACATCCACTTCGCCTGCCAGTCCTAAAGGACTACACACAGGAGGGACAAAAAGAATG
rabbit    ------------------------------------------------------------

human     GAGACCACCACCACAGCTCTGAAGACCACCACCACAGCTCTGAAGACCACTTCCAGAGCC
rabbit    ------------------------------------------------------------

human     ACCTTGACCACCAGTGTCTATACTCCCACTTTGGGAACACTGACTCCCCTCAATGCATCA
rabbit    ------------------------------------------------------------

human     ATGCAAATGGCCAGCACAATCCCCACAGAAATGATGATCACAACCCCATATGTTTTCCCT
rabbit    ------------------------------------------------------------
```

```
human     GATGTTCCAGAAACGACATCCTCATTGGCTACCAGCCTGGGAGCAGAAACCAGCACAGCT
rabbit    ------------------------------------------------------------

human     CTTCCCAGGACAACCCCATCTGTTTTCAATAGAGAATCAGAGACCACAGCCTCACTGGTC
rabbit    ------------------------------------------------------------

human     TCTCGTTCTGGGGCAGAGAGAAGTCCGGTTATTCAAACTCTAGATGTTTCTTCTAGTGAG
rabbit    ------------------------------------------------------------

human     CCAGATACAACAGCTTCATGGGTTATCCATCCTGCAGAGACCATCCCAACTGTTTCCAAG
rabbit    ------------------------------------------------------------

human     ACAACCCCCAATTTTTTCCACAGTGAATTAGACACTGTATCTTCCACAGCCACCAGTCAT
rabbit    ------------------------------------------------------------

human     GGGGCAGACGTCAGCTCAGCCATTCCAACAAATATCTCACCTAGTGAACTAGATGCACTG
rabbit    ------------------------------------------------------------

human     ACCCCACTGGTCACTATTTCGGGGACAGATACTAGTACAACATTCCCAACACTGACTAAG
rabbit    ------------------------------------------------------------

human     TCCCCACATGAAACAGAGACAAGAACCACATGGCTCACTCATCCTGCAGAGACCAGCTCA
rabbit    ------------------------------------------------------------

human     ACTATTCCCAGAACAATCCCCAATTTTTCTCATCATGAATCAGATGCCACACCTTCAATA
rabbit    ------------------------------------------------------------
```

```
human     GCCACCAGTCCTGGGGCAGAAACCAGTTCAGCTATTCCAATTATGACTGTCTCACCTGGT
rabbit    ------------------------------------------------------------

human     GCAGAAGATCTGGTGACCTCACAGGTCACTAGTTCTGGGACAGACAGAAATATGACTATT
rabbit    ------------------------------------------------------------

human     CCAACTTTGACTCTTTCTCCTGGTGAACCAAAGACGATAGCCTCATTAGTCACCCATCCT
rabbit    ------------------------------------------------------------

human     GAAGCACAGACAAGTTCGGCCATTCCAACTTCAACTATCTCGCCTGCTGTATCACGGTTG
rabbit    ------------------------------------------------------------

human     GTGACCTCAATGGTCACCAGTTTGGCGGCAAAGACAAGTACAACTAATCGAGCTCTGACA
rabbit    ------------------------------------------------------------

human     AACTCCCCTGGTGAACCAGCTACAACAGTTTCATTGGTCACGCATCCTGCACAGACCAGC
rabbit    ------------------------------------------------------------

human     CCAACAGTTCCCTGGACAACTTCCATTTTTTTCCATAGTAAATCAGACACCACACCTTCA
rabbit    ------------------------------------------------------------

human     ATGACCACCAGTCATGGGGCAGAATCCAGTTCAGCTGTTCCAACTCCAACTGTTTCAACT
rabbit    ------------------------------------------------------------

human     GAGGTACCAGGAGTAGTGACCCCTTTGGTCACCAGTTCTAGGGCAGTGATCAGTACAACT
rabbit    ------------------------------------------------------------
```

```
human       ATTCCAATTCTGACTCTTTCTCCTGGTGAACCAGAGACCACACCTTCAATGGCCACCAGT
rabbit      ------------------------------------------------------------

human       CATGGGGAAGAAGCCAGTTCTGCTATTCCAACTCCAACTGTTTCACCTGGGGTACCAGGA
rabbit      ------------------------------------------------------------

human       GTGGTGACCTCTCTGGTCACTAGTTCTAGGGCAGTGACTAGTACAACTATTCCAATTCTG
rabbit      ------------------------------------------------------------

human       ACTTTTTCTCTTGGTGAACCAGAGACCACACCTTCAATGGCCACCAGTCATGGGACAGAA
rabbit      ------------------------------------------------------------

human       GCTGGCTCAGCTGTTCCAACTGTTTTACCTGAGGTACCAGGAATGGTGACCTCTCTGGTT
rabbit      ------------------------------------------------------------

human       GCTAGTTCTAGGGCAGTAACCAGTACAACTCTTCCAACTCTGACTCTTTCTCCTGGTGAA
rabbit      ------------------------------------------------------------

human       CCAGAGACCACACCTTCAATGGCCACCAGTCATGGGGCAGAAGCCAGCTCAACTGTTCCA
rabbit      ------------------------------------------------------------

human       ACTGTTTCACCTGAGGTACCAGGAGTGGTGACCTCTCTGGTCACTAGTTCTAGTGGAGTA
rabbit      ------------------------------------------------------------

human       AACAGTACAAGTATTCCAACTCTGATTCTTTCTCCTGGTGAACTAGAAACCACACCTTCA
rabbit      ------------------------------------------------------------
```

```
human       ATGGCCACCAGTCATGGGGCAGAAGCCAGCTCAGCTGTTCCAACTCCAACTGTTTCACCT
rabbit      ------------------------------------------------------------

human       GGGGTATCAGGAGTGGTGACCCCTCTGGTCACTAGTTCCAGGGCAGTGACCAGTACAACT
rabbit      ------------------------------------------------------------

human       ATTCCAATTCTAACTCTTTCTTCTAGTGAGCCAGAGACCACACCTTCAATGGCCACCAGT
rabbit      ------------------------------------------------------------

human       CATGGGGTAGAAGCCAGCTCAGCTGTTCTAACTGTTTCACCTGAGGTACCAGGAATGGTG
rabbit      ------------------------------------------------------------

human       ACCTCTCTGGTCACTAGTTCTAGAGCAGTAACCAGTACAACTATTCCAACTCTGACTATT
rabbit      ------------------------------------------------------------

human       TCTTCTGATGAACCAGAGACCACAACTTCATTGGTCACCCATTCTGAGGCAAAGATGATT
rabbit      ------------------------------------------------------------

human       TCAGCCATTCCAACTTTAGCTGTCTCCCCTACTGTACAAGGGCTGGTGACTTCACTGGTC
rabbit      ------------------------------------------------------------

human       ACTAGTTCTGGGTCAGAGACCAGTGCGTTTTCAAATCTAACTGTTGCCTCAAGTCAACCA
rabbit      ------------------------------------------------------------

human       GAGACCATAGACTCATGGGTCGCTCATCCTGGGACAGAAGCAAGTTCTGTTGTTCCAACT
rabbit      ------------------------------------------------------------
```

```
human      TTGACTGTCTCCACTGGTGAGCCGTTTACAAATATCTCATTGGTCACCCATCCTGCAGAG
rabbit     ------------------------------------------------------------

human      AGTAGCTCAACTCTTCCCAGGACAACCTCAAGGTTTTCCCACAGTGAATTAGACACTATG
rabbit     ------------------------------------------------------------

human      CCTTCTACAGTCACCAGTCCTGAGGCAGAATCCAGCTCAGCCATTTCAACAACTATTTCA
rabbit     ------------------------------------------------------------

human      CCTGGTATACCAGGTGTGCTGACATCACTGGTCACTAGCTCTGGGAGAGACATCAGTGCA
rabbit     ------------------------------------------------------------

human      ACTTTTCCAACAGTGCCTGAGTCCCCACATGAATCAGAGGCAACAGCCTCATGGGTTACT
rabbit     ------------------------------------------------------------

human      CATCCTGCAGTCACCAGCACAACAGTTCCCAGGACAACCCCTAATTATTCTCATAGTGAA
rabbit     ------------------------------------------------------------

human      CCAGACACCACACCATCAATAGCCACCAGTCCTGGGGCAGAAGCCACTTCAGATTTTCCA
rabbit     ------------------------------------------------------------

human      ACAATAACTGTCTCACCTGATGTACCAGATATGGTAACCTCACAGGTCACTAGTTCTGGG
rabbit     ------------------------------------------------------------

human      ACAGACACCAGTATAACTATTCCAACTCTGACTCTTTCTTCTGGTGAGCCAGAGACCACA
rabbit     ------------------------------------------------------------
```

```
human      ACCTCATTTATCACCTATTCTGAGACACACACAAGTTCAGCCATTCCAACTCTCCCTGTC
rabbit     ------------------------------------------------------------

human      TCCCCTGGTGCATCAAAGATGCTGACCTCACTGGTCATCAGTTCTGGGACAGACAGCACT
rabbit     ------------------------------------------------------------

human      ACAACTTTCCCAACACTGACGGAGACCCCATATGAACCAGAGACAACAGCCATACAGCTC
rabbit     ------------------------------------------------------------

human      ATTCATCCTGCAGAGACCAACACAATGGTTCCCAGGACAACTCCCAAGTTTTCCCATAGT
rabbit     ------------------------------------------------------------

human      AAGTCAGACACCACACTCCCAGTAGCCATCACCAGTCCTGGGCCAGAAGCCAGTTCAGCT
rabbit     ------------------------------------------------------------

human      GTTTCAACGACAACTATCTCACCTGATATGTCAGATCTGGTGACCTCACTGGTCCCTAGT
rabbit     ------------------------------------------------------------

human      TCTGGGACAGACACCAGTACAACCTTCCCAACATTGAGTGAGACCCCATATGAACCAGAG
rabbit     ------------------------------------------------------------

human      ACTACAGCCACGTGGCTCACTCATCCTGCAGAAACCAGCACAACGGTTTCTGGGACAATT
rabbit     ------------------------------------------------------------

human      CCCAACTTTTCCCATAGGGGATCAGACACTGCACCCTCAATGGTCACCAGTCCTGGAGTA
rabbit     ------------------------------------------------------------
```

```
human           GACACGAGGTCAGGTGTTCCAACTACAACCATCCCACCCAGTATACCAGGGGTAGTGACC
rabbit          ------------------------------------------------------------

human           TCACAGGTCACTAGTTCTGCAACAGACACTAGTACAGCTATTCCAACTTTGACTCCTTCT
rabbit          ------------------------------------------------------------

human           CCTGGTGAACCAGAGACCACAGCCTCATCAGCTACCCATCCTGGGACACAGACTGGCTTC
rabbit          ------------------------------------------------------------

human           ACTGTTCCAATTCGGACTGTTCCCTCTAGTGAGCCAGATACAATGGCTTCCTGGGTCACT
rabbit          ------------------------------------------------------------

human           CATCCTCCACAGACCAGCACACCTGTTTCCAGAACAACCTCCAGTTTTTCCCATAGTAGT
rabbit          ------------------------------------------------------------

human           CCAGATGCCACACCTGTAATGGCCACCAGTCCTAGGACAGAAGCCAGTTCAGCTGTACTG
rabbit          ------------------------------------------------------------

human           ACAACAATCTCACCTGGTGCACCAGAGATGGTGACTTCACAGATCACTAGTTCTGGGGCA
rabbit          ------------------------------------------------------------

human           GCAACCAGTACAACTGTTCCAACTTTGACTCATTCTCCTGGTATGCCAGAGACCACAGCC
rabbit          ------------------------------------------------------------

human           TTATTGAGCACCCATCCCAGAACAGAGACAAGTAAAACATTTCCTGCTTCAACTGTGTTT
rabbit          ------------------------------------------------------------
```

```
human      CCTCAAGTATCAGAGACCACAGCCTCACTCACCATTAGACCTGGTGCAGAGACTAGCACA
rabbit     ------------------------------------------------------------

human      GCTCTCCCAACTCAGACAACATCCTCTCTCTTCACCCTACTTGTAACTGGAACCAGCAGA
rabbit     ------------------------------------------------------------

human      GTTGATCTAAGTCCAACTGCTTCACCTGGTGTTTCTGCAAAAACAGCCCCACTTTCCACC
rabbit     ------------------------------------------------------------

human      CATCCAGGGACAGAAACCAGCACAATGATTCCAACTTCAACTCTTTCCCTTGGTTTACTA
rabbit     ------------------------------------------------------------

human      GAGACTACAGGCTTACTGGCCACCAGCTCTTCAGCAGAGACCAGCACGAGTACTCTAACT
rabbit     ------------------------------------------------------------

human      CTGACTGTTTCCCCTGCTGTCTCTGGGCTTTCCAGTGCCTCTATAACAACTGATAAGCCC
rabbit     ------------------------------------------------------------

human      CAAACTGTGACCTCCTGGAACACAGAAACCTCACCATCTGTAACTTCAGTTGGACCCCCA
rabbit     ------------------------------------------------------------

human      GAATTTTCCAGGACTGTCACAGGCACCACTATGACCTTGATACCATCAGAGATGCCAACA
rabbit     ------------------------------------------------------------

human      CCACCTAAAACCAGTCATGGAGAAGGAGTGAGTCCAACCACTATCTTGAGAACTACAATG
rabbit     ------------------------------------------------------------
```

```
human           GTTGAAGCCACTAATTTAGCTACCACAGGTTCCAGTCCCACTGTGGCCAAGACAACAACC
rabbit          ------------------------------------------------------------

human           ACCTTCAATACACTGGCTGGAAGCCTCTTTACTCCTCTGACCACACCTGGGATGTCCACC
rabbit          ------------------------------------------------------------

human           TTGGCCTCTGAGAGTGTGACCTCAAGAACAAGTTATAACCATCGGTCCTGGATCTCCACC
rabbit          ------------------------------------------------------------

human           ACCAGCAGTTATAACCGTCGGTACTGGACCCCTGCCACCAGCACTCCAGTGACTTCTACA
rabbit          ------------------------------------------------------------

human           TTCTCCCCAGGGATTTCCACATCCTCCATCCCCAGCTCCACAGCAGCCACAGTCCCATTC
rabbit          ------------------------------------------------------------

human           ATGGTGCCATTCACCCTCAACTTCACCATCACCAACCTGCAGTACGAGGAGGACATGCGG
rabbit          ------------------------------------------------------------

human           CACCCTGGTTCCAGGAAGTTCAACGCCACAGAGAGAGAACTGCAGGGTCTGCTCAAACCC
rabbit          ------------------------------------------------------------

human           TTGTTCAGGAATAGCAGTCTGGAATACCTCTATTCAGGCTGCAGACTAGCCTCACTCAGG
rabbit          ------------------------------------------------------------

human           CCAGAGAAGGATAGCTCAGCCACGGCAGTGGATGCCATCTGCACACATCGCCCTGACCCT
rabbit          ------------------------------------------------------------
```

```
human       GAAGACCTCGGACTGGACAGAGAGCGACTGTACTGGGAGCTGAGCAATCTGACAAATGGC
rabbit      ------------------------------------------------------------

human       ATCCAGGAGCTGGGCCCCTACACCCTGGACCGGAACAGTCTCTATGTCAATGGTTTCACC
rabbit      ------------------------------------------------------------

human       CATCGAAGCTCTATGCCCACCACCAGCACTCCTGGGACCTCCACAGTGGATGTGGGAACC
rabbit      ------------------------------------------------------------

human       TCAGGGACTCCATCCTCCAGCCCCAGCCCCACGACTGCTGGCCCTCTCCTGATGCCGTTC
rabbit      ------------------------------------------------------------

human       ACCCTCAACTTCACCATCACCAACCTGCAGTACGAGGAGGACATGCGTCGCACTGGCTCC
rabbit      ------------------------------------------------------------

human       AGGAAGTTCAACACCATGGAGAGTGTCCTGCAGGGTCTGCTCAAGCCCTTGTTCAAGAAC
rabbit      ------------------------------------------------------------

human       ACCAGTGTTGGCCCTCTGTACTCTGGCTGCAGATTGACCTTGCTCAGGCCCGAGAAAGAT
rabbit      ------------------------------------------------------------

human       GGGGCAGCCACTGGAGTGGATGCCATCTGCACCCACCGCCTTGACCCCAAAAGCCCTGGA
rabbit      ------------------------------------------------------------

human       CTCAACAGGGAGCAGCTGTACTGGGAGCTAAGCAAACTGACCAATGACATTGAAGAGCTG
rabbit      ------------------------------------------------------------
```

```
human     GGCCCCTACACCCTGGACAGGAACAGTCTCTATGTCAATGGTTTCACCCATCAGAGCTCT
rabbit    ------------------------------------------------------------

human     GTGTCCACCACCAGCACTCCTGGGACCTCCACAGTGGATCTCAGAACCTCAGGGACTCCA
rabbit    ------------------------------------------------------------

human     TCCTCCCTCTCCAGCCCCACAATTATGGCTGCTGGCCCTCTCCTGGTACCATTCACCCTC
rabbit    ------------------------------------------------------------

human     AACTTCACCATCACCAACCTGCAGTATGGGGAGGACATGGGTCACCCTGGCTCCAGGAAG
rabbit    ------------------------------------------------------------

human     TTCAACACCACAGAGAGGGTCCTGCAGGGTCTGCTTGGTCCCATATTCAAGAACACCAGT
rabbit    ------------------------------------------------------------

human     GTTGGCCCTCTGTACTCTGGCTGCAGACTGACCTCTCTCAGGTCTGAGAAGGATGGAGCA
rabbit    ------------------------------------------------------------

human     GCCACTGGAGTGGATGCCATCTGCATCCATCATCTTGACCCCAAAAGCCCTGGACTCAAC
rabbit    ------------------------------------------------------------

human     AGAGAGCGGCTGTACTGGGAGCTGAGCCAACTGACCAATGGCATCAAAGAGCTGGGCCCC
rabbit    ------------------------------------------------------------

human     TACACCCTGGACAGGAACAGTCTCTATGTCAATGGTTTCACCCATCGGACCTCTGTGCCC
rabbit    ------------------------------------------------------------
```

```
human           ACCAGCAGCACTCCTGGGACCTCCACAGTGGACCTTGGAACCTCAGGGACTCCATTCTCC
rabbit          ------------------------------------------------------------

human           CTCCCAAGCCCCGCAACTGCTGGCCCTCTCCTGGTGCTGTTCACCCTCAACTTCACCATC
rabbit          ------------------------------------------------------------

human           ACCAACCTGAAGTATGAGGAGGACATGCATCGCCCTGGCTCCAGGAAGTTCAACACCACT
rabbit          ------------------------------------------------------------

human           GAGAGGGTCCTGCAGACTCTGCTTGGTCCTATGTTCAAGAACACCAGTGTTGGCCTTCTG
rabbit          ------------------------------------------------------------

human           TACTCTGGCTGCAGACTGACCTTGCTCAGGTCCGAGAAGGATGGAGCAGCCACTGGAGTG
rabbit          ------------------------------------------------------------

human           GATGCCATCTGCACCCACCGTCTTGACCCCAAAAGCCCTGGAGTGGACAGGGAGCAGCTA
rabbit          ------------------------------------------------------------

human           TACTGGGAGCTGAGCCAGCTGACCAATGGCATCAAAGAGCTGGGCCCCTACACCCTGGAC
rabbit          ------------------------------------------------------------

human           AGGAACAGTCTCTATGTCAATGGTTTCACCCATTGGATCCCTGTGCCCACCAGCAGCACT
rabbit          ------------------------------------------------------------

human           CCTGGGACCTCCACAGTGGACCTTGGGTCAGGGACTCCATCCTCCCTCCCCAGCCCCACA
rabbit          ------------------------------------------------------------
```

```
human          ACTGCTGGCCCTCTCCTGGTGCCGTTCACCCTCAACTTCACCATCACCAACCTGAAGTAC
rabbit         ------------------------------------------------------------

human          GAGGAGGACATGCATTGCCCTGGCTCCAGGAAGTTCAACACCACAGAGAGAGTCCTGCAG
rabbit         ------------------------------------------------------------

human          AGTCTGCTTGGTCCCATGTTCAAGAACACCAGTGTTGGCCCTCTGTACTCTGGCTGCAGA
rabbit         ------------------------------------------------------------

human          CTGACCTTGCTCAGGTCCGAGAAGGATGGAGCAGCCACTGGAGTGGATGCCATCTGCACC
rabbit         ------------------------------------------------------------

human          CACCGTCTTGACCCCAAAAGCCCTGGAGTGGACAGGGAGCAGCTATACTGGGAGCTGAGC
rabbit         ------------------------------------------------------------

human          CAGCTGACCAATGGCATCAAAGAGCTGGGTCCCTACACCCTGGACAGAAACAGTCTCTAT
rabbit         ------------------------------------------------------------

human          GTCAATGGTTTCACCCATCAGACCTCTGCGCCCAACACCAGCACTCCTGGGACCTCCACA
rabbit         ------------------------------------------------------------

human          GTGGACCTTGGGACCTCAGGGACTCCATCCTCCCTCCCCAGCCCTACATCTGCTGGCCCT
rabbit         ------------------------------------------------------------

human          CTCCTGGTGCCATTCACCCTCAACTTCACCATCACCAACCTGCAGTACGAGGAGGACATG
rabbit         ------------------------------------------------------------
```

```
human           CATCACCCAGGCTCCAGGAAGTTCAACACCACGGAGCGGGTCCTGCAGGGTCTGCTTGGT
rabbit          ------------------------------------------------------------

human           CCCATGTTCAAGAACACCAGTGTCGGCCTTCTGTACTCTGGCTGCAGACTGACCTTGCTC
rabbit          ------------------------------------------------------------

human           AGGCCTGAGAAGAATGGGGCAGCCACTGGAATGGATGCCATCTGCAGCCACCGTCTTGAC
rabbit          ------------------------------------------------------------

human           CCCAAAAGCCCTGGACTCAACAGAGAGCAGCTGTACTGGGAGCTGAGCCAGCTGACCCAT
rabbit          ------------------------------------------------------------

human           GGCATCAAAGAGCTGGGCCCCTACACCCTGGACAGGAACAGTCTCTATGTCAATGGTTTC
rabbit          ------------------------------------------------------------

human           ACCCATCGGAGCTCTGTGGCCCCCACCAGCACTCCTGGGACCTCCACAGTGGACCTTGGG
rabbit          ------------------------------------------------------------

human           ACCTCAGGGACTCCATCCTCCCTCCCCAGCCCCACAACAGCTGTTCCTCTCCTGGTGCCG
rabbit          ------------------------------------------------------------

human           TTCACCCTCAACTTTACCATCACCAATCTGCAGTATGGGGAGGACATGCGTCACCCTGGC
rabbit          ------------------------------------------------------------

human           TCCAGGAAGTTCAACACCACAGAGAGGGTCCTGCAGGGTCTGCTTGGTCCCTTGTTCAAG
rabbit          ------------------------------------------------------------
```

```
human     AACTCCAGTGTCGGCCCTCTGTACTCTGGCTGCAGACTGATCTCTCTCAGGTCTGAGAAG
rabbit    ------------------------------------------------------------

human     GATGGGGCAGCCACTGGAGTGGATGCCATCTGCACCCACCACCTTAACCCTCAAAGCCCT
rabbit    ------------------------------------------------------------

human     GGACTGGACAGGGAGCAGCTGTACTGGCAGCTGAGCCAGATGACCAATGGCATCAAAGAG
rabbit    ------------------------------------------------------------

human     CTGGGCCCCTACACCCTGGACCGGAACAGTCTCTACGTCAATGGTTTCACCCATCGGAGC
rabbit    ------------------------------------------------------------

human     TCTGGGCTCACCACCAGCACTCCTTGGACTTCCACAGTTGACCTTGGAACCTCAGGGACT
rabbit    ------------------------------------------------------------

human     CCATCCCCCGTCCCCAGCCCCACAACCACCGGCCCTCTCCTGGTGCCATTCACACTCAAC
rabbit    ------------------------------------------------------------

human     TTCACCATCACTAACCTACAGTATGAGGAGAACATGGGTCACCCTGGCTCCAGGAAGTTC
rabbit    ------------------------------------------------------------

human     AACATCACGGAGAGTGTTCTGCAGGGTCTGCTCAAGCCCTTGTTCAAGAGCACCAGTGTT
rabbit    ------------------------------------------------------------

human     GGCCCTCTGTATTCTGGCTGCAGACTGACCTTGCTCAGGCCTGAGAAGGATGGAGTAGCC
rabbit    ------------------------------------------------------------
```

```
human     ACCAGAGTGGACGCCATCTGCACCCACCGCCCTGACCCCAAAATCCCTGGGCTAGACAGA
rabbit    ------------------------------------------------------------

human     CAGCAGCTATACTGGGAGCTGAGCCAGCTGACCCACAGCATCACTGAGCTGGGACCCTAC
rabbit    ------------------------------------------------------------

human     ACCCTGGATAGGGACAGTCTCTATGTCAATGGTTTCACCCAGCGGAGCTCTGTGCCCACC
rabbit    ------------------------------------------------------------

human     ACCAGCACTCCTGGGACTTTCACAGTACAGCCGGAAACCTCTGAGACTCCATCATCCCTC
rabbit    ------------------------------------------------------------

human     CCTGGCCCCACAGCCACTGGCCCTGTCCTGCTGCCATTCACCCTCAATTTTACCATCACT
rabbit    ------------------------------------------------------------

human     AACCTGCAGTATGAGGAGGACATGCGTCGCCCTGGCTCCAGGAAGTTCAACACCACGGAG
rabbit    ------------------------------------------------------------

human     AGGGTCCTTCAGGGTCTGCTTATGCCCTTGTTCAAGAACACCAGTGTCAGCTCTCTGTAC
rabbit    ------------------------------------------------------------

human     TCTGGTTGCAGACTGACCTTGCTCAGGCCTGAGAAGGATGGGGCAGCCACCAGAGTGGAT
rabbit    ------------------------------------------------------------

human     GCTGTCTGCACCCATCGTCCTGACCCCAAAAGCCCTGGACTGGACAGAGAGCGGCTGTAC
rabbit    ------------------------------------------------------------
```

```
human          TGGAAGCTGAGCCAGCTGACCCACGGCATCACTGAGCTGGGCCCCTACACCCTGGACAGG
rabbit         ------------------------------------------------------------

human          CACAGTCTCTATGTCAATGGTTTCACCCATCAGAGCTCTATGACGACCACCAGAACTCCT
rabbit         ------------------------------------------------------------

human          GATACCTCCACAATGCACCTGGCAACCTCGAGAACTCCAGCCTCCCTGTCTGGACCCATG
rabbit         ------------------------------------------------------------

human          ACCGCCAGCCCTCTCCTGGTGCTATTCACAATTAACTTCACCATCACTAACCTGCGGTAT
rabbit         ------------------------------------------------------------

human          GAGGAGAACATGCATCACCCTGGCTCTAGAAAGTTTAACACCACGGAGAGAGTCCTTCAG
rabbit         ------------------------------------------------------------

human          GGTCTGCTCAGGCCTGTGTTCAAGAACACCAGTGTTGGCCCTCTGTACTCTGGCTGCAGA
rabbit         ------------------------------------------------------------

human          CTGACCTTGCTCAGGCCCAAGAAGGATGGGGCAGCCACCAAAGTGGATGCCATCTGCACC
rabbit         ------------------------------------------------------------

human          TACCGCCCTGATCCCAAAAGCCCTGGACTGGACAGAGAGCAGCTATACTGGGAGCTGAGC
rabbit         ------------------------------------------------------------

human          CAGCTGACCCACAGCATCACTGAGCTGGGCCCCTACACCCTGGACAGGGACAGTCTCTAT
rabbit         ------------------------------------------------------------
```

```
human           GTCAATGGTTTCACACAGCGGAGCTCTGTGCCCACCACTAGCATTCCTGGGACCCCCACA
rabbit          ------------------------------------------------------------

human           GTGGACCTGGGAACATCTGGGACTCCAGTTTCTAAACCTGGTCCCTCGGCTGCCAGCCCT
rabbit          ------------------------------------------------------------

human           CTCCTGGTGCTATTCACTCTCAACTTCACCATCACCAACCTGCGGTATGAGGAGAACATG
rabbit          ------------------------------------------------------------

human           CAGCACCCTGGCTCCAGGAAGTTCAACACCACGGAGAGGGTCCTTCAGGGCCTGCTCAGG
rabbit          ------------------------------------------------------------

human           TCCCTGTTCAAGAGCACCAGTGTTGGCCCTCTGTACTCTGGCTGCAGACTGACTTTGCTC
rabbit          ------------------------------------------------------------

human           AGGCCTGAAAAGGATGGGACAGCCACTGGAGTGGATGCCATCTGCACCCACCACCCTGAC
rabbit          ------------------------------------------------------------

human           CCCAAAAGCCCTAGGCTGGACAGAGAGCAGCTGTATTGGGAGCTGAGCCAGCTGACCCAC
rabbit          ------------------------------------------------------------

human           AATATCACTGAGCTGGGCCCCTATGCCCTGGACAACGACAGCCTCTTTGTCAATGGTTTC
rabbit          ------------------------------------------------------------

human           ACTCATCGGAGCTCTGTGTCCACCACCAGCACTCCTGGGACCCCCACAGTGTATCTGGGA
rabbit          ------------------------------------------------------------
```

```
human     GCATCTAAGACTCCAGCCTCGATATTTGGCCCTTCAGCTGCCAGCCATCTCCTGATACTA
rabbit    ------------------------------------------------------------

human     TTCACCCTCAACTTCACCATCACTAACCTGCGGTATGAGGAGAACATGTGGCCTGGCTCC
rabbit    ------------------------------------------------------------

human     AGGAAGTTCAACACTACAGAGAGGGTCCTTCAGGGCCTGCTAAGGCCCTTGTTCAAGAAC
rabbit    ------------------------------------------------------------

human     ACCAGTGTTGGCCCTCTGTACTCTGGCTGCAGGCTGACCTTGCTCAGGCCAGAGAAAGAT
rabbit    -CCAGTGTTGGCACCTTCTACACTGGCTGCAGCCTGGCCGCTCTCAGCCTTGAGAAGGGA
           *********** *  * *** ********** *** **   ***** *  ***** *

human     GGGGAAGCCACCGGAGTGGATGCCATCTGCACCCACCGCCCTGACCCCACAGGCCCTGGG
rabbit    GGAGCAGCCACTGGTGTGAACTTTGTCTGCACTTTCCACTCTGACCCCTTAAACCCTGGA
          ** * ****** ** *** *     *******   ** * ********  *  ******

human     CTGGACAGAGAGCAGCTGTATTTGGAGCTGAGCCAGCTGACCCACAGCATCACTGAGCTG
rabbit    CTGCGCAGAGAGCAGCTGTACTGGGAGCTGAGCCGAGAGACCTATGGCATCACCCAGCTG
          ***  *************** * ***********    **** *  *******  *****

human     GGCCCCTACACACTGGACAGGGACAGTCTCTATGTCAATGGTTTCACCCATCGGAGCTCT
rabbit    GGCTCCTTTGCCCTGGACAGGGACAGTCTCTATGTCAATGATTACACCTATGGAGCCATG
          *** ***   * **************************** ** **** ** *   *

human     GTACCCACCACCAGCACCGGGGTGGTCAGCGAGGAGCCATTCACACTGAACTTCACCATC
rabbit    GCCCCAACTACCAGCACTGATGAGGTCAGTGAGGAGCCCTTCACGCTGAACTTCACCATC
          *  ** ** ******** *  * ****** ******** ***** ***************

human     AACAACCTGCGCTACATGGCGGACATGGGCCAACCCGGCTCCCTCAAGTTCAACATCACA
rabbit    AACAACCTGCGCTACTCTGCCAACATGGGCCGCCCTGGCTCCCTCAAGTTCAACATCACA
          ***************   **  *********  ** ************************
```

```
human           GACAACGTCATGCAGCACCTGCTCAGTCCTTTGTTCCAGAGGAGCAGCCTGGGTGCACGG
rabbit          GACACCCTCATGCAGCACCTGCTCAGCCCCTTGTTCCAGAGGAGCAGTCTGGGTGCCCGA
                **** * ******************* ** ***************** ******** **

human           TACACAGGCTGCAGGGTCATCGCACTAAGGTCTGTGAAGAACGGTGCTGAGACACGGGTG
rabbit          TATGCTGGCTGCAGGGTCATCATGCTAAGGTCTGTGAAGAACCATGCCCATACACAGGTG
                **  * ***************   ******************  ***  * **** ****

human           GACCTCCTCTGCACCTACCTGCAGCCCCTCAGCGGCCCAGGTCTGCCTATCAAGCAGGTG
rabbit          GACATCCTGTGTACCTACCGCTGGCCCCCTGGCAGCCCGCGGTTGTCTGCCAAGCGCGTG
                *** **** ** *******    *****   ** ****  *  ** **  *****  ***

human           TTCCATGAGCTGAGCCAGCAGACCCATGGCATCACCCGGCTGGGCCCCTACTCTCTGGAC
rabbit          TTCCATGAGCTGAGCTGGCAGACCCGAGGCATCACCCAGCTGGGCCCTTACTTCCTGGAC
                ***************  ********  ********** ********* ****  ******

human           AAAGACAGCCTCTACCTTAACGGTTACAATGAACCTGGTCCAGATGAGCCTCCTACAACT
rabbit          AAAGACAGCCTGCACATCGACGGTTACAATGAACGTGGGCCAGATGAGCCTCCTACAACT
                ***********  ** *  *************** *** *********************

human           CCCAAGCCAGCCACCACATTCCTGCCTCCTCTGTCA---------------GAAGCCACA
rabbit          CCTGAGCCAGCCACTGCATTACCGACTTCTCCATCAACATCTGTGTGGCCAGAAGCCACC
                **  **********  **** * * ** ***  ***               ********

human           ACAGCCATGGGGTACCACCTGAAGACCCTCACACTCAACTTCACCATCTCCAATCTCCAG
rabbit          ACAGCCACAGGGCACACCCTGAAGACCTTCACACTCAACTTCACCATCTCCAACCTCCAC
                *******  *** **  ********** ************************* *****

human           TATTCACCAGATATGGGCAAGGGCTCAGCTACATTCAACTCCACCGAGGGGGTCCTTCAG
rabbit          TATTCACCAGACATGGGCAACGGGTCAGCCACACTCAACTCCACTGAGCGGACCCTGCTG
                *********** ******** ** ***** *** ********** *** **  *** * *

human           CACCTGCTCAGACCCTTGTTCCAGAAGAGCAGCATGGGCCCCTTCTACTTGGGTTGCCAA
rabbit          TATCTGCTCGGATCCATGTTCCAGAACAGCAGCCTGGGTCCCTCCTACTCAGGCTGCAGA
                 * ****** ** ** ********** ****** **** **** *****  ** ***  *
```

```
human      CTGATCTCCCTCAGGCCTGAGAAGGATGGGGCAGCCACTGGTGTGGACACCACCTGCACC
rabbit     CTGACCTCTCTCAGGTCTGAGCAGGACAGGGTGGGCACTAGCGTGGATGTCATCTGCACC
           **** *** ****** ***** ****  ***  * **** * *****   ** *******

human      TACCACCCTGACCCTGTGGGCCCCGGGCTGGACATACAGCAGCTTTACTGGGAGCTGAGT
rabbit     TATCGCCCTGACCCCACGCACCTGGTGCTGGATGTGCAGCAGCTGTATGGAGAGCTGAGC
           ** * *********   *  **  * ******  * ******** **  * ******** 

human      CAGCTGACCCATGGTGTCACCCAACTGGGCTTCTATGTCCTGGACAGGGATAGCCTCTTC
rabbit     CAGCTGACCCATGGTGTCACCCAGTTGGGCCTGTACAGCTTGGACAAGGACAGCCTATTT
           ***********************  ***** * **   * ****** *** ***** ** 

human      ATCAATGGCTATGCACCCCAGAATTTATCA---ATCCGGGGCGAGTACCAGATAAATTTC
rabbit     GTCAATGGCTATGCACCCCAGCACGTGGCACCCATCTGGAGCGAGTACCAGCTTGATTTC
            ******************** *  *  **   *** ** *********** *  *****

human      CACATTGTCAACTGGAACCTCAGTAATCCAGACCCCACATCCTCAGAGTACATCACCCTG
rabbit     CACATAATCAACTGGAACCTCAGCAAGGCAGATCCCAGCTCTGTGGAGTACCTTGCTCTG
           *****  **************** **  **** ****  **    ****** *  * ***

human      CTGAGGGACATCCAGGACAAGGTCACCACACTCTACAAAGGCAGTCAACTACATGACACA
rabbit     ATGACGGACATCCAGGACAAGGTCACCAGACTCTACACAGGGAGTCAACTACGAGACGTG
            *** *********************** ******** *** **********  ***   

human      TTCCGCTTCTGCCTGGTCACCAACTTGACGATGGACTCCGTGTTGGTCACTGTCAAGGCA
rabbit     TTCAACTCATGCCTAGTCACCAACTTAACGTCGGGCTCTCTGTTGGTCACTATGAAAGCA
           ***  **  ***** *********** ***  ** ***  *********** * ** ***

human      TTGTTCTCCTCCAATTTGGACCCCAGCCTGGTGGAGCAAGTCTTTCTAGATAAGACCCTG
rabbit     CTGTTCTCCTCCAATCTGGACCCCAACCTGGTGAAGCAAGTTTTTCTAGAAAAGACCTCG
            ************** ********* ******* ******* ******** ******  *

human      AATGCCTCATTCCATTGGCTGGGCTCCACCTACCAGTTGGTGGACATCCATGTGACAGAA
rabbit     AATGCCTCATCCTACTGGCTGGGCGCCACCTACCAGTTGATGGATCTGCATGTGACAGAA
           ********** * * ********* ************** ****  * ************
```

```
human     ATGGAGTCATCAGTTTATC------------AACCAACAAGCAGCTCCAGCACCCAGCAC
rabbit    GTGAGGCCAGCAGTTCATCTACCTACAGAAAAACCAACAAGAAGTCCCAGTTCCCAGCAA
           **  * ** ***** ***            ********** **  ****  *******

human     TTCTACCTGAATTTCACCATCACCAACCTACCATATTCCCAGGACAAAGCCCAGCCAGGC
rabbit    TTCCAGCTGAATTTCACCATCACCAACCTGTTCTACTCCCAAGACATAGCCCAGGAAGGC
          *** * ***********************    ** ***** **** *******  ****

human     ACCACCAATTACCAGAGGAACAAAAGGAATATTGAGGATGCGCTCAACCAACTCTTCCGA
rabbit    AGTGCCAAATACCAGTGGAACAAAAGAAGTATTGAGGACGCGCTCAACCAGCTCTTCCAA
          *   **** ****** ********** * ********* *********** ******* *

human     AACAGCAGCATCAAGAGTTATTTTTCTGACTGTCAAGTTTCAACATTCAGGTCTGTCCCC
rabbit    AACAGCAGCGTCAAGAGTTACTTTTCCGAATGTCAAGTTGTAGCATTCCGGTCTGTCCCC
          ********* ********** ***** ** *********  * ***** ***********

human     AACAGGCACCACACCGGGGTGGACTCCCTGTGTAACTTCTCGCCACTGGCTCGGAGAGTA
rabbit    GACAGCGACCATACCAAAGTGGACTCCTTGTGTGGCTTCTCATCTTTGGCTCGGAGACTG
           ****  **** ***   ********* *****  ******  *  *********** *

human     GACAGAGTTGCCATCTATGAGGAATTTCTGCGGATGACCCGGAATGGTACCCAGCTGCAG
rabbit    GACAGAGTCATTATCTATGAGGAGTTTGTGCAGATGACCAAGAATGGTACTCAGTTGCAG
          ********    *********** *** *** *******  ********* *** *****

human     AACTTCACCCTGGACAGGAGCAGTGTCCTTGTGGATGGGTATTCTCCCAACAGAAATGAG
rabbit    AACTTCACCCTGGACAGGGACAGCATTCTTGTGGACGGGTATTCTCCAAGCAGAAATGAT
          ******************  ***  * ******** *********** * *********

human     CCCTTAACTGGGAATTCTGACCTTCCCTTCTGGGCTGTCATCCTCATCGGCTTGGCAGGA
rabbit    GCTGTGATTAGGAATTCAGACCTTCCCTTCTGGGCCATCATCCTCATCTGCCTGGCTGCA
           *  * * * ******* *****************  *********** ** **** * *

human     CTCCTGGGAGTCATCACATGCCTGATCTGCGGTGTCCTGGTGACCACCCGCCGGCGGAAG
rabbit    TTCCTGGGACTCATCACGTGCCTGGTCTGCTGCTTTCTGGTGGCCACCCGCCTGCGGAGG
           ******** ******* ****** ***** *  * ****** ********* ***** *
```

```
human      AAGGAAGGAGAATACAACGTCCAGCAACAGTGCCCAGGCTACTACCAGTCACACCTAGAC
rabbit     AAGGAGGGAGACTACGAGGTCCAACGCCGCCGCCTGGGCTACTATCTGCCACAGCTGGAC
           ***** ***** *** * ***** *  *   ***  ******** * * **** ** ***

human      CTGGAGGATCTGCAATGA---CTGGAACTTGCCGG---TGCCTGGGGTGCCTTTCCCCCA
rabbit     CTGACGAAGCTGCAGTAATTCCTGGAACCACAGGAGCTTGGCTGGAGCAGAAATAAACCA
           ***  * * ***** * *   *******     *    ** **** *      *   ***

human      GCCAGGGTCCAAAGAAGCTT--GGCTGGGGCAGAAATAAACCAT-ATTGGTCGGA-----
rabbit     CACTGGTGCGACACAGCCTCTGGGCCTTTCTTGACTTGGCCCCTGACTGGCTTGCAGAGA
             * **  * * * *  **   ***       **  *   ** * * ***   *

human      ------------------------------------------------------------
rabbit     CTACGAGGAAAGTTGGAAAGCCTTCCTTCCTTACTAAGCCCTGAAGTCAACTATCAGTTG

human      ------------------------------------------------------------
rabbit     TGTTTTATGGTTTCACACCATGTCACCCTTCAGGGATGCCAGGATTGCCATCACATAAAA

human      ------------------------------------------------------------
rabbit     TCAACATCCCACTTCACCATGAGGTTTTCCCTCCTGTTCACCCACTGTATACCTGCTATT

human      ------------------------------------------------------------
rabbit     CTCCCCATCACACCTATGTCTGCTCCTCCCCATTTCCCTACTTCCTGTAGTCCCCGTAGT

human      ------------------------------------------------------------
rabbit     CTTGAGTTTGTCTTCCTTCATTCTCTGAAGCTCAGGTTCACCAGGATTTCTGGATCAGTT

human      ------------------------------------------------------------
rabbit     TTCCTGGATGTCTCCTTACCCACCCCCATGGATTCTCCAAGATGAAAAACAGACCAAGAA
```

```
human         ------------------------------------------------------------
rabbit        GTGGCTCAAAAATGGCTCTGTAATCAGCTTTGGGACTGGAGAGCTGAAATGAAGAGAGGC

human         ------------------------------------------------------------
rabbit        ATTTAGGAAGCCAAGTAATATCAAGATTCTAGAAGCATAATGTCTGCTTTGGATATAAAG

human         ------------
rabbit        ATGAATAAAAGT
```

Underline fields are primer sequences we used.

Supplemental Information 5.

CLUSTAL 2.1 multiple sequence alignment

Galectin 3

```
human           GAGTATTTGAGGCTCGGAGCCACCGCCCCGCCGGCGCCCGCAGCACCTCCTCGCCAGCAG
rabbit          ------------------------------------------------------------


human           CCGTCCGGAGCCAGCCAACGAGCGGAAAATGGCAGACAATTTTTCGCTCCATGATGCGTT
rabbit          ----------------------AGGAAAATGGCGGATGGTTTTTCGCTCAACGATGCCCT
                                       ********** **   ********** * *****  *

human           ATCTGGGTCTGGAAACCCAAACCCTCAAGGATGGCCTGGCGCATGGGGGAACCAGCCTGC
rabbit          ATCTGGGTCTGGACACCCCCCAAACCAAGGATGGCCTGGCCCATGGGGGAACCAGCCTGC
                ************* ****       *************** *******************

human           TGGGGCAGGGGGCTACCCAGGGGCTTCCTATCCTGGGGCCTACCCCGGGCAGGCACCCCC
rabbit          TGGGCCAGGGGGCTACCCAGGGGCAGCCTATCCTGGGGCCTACCCTGGACATGCACCT--
                **** *******************  ******************* ** ** *****

human           AGGGGCTTATCCTGGACAGGCACCTCCAGGCGCCTACCCTGGAGCACCTGGAGCTTATCC
rabbit          -GGAGCTTATCCCGGGCAAGCGCCTCCTGGCCCCTACCCTGG----------------CC
                 ** ******** ** ** ** ***** *** **********                **

human           CGGAGCACCTGCACCTGGAGTCTACCCAGGGCCACCCAGCGGCCCTGGGGCCTACCCATC
rabbit          CAGGA-----GCACATGGAGCCTACCCTGGGCAGCCAGGTGGTCCTGGGGCCTACCCGTC
                * *       **** ***** ****** ****  **  * ** ************** **

human           TTCTGGACAGCCAAGTGCCACCGGAGCCTACCCTGCCACTGGCCCCTATGGCGCCCCTGC
rabbit          TCCTGGACAGCCAAGTGGTGCTGGAGCTTACCCTGGCGCCAGCCCTTACAGCGCCTCTGC
                * ***************   * ***** ******* * *  **** **  ***** ****

human           TGGGCCACTGATTGTGCCTTATAACCTGCCTTTGCCTGGGGGAGTGGTGCCTCGCATGCT
rabbit          TGGACCACTGCCTGTGCCTTATGACCTGCCTCTGCCTGGAGGAGTCATGCCCCGCATGCT
```

```
                  *** ******  ********** ******** ******* *****  **** ********

human             GATAACAATTCTGGGCACGGTGAAGCCCAATGCAAACAGAATTGCTTTAGATTTCCAAAG
rabbit            GATAACGATTGTGGGCACGGTGAAGCCCAATGCAAACAGACTCGCTTTGGATTTCAAGAG
                  ****** *** ***************************** * ***** ****** * **

human             AGGGAATGATGTTGCCTTCCACTTTAACCCACGCTTCAATGAGAACAACAGGAGAGTCAT
rabbit            AGGGAATGACGTTGCCTTCCACTTTAACCCCCGCTTCAATGAGAACAACAGGAGAGTCAT
                  ********* ******************** *****************************

human             TGTTTGCAATACAAAGCTGGATAATAACTGGGGAAGGGAAGAAAGACAGTCGGTTTTCCC
rabbit            TGTCTGCAACACAAAGGTGGATAACAACTGGGGAAGGGAAGAAAGGCAGACGACTTTCCC
                  *** ***** ****** ******* ******************** *** **  ******

human             ATTTGAAAGTGGGAAACCATTCAAAATACAAGTACTGGTTGAACCTGACCACTTCAAGGT
rabbit            ATTTGAAATTGGTAAACCATTCAAAATACAAGTCCTGGTGGAGCCAGACCACTTCAAGGT
                  ******** *** ******************** ***** ** ** **************

human             TGCAGTGAATGATGCTCACTTGTTGCAGTACAATCATCGGGTTAAAAAACTCAATGAAAT
rabbit            TGCGGTCAATGATGCCCACTTGTTGCAGTACAATCATCGCATGAGAAACCTCAAGGAAAT
                  *** ** ******** ***********************  * * *** ***** *****

human             CAGCAAACTGGGAATTTCTGGTGACATAGACCTCACCAGTGCTTCATATACCATGATATA
rabbit            CAACAAGCTGGGAATTTCTGGTGACATACAACTCACCAGTGCTTCACATGCTATGATATA
                  ** *** ********************* * *************** ** * ********

human             ATCTGAAAGGGGCAGATTAAAAAAAAAAAAAGAATCTAAACCTTACATGTGTAAAGGTTT
rabbit            A-----------------------------------------------------------
                  *

human             CATGTTCACTGTGAGTGAAAATTTTTACATTCATCAATATCCCTCTTGTAAGTCATCTAC
rabbit            ------------------------------------------------------------

human             TTAATAAATATTACAGTGAATTACCTGTCTCAATATGTCAAAAAAAAAAAAAAAAAA
rabbit            ---------------------------------------------------------
```

Underline fields are primer sequences we used.
